# “Hyphae Intertwined, Biomolecules Co-Born”—New Polyketides Induction by Co-Culture of the Mangrove Endophytic Fungus *Phomopsis asparagi* DHS-48 and *Pestalotiopsis* sp. HHL-101 at Both Volatile and Non-Volatile Levels

**DOI:** 10.3390/md23120452

**Published:** 2025-11-26

**Authors:** Ting Feng, Xiaojing Li, Zhenyi Liang, Jing Xu

**Affiliations:** Collaborative Innovation Center of Ecological Civilization, School of Chemistry and Chemical Engineering, Hainan University, Haikou 570228, China

**Keywords:** mangrove endophytic fungi, *Phomopsis asparagi*, *Pestalotiopsis* sp., co-culture, chemical interaction, non-volatile metabolites, volatile metabolites

## Abstract

The co-culture technique, mimicking natural microbial interactions, has proven to be successful at activating silent biosynthetic gene clusters (BGCs) to produce novel metabolites or enhance the yield of specific metabolites. To effectively decode induction processes, it is critical to have a comprehensive understanding of intermicrobial interactions across both volatile and non-volatile metabolomes. As part of our attempt to uncover structurally unique and biologically active natural products from mangrove endophytic fungi, *Phomopsis asparagi* DHS-48 was co-cultured with another mangrove fungal strain, *Pestalotiopsis* sp. HHL-101. The competition interaction of the two strains was investigated using morphology and scanning electron microscopy (SEM), and it was discovered that the mycelia of the DHS-48 and HHL-101 compressed and tangled with each other in the co-culture system, forming an interwoven pattern. To profile volatile-mediated chemical interactions during fungal co-culture, headspace solid-phase microextraction gas chromatography mass spectrometry (HS-SPME-GC-MS) coupled with orthogonal partial least squares-discriminant analysis (OPLS-DA) was adopted. Meanwhile, non-volatile metabolites from both liquid and solid small-scale co-cultures were profiled via HPLC. Two new polyketides, named phaseolorin K (**1**) and pestaphthalide C (**7**), together with 11 known compounds (**2**–**6**, **8**–**13**), were characterized from solid-state co-cultivation extracts of these two titled strains. Their planar structures were established by analysis of HRMS, MS/MS, and NMR spectroscopic data, while absolute configurations were assigned using ECD calculations. Co-culture feeding experiments demonstrated that DHS-48 exerts antagonistic activity against HHL-101 through altering its hyphal morphology, which mediated enhanced biosynthesis of non-volatile antimicrobial metabolites **5** and **6**. Biological assays revealed that compounds **4**–**6** exhibited potent in vitro cytotoxicity against human cancer cell lines HeLa and HepG2, compared to the positive controls adriamycin and fluorouracil. Compound **2** moderately inhibited the proliferation of ConA-induced T and LPS-induced B murine spleen lymphocytes.

## 1. Introduction

Mangrove-derived microbes that have adapted to extreme environmental conditions like high salinity, elevated temperatures, high humidity, light constraints, and limited air are regarded as a reliable source of distinctive metabolites [1,2,3,4,5]. The exploration of mangrove-derived microbes for structurally novel secondary metabolites exhibiting diverse biological activities has emerged as a prominent focus in pharmaceutical research, exemplified by the clinical proteasome inhibitor salinosporamide A (Marizomib^®^) [6,7]. Continued exploration of mangrove microbial consortia is anticipated to yield a new wave of structurally unprecedented bioactive metabolites in the future.

Recent years have seen a significant rise in chemical investigations of mangrove-derived microorganisms, especially endophytic fungi. Notably, endophytic strains alone (over 250) account for 1090 (79%) of the 1300 new compounds discovered from mangrove fungi [8]. However, whole-genome sequencing has revealed a significant discrepancy in mangrove endophytic fungi: the number of biosynthetic gene clusters (BGCs) vastly exceeds the quantity of chemically characterized metabolites produced [9,10]. One hypothesis attributes limited metabolite production to fungal domestication during conventional laboratory cultivation, activating only a fraction of BGCs [11,12]. Alternatively, the absence of environmental stressors required to induce cryptic gene clusters may suppress the expression of specific metabolites [13,14].

In nature, fungi evolve sophisticated strategies, including rapid growth, stress adaptation, and chemical defense mechanisms, to compete with other microorganisms [15,16]. The biosynthesis of defensive compounds is believed to serve an inhibitory function in competitive contexts, evidenced by observed biological activities including cytotoxicity [17] and antimicrobial activity manifested as the inhibition of colony formation [18]. To activate their silent biosynthetic gene clusters and access this untapped metabolic potential, multiple approaches were employed: epigenetic modifiers that alter gene expression, OSMAC (One Strain Many Compounds) techniques manipulating culture conditions, and genetic engineering targeting regulatory pathways. Among these, co-cultivation stands out as a particularly effective method [10]. By mimicking ecological competition through controlled fungal–microbial interactions, this technique directly triggers cryptic pathway activation [19]. Consequently, the technique not only reliably induces novel bioactive metabolites but also offers significantly lower technical barriers [18], making it the most widely adopted approach for activating silent genes and accelerating bioactive compounds discovery.

Fungal co-cultivation has arisen as a promising strategy for activating latent biosynthetic pathways and discovering novel bioactive compounds. Understanding the molecular mechanisms underlying intermicrobial interactions is crucial for effectively emphasizing these induction processes. Such interactions can be categorized into direct physical contact and indirect chemical signaling, each playing distinct roles in metabolic activation [20]. Direct interactions involve physical contact between co-cultured fungi that facilitates metabolic crosstalk through cell–cell communication, which enables the exchange of cytoplasmic contents, triggering stress-responsive biosynthetic pathways essential for activating novel metabolite production [14,21]. Indirect interactions occur via chemical communication mediated by volatile and non-volatile secondary metabolites, creating complex inter- and intra-kingdom signaling networks [22,23]. Microbial volatile organic compounds (MVOCs), chemically diverse metabolites readily transported through the air, play a significant role in these interactions [22,24,25]. Although numerous metabolomics analyses have elucidated non-volatile metabolites and their metabolic pathways, studies investigating the MVOCs generated during co-culture are exceedingly limited.

As part of our attempt to uncover structurally unique and biologically active natural compounds from mangrove endophytic fungi, we identified *Phomopsis asparagi* DHS-48, a strain isolated from the rhizomes of *Rhizophora mangle*. This fungal isolate demonstrated exceptional biosynthetic capacity, accumulating a series of chromone derivatives and cytochalasin congeners with significant immunomodulatory activity, including inhibition of T-cell proliferation and cytokine modulation [26,27,28]. To harness the metabolic potential of this fungal strain, we conducted co-culture fermentation of the well-studied DHS-48 with other fungi. Our previous work on co-culturing DHS-48 with a *Phomopsis* genus fungus DHS-11 led to the isolation of 23 metabolites (6 new), including nine dimeric xanthones, six alkaloids, two sterols, and six polyketides, while also enhancing the yields of key target compounds [29,30]. These findings established co-cultivation as an effective strategy for expanding the chemodiversity of DHS-48, which prompted us to co-cultivate DHS-48 with additional mangrove-derived fungi to further characterize its metabolic potential. Another mangrove endophytic fungus *Pestalotiopsis* sp. HHL-101 was selected, prioritized for two key reasons: (i) its documented production of structurally diverse bioactive metabolites, notably the immunosuppressant pestalotiopyrone M [31] and antibacterial pestalotiopisorin B [32]; and (ii) its shared evolutionary adaptation with Rhizophoraceae endophytes (HHL-101 from *R. stylosa vs. DHS-48* from *R. apiculata*; collected in the same conservation zone), suggesting that chemically compatible interactions may stimulate cryptic pathways in DHS-48 through interspecies chemical crosstalk.

**Figure 1:**
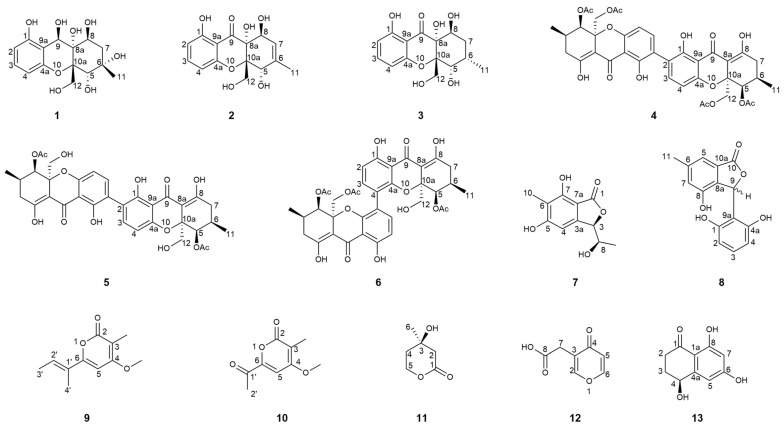
Structures of the isolated compounds **1**–**13**.

To elucidate the direct physical interaction between DHS-48 and HHL-101, we systematically investigated interactions between DHS-48 and HHL-101 through the integrated morphological characterization and metabolomic profiling of volatile/non-volatile metabolites. Physical interactions were investigated via scanning electron microscopy (SEM) of mycelial morphology on PDA co-culture plates, while volatile metabolite exchanges were analyzed using headspace solid-phase microextraction gas chromatography mass spectrometry (HS-SPME-GC-MS) coupled with OPLS-DA multivariate analysis. Non-volatile metabolites were investigated through HPLC screening of EtOAc extracts from small-scale liquid/solid fermentations to optimize upscaled co-culture conditions, leading to large-scale rice-based co-culture extracts analyzed by HPLC and ^1^H NMR. This led to the discovery of thirteen metabolites from co-culture extracts, including two new polyketides named phaseolorin K (**1**) and pestaphthalide C (**7**), as well as eleven known compounds: phaseolorin J (**2**) [28], phaseolorin D (**3**) [33], dicerandrol C (**4**) [34], dicerandrol A (**5**) [34], 12-*O*-deacetyl-phomoxanthone A (**6**) [35], 3-(2,6-dihydroxyphenyl)-4-hydroxy-6-methylisobenzofuran-1(3H)-one (**8**) [36], nectriapyrone (**9**) [37], wermopyrone (**10**) [38], (R)-mevalonolactone (**11**) [39], 4-Oxo-4H-pyran-3-acetic acid (γ-pyrone-3-acetic acid) (**12**) [40], and (4*RS*)-4,8-dihydroxy-3,4-dihydronaphthalen-1(2H)-one (**13**) [41] (Figure 1). Additionally, to evaluate whether DHS-48 antagonizes HHL-101 through overproduction of non-volatile antimicrobials 5 and 6, we examined HHL-101 hyphal morphology following exposure to these DHS-48-derived metabolites. Herein, we report the co-culture morphology and volatile/non-volatile metabolites interactions, followed by the isolation and structure characterization of compounds (**1**–**13**) and their cytotoxic/immunosuppressive properties.

## 2. Results and Discussions

### 2.1. Morphology of Co-Culture Systems

**Figure 2:**
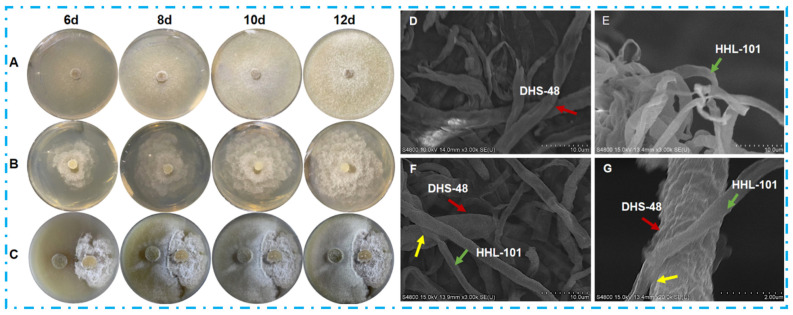
Morphology comparison of *Phomopsis asparagi* DHS-48 and *Pestalotiopsis* sp. HHL-101 in mono- versus co-culture on PDA medium. Colony morphology of (**A**) DHS-48, (**B**) HHL-101, (**C**) DHS-48, and HHL-101 in co-culture. Scanning electron micrographs of (**D**) DHS-48, (**E**) HHL-101, (**F**) DHS-48, and HHL-101 in co-culture (**F**) under ×3000 magnification. (**G**) Magnified view of co-culture interface from (**F**) under ×20,000 magnification. Colored arrows denote the following: Red: DHS-48; Green: HHL-101; Yellow: Interspecies interaction zones.

The fungal phenotype serves as a critical indicator of secondary metabolite biosynthesis potential, with metabolic adaptations often mediating interspecies competition in co-culture systems [14,21]. Physical interactions between DHS-48 and HHL-101 were confirmed via morphology and SEM observation. Figure 2 illustrated the morphological characteristics of DHS-48 and HHL-101 (both mono-cultures and co-cultures) cultured on the PDA medium at 28 °C, with growth observed at 6, 8, 10, and 12 days post-inoculation. For the DHS-48 mono-culture (Figure 2A), initial growth on day 6 exhibited sparse, white, fluffy mycelial expansion; by day 8, colonies developed into dense, velvety structures with yellow-brown undersides. At day 10, radial growth accelerated centrally and spread outward in an annular circle pattern, forming concentric growth rings, and achieved complete medium coverage (90 mm diameter) at day 12. While in the HHL-101 mono-culture (Figure 2B), hyphae displayed cotton-like white texture with regular radial expansion at day 6, reaching 90 mm diameter at day 12. Co-culture systems, as observed in Figure 2C, revealed competitive interactions altering growth trajectories, resulting in temporally divergent morphological development compared to respective mono-culture counterparts. At day 6, the mycelium of HHL-101 appeared dense and robust with distinct growth areas, whereas DHS-48 appeared sparse compared to its mono-culture, and a pronounced mycelia-free zone was observed between these two fungi strains; this stark contrast suggested that HHL-101 restricted the growth of DHS-48 and held a competitive advantage during the initial co-culture phase. When DHS-48 was co-cultured with HHL-101 on days 8 to 12, contacts between both mycelia were observed at certain spots on the pigmented interfacial zone formed at the mycelial confrontation front, accompanied by altered mycelial morphology. Notably, DHS-48 exhibited accelerated hyphal growth concurrent with a dark-yellow medium discoloration, correlating with elevated yellow secondary metabolites secretion. These adaptive responses align with fungal defense mechanisms documented in competitive coexistence systems, where secondary metabolite diversification enhances survival under competitive stress [11], indicating that DHS-48 employed chemically mediated resistance as a defense to mitigate HHL-101-induced suppression to boost survival. The reciprocal metabolic suppression and adaptive countermeasures observed in co-cultured DHS-48 and HHL-101 highlight an evolutionary conserved defense paradigm analogous to fungal competition strategies, potentially driving the biosynthesis of novel bioactive metabolites that remain underrepresented in existing natural product repositories.

Scanning electron microscopy (SEM) was adopted to examine the morphology of the species involved in the interactions between DHS-48 and HHL-101 during co-cultivation versus mono-culture at day 12 under magnifications of ×3000 (Figure 2D–F) and ×20,000 (2G). Monocultured DHS-48 exhibited thicker mycelial networks with a slightly wrinkled surface compared to HHL-101, whose hyphae displayed a more uniform texture (Figure 2D,E). Co-culture systems (Figure 2F,G), however, induced significant morphological remodeling; both species formed densely interwoven hyphal mats characterized by intricate interlocking structures and smoother cell surfaces. This phenotypic shift in co-culture likely reflects adaptive responses to interspecies competition and adaptive responses, as previously documented in fungal stress-tolerance mechanisms, through cell surface modulation in unfavorable biotic circumstances [42]. Collectively, these alterations coincided with enhanced metabolite biosynthesis, underscoring the pivotal role of interspecific interactions in driving ecological specialization and secondary metabolite production.

### 2.2. Chemical Interaction Mediated by VOCs in the Co-Culture Systems

**Figure 3:**
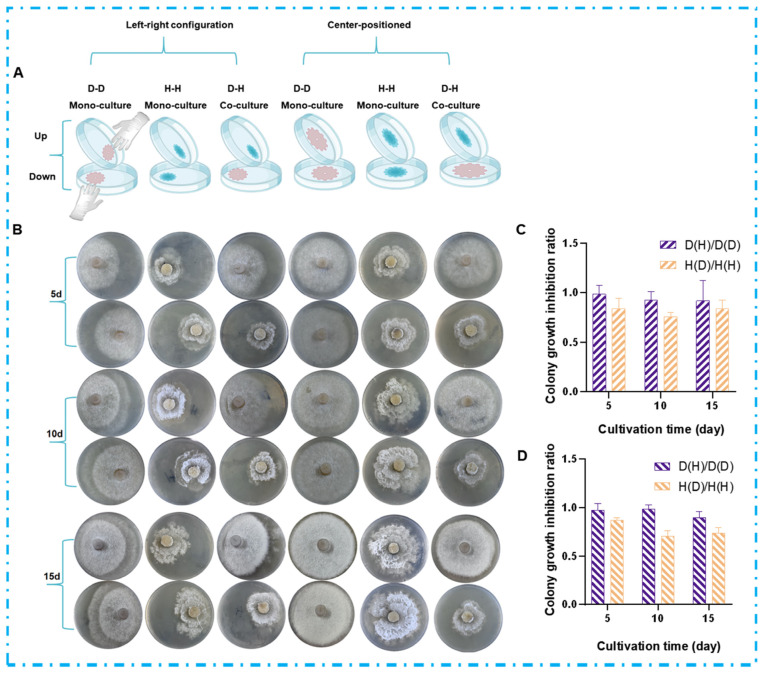
Effect of VOCs on colony morphology of DHS-48 (abbreviated as “D”) and HHL-101 (abbreviated as “H”); D-D denotes the mono-culture of strain DHS-48, H-H represents the mono-culture of strain HHL-101, and D-H indicates the co-culture of both DHS-48 and HHL-101. The ratio D(H)/D(D) corresponds to the average colony diameter of DHS-48 under co-culture conditions relative to that under mono-culture conditions. Similarly, the ratio H(D)/H(H) represents the average colony diameter of HHL-101 in co-culture compared to that in mono-culture. These ratios serve as indicators for evaluating the mutual inhibitory effects between the two strains when cultured together. (**A**) Experimental setup schematic for VOC exposure assay. (**B**) Representative colony morphologies at 5, 10, and 15 days. Colony growth inhibition ratio under VOC exposure (**C**) agar blocks: left-right configuration, (**D**) agar blocks: center-positioned.

Volatile organic compounds (VOCs), as a class of chemically diverse metabolites capable of aerial dissemination, play a significant role in indirect chemical and ecological interactions among microorganisms. To investigate VOC-mediated chemical interactions during fungal co-culture while avoiding physical contact and the exchange of non-volatile metabolites, this study designed a partitioned co-culture system. Colonies (1 cm^2^) of both strains were inoculated on opposite sides of a divided 90 mm Petri dish (using both left-right and center-positioned configurations), and the assembly was sealed to create a shared headspace (Figure 3A). As shown in Figure 3B, both strains exhibited noticeable morphological changes in the co-culture system compared to axenic cultures. In the DHS-48 and HHL-101 co-culture system, the colony diameter of DHS-48 was significantly smaller than that of its mono-culture, while the colony diameter of HHL-101 was also markedly reduced compared to its solitary culture. To further quantify the VOC-mediated interactions, the average colony diameter was systematically measured using the cross-streak method (data provided in Tables S1 and S2), and the growth inhibition rate was calculated accordingly (results shown in Table S3 and Figure 3C,D). Under VOCs exposure, the average colony diameter of DHS-48 or HHL-101 in the co-culture system was smaller compared to its mono-culture at days 5, 10, and 15. Notably, the growth inhibition observed in HHL-101 was more pronounced than that in DHS-48, which was in consistent with the results presented in Figure 2C,D. These findings suggested that DHS-48 exerted greater antagonism over HHL-101 in the later stages of co-culture (8–12 days), thereby maintaining its ecological fitness within the system. Collectively, these results demonstrated a bidirectional growth inhibition between DHS-48 and HHL-101 that was mediated solely by VOCs, independent of hyphal physical contact or non-volatile metabolite exchange.

### 2.3. Volatile Metabolomics Analysis of the Co-Culture Systems

**Figure 4:**
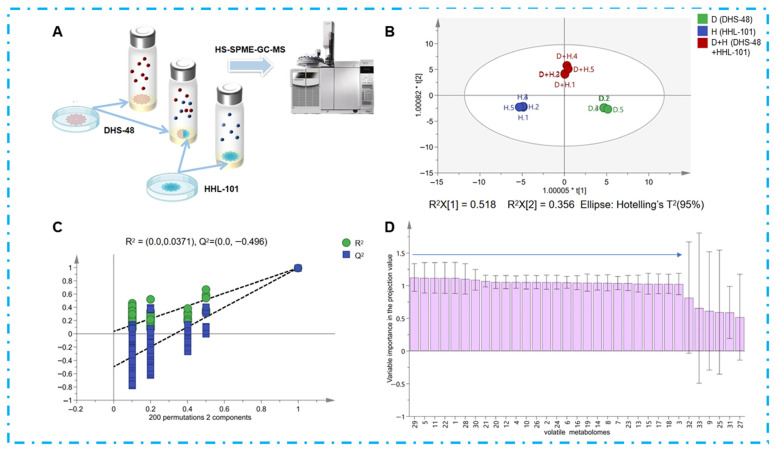
(**A**) DHS-48 and HHL-101 (mono-culture and co-culture) were cultivated directly in SPME bottles, and the volatile fraction was analyzed directly using headspace GC-MS; (**B**) the OPLS-DA score plot for volatiles of DHS-48 and HHL-101 for mono-culture and co-cultures (green spheres represent DHS-48 cultured alone, blue spheres represent HHL-101 cultured alone, red spheres represent DHS-48 co-cultured with HHL-101); (**C**) permutation test plot; (**D**) VIP value chart of volatile components in volatiles for mono-culture and co-culture of DHS-48 and HHL-101 (VIP values are provided in Table S5).

To substantiate VOCs as mediators of chemical interactions between DHS-48 and HHL-101, we comparatively profiled their volatile metabolomes in mono- versus co-culture systems via HS-SPME-GC-MS (Figure 4A). The analysis revealed distinct volatile profiles (Table S4): mono-cultured DHS-48 exhibited 22 VOCs, while HHL-101 mono-culture yielded 8 VOCs. Notably, the co-culture of both fungi generated 22 VOCs, suggesting synergistic or competitive interactions influencing volatile biosynthesis.

To elucidate the impact of VOCs on co-cultivation, multivariate statistical analysis (e.g., orthogonal partial least squares-discriminant analysis, OPLS-DA) was performed on the HS-SPME-GC-MS datasets, identifying key differential compounds to provide insights into the metabolic crosstalk between DHS-48 and HHL-101 in co-culture systems. OPLS-DA, an advanced supervised multivariate statistical method derived from PCA, enables discriminant modeling by orthogonally partitioning covariance into predictive variation and orthogonal uncorrelated variation [43]. As visualized in Figure 4B, the OPLS-DA score plot demonstrated clear separation of volatile profiles across culture conditions. The model (Figure 4C) exhibited robust predictive capacity, with cumulative fit parameters exceeding thresholds for biological relevance (R^2^X = 0.884, R^2^Y = 0.992, Q^2^ = 0.921; all >0.5), confirming robust discriminatory power. Model validation through 200 permutations further demonstrated its reliability with R^2^ = 0.00371 and Q^2^ = −0.496, with the Q^2^ regression line intersecting the negative *y*-axis [Q^2^ (= −0.496) < 0.05], thereby satisfying the criteria for non-overfitting and validating the stability of the discriminant model. These results highlight the utility of OPLS-DA in elucidating cultivar- and interaction-dependent VOCs biosynthesis pathways in microbial consortia. The Variable Importance in Projection (VIP) value, derived from OPLS-DA, quantifies the contribution of individual volatile compounds to class discrimination, with VIP > 1 serving as a threshold for identifying differential VOCs. VIP scores for volatiles (Figure 4D, Table S5) revealed 27 volatiles with VIP > 1, indicating their critical roles in distinguishing metabolic profiles. Co-cultivation triggered metabolic restructuring characterized by the emergence of five newly induced volatiles (e.g., compounds **V1**, **V5**, **V11**, **V22**, and **V29**), including antimicrobial compounds **V11** and **V29** [44]; the disappearance of volatile **V30**; quantitative increases in antimicrobial volatiles **V6** and **V28** [45,46]; and strain-specific modulation, where DHS-48-associated volatiles exhibited disappearance (**V2**, **V4**, **V10**, **V12**, **V20**, **V24** and **V26**), decrease (**V3**, **V7**, **V13**, **V14**, **V17**, **V18**), or increase (**V6**, **V21**), amongst which **V6** demonstrated potential antimicrobial properties [46], while VOCs associated with HHL-101 showed disappearance (**V16**, **V19**) or increase (**V15**, potentially antimicrobial) [47]. These shifts demonstrate complex volatile-mediated chemical communication, indicating that fungi synthesize antifungal agents as defensive mechanisms with potential synergistic effects. Critically, co-culture stimulates secondary metabolite biosynthesis through both direct mycelial contact and volatile signaling pathways. These shifts demonstrate complex volatile-mediated chemical communication, indicating that fungi synthesize antifungal agents as defensive mechanisms with potential synergistic effects. Critically, co-culture stimulates secondary metabolite biosynthesis through both direct physical mycelial contact and volatile signaling pathways.

### 2.4. Non-Volatile Metabolomics Analysis of Small Scale Co-Cultures

**Figure 5:**
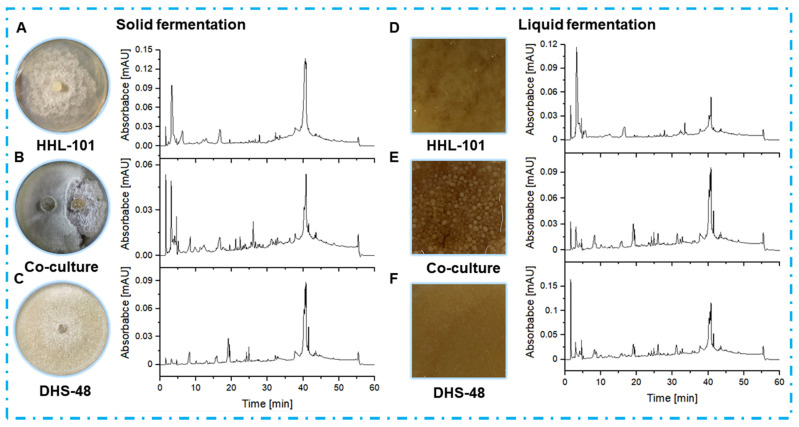
HPLC profiles of EtOAc extracts from DHS-48 and HHL-101 mono-cultures and co-cultures under solid (**A**–**C**) and liquid (**D**–**F**) fermentations. HPLC chromatograms: C18 column (Waters, 5 μm, 10 × 150 mm). Solvents: A, H_2_O; B, MeOH. Linear gradient: 0–5 min, 25% B; 5–15 min, 25–30% B; 15–30 min, 30–55% B; 30–40 min, 55–75% B; 40–50 min, 75–90% B; 50–60 min, 90–100% B. Temperature: 25 °C. Flowrate: 0.8 mL/min. UV detection at λ = 210 nm. Samples were normalized to 1.0 mg/mL (5 µL injection).

To provide evidence that non-volatile metabolites mediate the chemical interaction between DHS-48 and HHL-101, comparative analyses of mono- and co-culture systems under solid-fermentation and liquid fermentation were conducted, focusing on mycelial morphology, broth rheology, and secondary metabolite profiles (Figure 5).

In solid-state fermentation (Figure 5A–C), co-cultured extracts exhibited higher peak intensities at retention times of 10–35 min (putative pyrones and chromones) and 30–45 min (dimeric xanthones) compared to mono-cultures, suggesting synergistic activation of silent biosynthetic gene clusters. Notably, the co-culture system produced three novel peaks (RT: 28.3, 32.1, and 36.5 min) absent in mono-cultures. The observed metabolic shifts align with previous reports on fungal co-culture systems, where spatial heterogeneity in solid matrices promotes interspecies communication and secondary metabolite biosynthesis [48]. Liquid fermentation co-cultures (Figure 5D–F) revealed that mono-cultured mycelia exhibited uniform dispersion without aggregation, whereas co-cultured mycelia formed smooth spherical structures. This morphological shift correlated with a reported reduction in broth viscosity, enhancing mass transfer and promoting vigorous growth to elevate metabolite synthesis efficiency [49]. Despite these kinetic improvements, liquid fermentation co-cultures showed only 1.2–1.5-fold increases in peak intensities, with no novel chromatographic features, indicating limited metabolic pathway crosstalk under submerged conditions. These findings highlight the superiority of solid-state fermentation for co-culture applications and was selected for subsequent large-scale co-culture, in which both biomass accumulation and metabolite diversity synergistically enhanced.

### 2.5. Non-Volatile Metabolomics Analysis of Large-Scale Co-Cultures

**Figure 6:**
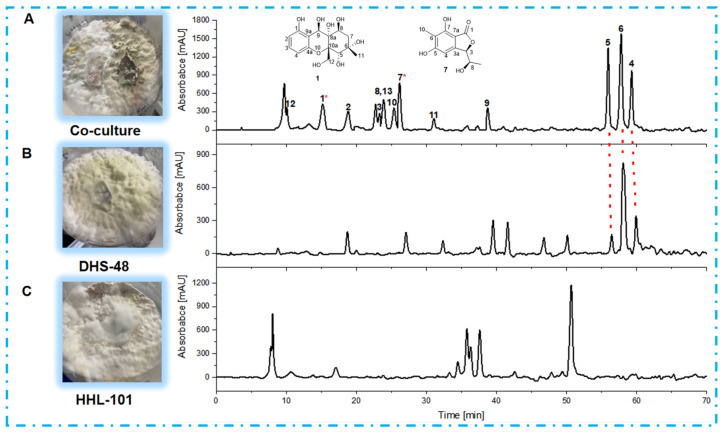
HPLC chromatograms of EtOAc extracts derived from (**A**) DHS-48 and HHL-101 co-culture; and the mono-cultures of (**B**) DHS-48 and (**C**) HHL-101. * Compounds **1** and **7** in (**A**) represent the new compounds induced during co-culture. HPLC chromatograms: C18 column (Agilent Technologies 10 mm × 250 mm). Solvents: A, H_2_O; B, MeOH. Linear gradient: 0 min, 10% B; 70 min, 80% B. Temperature: 25 °C. Flow rate: 2 mL/min. UV detection at λ = 210 nm.

**Figure 7:**
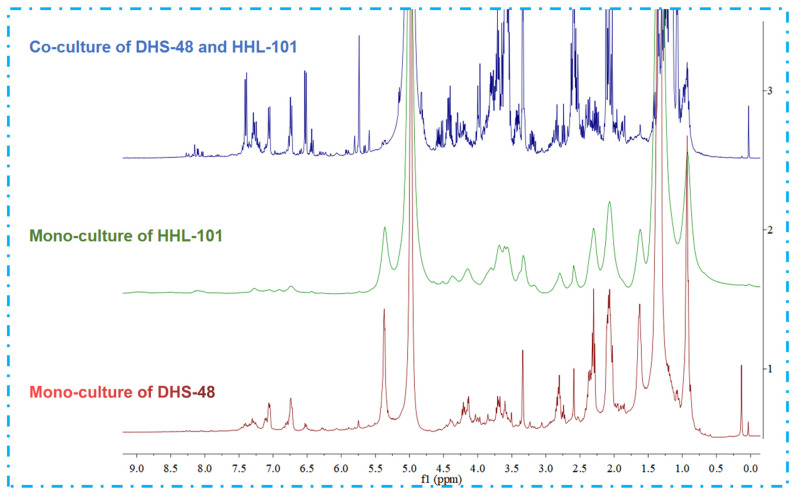
^1^H NMR spectra (400 MHz, CD_3_OD) of EtOAc extracts of DHS-48 and HHL-101 under mono- and co-cultures, chemical shifts (δ) presented in ppm.

Following scale-up fermentation via the selected solid-state approach, HPLC-UV analysis of the EtOAc extract from the 30-day DHS-48/HHL-101 co-culture on rice medium revealed substantial metabolic reprogramming, notably characterized by the emergence of newly induced compounds **1**–**3** and **7**–**13** (Figure 6A), which were absent in the corresponding mono-cultures (Figure 6B,C). Furthermore, the production of the identified metabolites **4**–**6** was markedly increased relative to their respective mono-cultures (Figure 6A,B). Interestingly, compounds **4**–**6** have been reported in the literature to possess significant antimicrobial activity [35,50,51]. These differences were also supported by the fact that the ^1^H NMR metabolic profile (Figure 7) of EtOAc extracts showed several significant hydrogen resonations between 6.3 and 7.7 ppm compared with the mono-cultures.

### 2.6. Structure Elucidation of New Compounds

**Figure 8:**
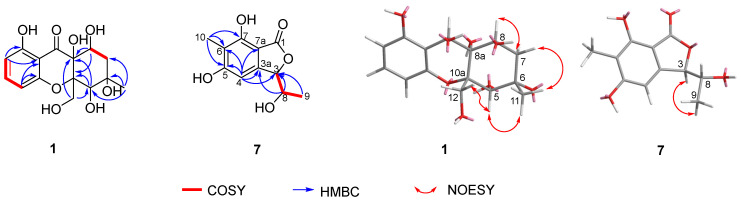
Key COSY, HMBC, and NOESY correlations of compounds **1** and **7**.

**Figure 9:**
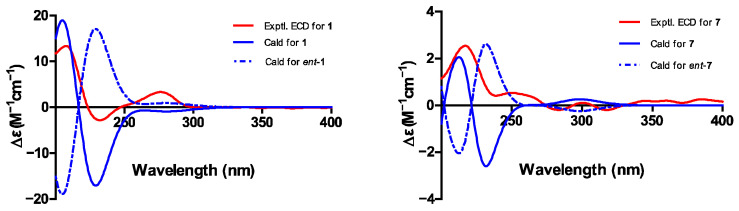
Experimental and calculated electronic circular dichroism (ECD) spectra of **1** and **7**.

Phaseolorin K (**1**) was obtained as light yellow amorphous powder, and its molecular formula was established as C_15_H_20_O_8_ according to its Na^+^-liganded molecular ion at *m*/*z* 351.1058 (calculated for C_15_H_20_O_8_Na, 351.1056) in its high resolution electrospray ionization mass spectrometry (HR-ESI-MS), requiring six unsaturation degrees. The ^1^H and ^13^C NMR data of **1** (Table 1) and its ^1^H-^1^H COSY and HSQC spectra exhibited a series of characteristic resonances for a 1,2,3-trisubstituted benzene ring [*δ*_H_ 6.37 (d, *J* = 8.2 Hz), *δ*_C_ 109.1, d, CH-2; *δ*_H_ 6.97 (t, *J* = 8.2 Hz), *δ*_C_ 129.9, d, CH-3; *δ*_H_ 6.40 (d, *J* = 8.2 Hz), *δ*_C_ 108.8, d, CH-4], three oxygenated methine (*δ*_H_ 4.21, 1H, s, *δ*_C_ 76.8, d, CH-5; 3.90, 1H, d, *J* = 4.9 Hz, *δ*_C_ 70.5, d, CH-8; *δ*_H_ 5.67, 1H, s, *δ*_C_ 64.7, d, CH-9), an oxygenated methylene [*δ*_H_ 4.07, (d, *J* = 7.7 Hz), *δ*_H_ 3.81 (d, *J* = 7.7 Hz); *δ*_C_ 69.3, t, CH_2_-12], one unhybridized methylene [*δ*_H_ 2.15, (dd, *J* = 15.0, 5.4 Hz), *δ*_H_ 1.92 (d, *J* = 15.0 Hz); *δ*_C_ 42.9, t, CH_2_-7], and a tertiary methyl (*δ*_H_ 1.30, 3H, s; *δ*_C_ 20.5, q, CH_3_-11). Comparison of the ^1^H and ^13^C NMR data of **1** with those of phaseolorin D (**3**) co-isolated in the culture medium revealed that both had the same chromone core, except for the absence of a carbonyl group at C-9 and the presence of additional hydroxyl groups attached at C-6 and C-9. Supporting evidence for this assignment was obtained from the upfield chemical shift of C-9 (*δ*_C_ 64.7, CH) and the downfield chemical shift of C-6 (*δ*_C_ 85.4, C) in **1** (*δ*_H_ 2.20, m for H-6 and *δ*_C_ 29.2, CH for C-6; 196.2 for *δ*_C_ C-9 in **2**, respectively) and the critical HMBC correlations from H_3_-11 to C-5, C-6, and C-7 as well as from H-9 to C-1, C-8, C-4a, C-8a, and C-9a (Figure 8). The relative configuration of compound **1** was established by the interpretation of NOE correlations. The NOESY spectrum of **1** showed a correlation of H-8/H-9, H-9/H_2_-12, H_2_-12/H-5, and H-5/H_3_-11, indicating that these protons are on the same spatial orientation. The absolute configuration of **1** was theoretically deduced to be the same as that of **3** from the biogenetic consideration and confirmed with the aid of the calculated CD spectrum method, which expectedly launched a calculated CD spectrum of the truncated model 5*S*, 6*S*, 8*S*, 8a*R*, 9*S*, 10a*S*-**1** perfectly matched with the experimental one (Figure 9). The configuration of **1** was conclusively assigned by the combined analysis of NOESY correlations and ECD calculations. The compound is therefore designated as phaseolorin K.

Pestaphthalide C (**7**), isolated as a colorless oil, had a molecular formula of C_11_H_12_O_5_ with six degrees of unsaturation, as determined by HRESIMS (*m*/*z* 223.0614 [M-H]^−^, calc. for C_11_H_11_O_5_ 223.0612). The 1D NMR data of **7** (Table 1) and its ^1^H-^1^H COSY spectrum (Figure 8) exhibited an oxygenated methine [*δ*_H_ 5.24 (d, *J* = 3.7 Hz), *δ*_C_ 83.0, d, CH-3], an aromatic proton (*δ*_H_ 6.51, s, *δ*_C_ 102.1, d, CH-4), a methyl group (*δ*_H_ 2.05, s, *δ*_C_ 8.5, q, 10-CH_3_), and a 1-hydroxyethyl group (CH-8 to CH_3_-9). The HMBC spectrum showed correlations of H-4/C-3, C-5 (*δ*_C_ 163.9), C-6 (*δ*_C_ 112.8), C-7a (*δ*_C_ 105.0), and C-8 (*δ*_C_ 68.8), which permitted construction of the 5,6,7-trisubstituted isobenzofuranone ring. Furthermore, key HMBC correlations of H_3_-10/C-5 (*δ*_C_ 163.9), C-6 (*δ*_C_ 112.8), and C-9 (*δ*_C_ 138.3); H-8 [*δ*_H_ 4.10 (qd, *J* = 6.5, 3.7 Hz)]/C-3 and C-3a (*δ*_C_ 151.3); and H_3_-9/C-3 allowed for the connection of CH_3_-10 and the 1-hydroxyethyl group at C-6 and C-3 positions, respectively. The proposed planar structure is identical to pestaphthalides A and B [52] isolated from plant endophytic fungus *Pestalotiopsis foedan*. The NOESY spectrum (Figure 8) disclosed the correlations between H-3/H-8 and enabled the assignment of the same orientation of H-3 ad H-8 as pestaphthalide A. Interestingly, the measured optical rotation value ${\left[\alpha \right]}_{D}^{20}$ + 60 (*c* 0.0001, MeOH) of **7** reported in this work does not correspond to those reported for pestaphthalide A ${\left[\alpha \right]}_{D}$ + 51 (*c* 0.05, MeOH), and the chemical shift of CH-3 [*δ*_H_ 5.32 (d, *J* = 2.4 Hz), *δ*_C_ 85.4, d, CH-3], CH-4 (*δ*_H_ 6.67, s, *δ*_C_ 102.0, d) and CH-8 [*δ*_H_ 4.19 (qd, *J* = 6.3, 2.4 Hz), *δ*_C_ 68.8, d] in pestaphthalide A changed to the current observed values in **7**, indicating that **7** was a diastereoisomer of pestaphthalide A. As shown, the 3*R*, 8*R* configuration was defined based on the calculated ECD spectrum in good accordance with the experimental curve (Figure 9).

### 2.7. Chemical Interaction Mediated by Non-Volatile Antimicrobial Metabolites

**Figure 10:**
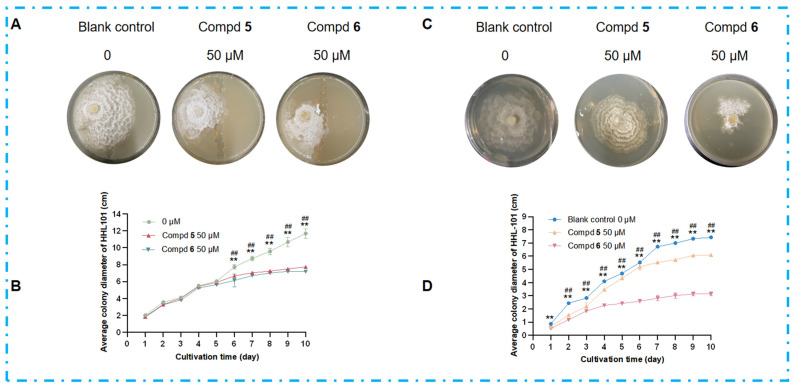
Effects of antimicrobial metabolites **5** and **6** on HHL-101 growth inhibition and morphological alterations. (**A**) Mycelial morphology and (**B**) average colony diameter dynamics of HHL-101 after 10 days on 14 cm PDA plates, with **5**/**6** (0, 50 μM) restricted to the central 1 cm region. (**C**) Mycelial morphology and (**D**) average colony diameter dynamics of HHL-101 in 9 cm PDA plates treated with compounds **5**/**6** (0, 50 μM). # *p* < 0.05, ## *p* < 0.01 blank control group compared to compd **5** group; * *p* < 0.05, ** *p* < 0.01 blank control group compared to compd **6** group.

Microbes can produce compounds that function as transcriptional regulators and epigenetic modifiers, as evidenced by their roles in regulating stress-responsive gene networks and secondary metabolite biosynthesis remodeling [53]. To investigate whether the antimicrobial metabolites produced by DHS-48 during co-culture could act as chemical agents to inhibit HHL-101 and thereby enhance its competitive resource acquisition, we selected compounds **5** and **6** (high-yield secondary metabolites of DHS-48) for experimental validation (Figure 6). Spatial inhibition assays revealed that compounds **5** and **6**, when positioned as a midline barrier, completely blocked HHL-101 penetration (Figure 10A), indicating that DHS-48 established chemically defined exclusion zones in the co-culture system. Comparable levels of growth inhibition were demonstrated by the colony diameters of HHL-101 in co-culture with DHS-48 or when exposed to compound **5** or **6** alone (Figure 10B, Table S6), suggesting that the inhibitory effect of DHS-48 during co-culture is primarily attributable to the activity of these metabolites. Morphological disruption was further evidenced by distorted hyphal structures in HHL-101 upon exposure to compounds **5** and **6** in the culture medium (Figure 10C), characterized by desiccation, curling, and marginal yellowing. Quantitative analysis confirmed significant reduction in the HHL-101 growth rate (Figure 10D, Table S7), establishing the observed significantly reduced colony diameter, confirming their potent growth-inhibitory effects. These findings collectively demonstrate that DHS-48 employs upregulated antimicrobial metabolites, for instance, antimicrobial metabolites **5** and **6,** as chemical weapons to suppress competitors, thereby securing ecological dominance through resource monopolization.

### 2.8. Biological Evaluation of Isolated Compounds

All isolated metabolites were assessed for their cytotoxic and immunosuppressive properties. Amongst these, compounds **4**–**6** exhibited significant cytotoxicity towards human liver cells HepG-2 (IC_50_ values of 14.03 ± 0.56, 4.94 ± 0.33, and 13.11 ± 0.43) and cervical cancer cells Hela (IC_50_ values of 24.17 ± 2.35, 19.26 ± 0.78, and 21.06 ± 0.87), compared to the positive controls adriamycin and fluorouracil (Table 2). The findings indicated that the enhanced production of compounds **4**–**6** by DHS-48 during co-culture may confer a competitive advantage against HHL-101 through inhibitory mechanisms. Compound **2** showed marginal immunosuppressive activity against the proliferation of ConA-induced (T-cells) and LPS-induced (B-Cells) in murine splenic lymphocytes (Table 3).

## 3. Materials and Methods

### 3.1. General Procedures

The ATR-W2 HHW5 digital Abbe refractometer (Shanghai Physico-optical Instrument Factory, Shanghai, China) was employed to acquire the optical rotations. The Shimadzu UV-2600 PC spectrophotometer (Shimadzu Corporation, Tokyo, Japan) was employed to acquire the UV spectra, and the JASCO J-715 spectra polarimeter (Japan Spectroscopic, Tokyo, Japan) was employed to measure the ECD spectra. The ^1^H, ^13^C, and 2D NMR spectra were obtained using a Bruker AV 400 NMR spectrometer using TMS as the internal standard. Column chromatography (CC) was conducted on a Sephadex-LH-20 (18–110 µm, Merck, Darmstadt, Germany) or silica gel (200–400 mesh, Qingdao Marine Chemical Inc., Qingdao, China). The HPLC analysis of liquid and solid fermentation in small quantities was conducted on a C18 column (Waters, 5 μm, 10 × 150 mm) using a Waters e2695 (Waters Corporation, Milford, MA, USA). Semi-preparative HPLC was achieved using a C18 column (Agilent Technologies 10 mm × 250 mm) in an Agilent Technologies 1200 LC (Santa Clara, CA, USA). The purity of the isolated compounds was ascertained using high-performance liquid chromatography (HPLC) on an Agilent 1200 instrument and a reverse-phase column (4.6 × 150 mm, 5 μm). The detection wavelength was 210 nm in ultraviolet. Scanning electron micrographs (SEM) analysis was obtained using a Hitachi S-4800 (Hitachi, Ltd., Tokyo, Japan). HS-SPME-GC-MS was obtained using an Agilent Technologies 7890B/7000B.

### 3.2. Fungal Material

The endophytic fungus *Phomopsis asparagi* DHS-48 was isolated from the fresh roots of the mangrove plant *Rhizophora mangle*, collected in October 2015. The endophytic fungus *Pestalotiopsis* sp. was isolated from a fresh, healthy branch of *Rhizophora stylosa* (Rhizophoraceae) harvested in September 2014. Both originated from Dong Zhai Gang-Mangrove Garden on Hainan Island, China. The fungi were classified as *Phomopsis asparagi* (strain no. DHS-48) and *Pestalotiopsis* sp. (strain no. HHL-101) based on ITS gene sequencing (GenBank Accession No. MT126606 and No. EF451799) [54]. Two voucher strains were deposited in the laboratory of one of the authors (J.X.).

### 3.3. Preparation of Phomopsis asparagi DHS-48, Pestalotiopsis sp. HHL-101, Co-Cultivation, and Morphological Observation

The *Phomopsis asparagi* DHS-48 and *Pestalotiopsis* sp. HHL-101 were individually cultivated on PDA for 7 days at 28 °C. For co-cultivation, two 1 cm^2^ agar segments from each fungus were positioned 4.5 cm apart on a fresh agar plate with a diameter of 9 cm. The co-cultures were incubated at 28 °C for a duration of 12 days. Simultaneously, mono-cultures of each strain designated for co-cultivation were established and grown under identical circumstances for comparative analysis. After 12 days, the agar blocks containing mycelium of DHS-48, HHL-101, and their co-culture were excised and combined with a 2.5% glutaraldehyde solution at 4 °C for 4 h, followed by thorough rinsing in phosphate-buffered saline (PBS) three times for 15 min each. The specimens underwent dehydration thrice in a graded ethanol series (50%, 70%, 80%, 90%, and 100%) for 30 min each, followed by lyophilization, fixation on a substrate with conductive gel, gold sputtering for 30 s using an IB-3 ion coater, and, ultimately, examination with a Hitachi S-4800 scanning electron microscope.

### 3.4. Effect of Volatiles on Colony Morphology of Phomopsis asparagi DHS-48, Pestalotiopsis sp. HHL-101

To prevent contact, fungal samples of *Phomopsis asparagi* DHS-48 and *Pestalotiopsis* sp. HHL-101, each measuring 1 cm^2^, were injected on two 9 cm diameter plates (without lids), which were then sealed together. Two inoculation methods were selected (agar blocks positioned on the left-right relative configuration of the plate or in the center of the plate facing each other), with each method comprising three distinct combinations. The DHS-48-DHS-48 (D-D) were positioned in a left-right relative orientation; the HHL-101-HHL-101 (H-H) were arranged in a left-right relative orientation; the DHS-48-HHL-101 (D-H) were set in a left-right relative orientation; the DHS-48-DHS-48 (D-D) were positioned in the center of the plate facing each other; the HHL-101-HHL-101 (H-H) were situated in the center of the plate facing; the DHS-48-HHL-101 (D-H) were placed in the center of the plate facing each other. The plates were inverted and incubated in a thermostat at 28 °C for 5, 10, and 15 days to assess colony color, size, and mycelial growth, with images captured for archival purposes. The aforementioned trials were conducted thrice concurrently.

### 3.5. Experimental Conditions for DHS-48 and HHL-101 Mono- and Co-Cultures Analyzed Using HS-SPME-GC-MS

First, SPME bottles were placed into 2 mL of PDA. Mono-cultures of DHS-48 and HHL-101 were established by inoculating 2 mm single colonies of DHS-48 or HHL-101, respectively, into Petri dishes containing Potato Dextrose Agar (PDA). DHS-48 and HHL-101 co-cultures were established by placing two 2 mm DHS-48 and HHL-101 single colonies on opposite sides of the SPME vessel. After inoculation, each vial was sealed with a suitable SPME cap. Cultures were incubated for 12 days under dark conditions at 28 °C. After 12 days, five replicates (*n* = 5) of each mono-culture and co-culture were assayed using a gas chromatography-mass spectrometer.

### 3.6. HS-SPME-GC-MS for Analyzing Volatile Fractions

The headspace of each individual single culture and co-culture was sampled with a 2 cm DVB/CAR/PDMS SPME fiber (Supelco, Bellefonte, PA, USA) directly in the SPME vial used for cultivation. Sampling was carried out by an MPS2 autosampler at 60 °C (no pre-equilibrium, 30 min of sampling). The fiber was automatically injected in an Agilent 7890B/7000B Gas chromatography triple quadrupole mass spectrometer. Sampling was only performed once for each culture to avoid any perturbation of the volatile fraction over time. The GC-MS conditions were as follows: inlet temperature 250 °C, no split injection (1 min), carrier gas (helium at a flow rate of 3 mL/min); column: HP5MS (30 m × 250 µm× 0.25 µm; Agilent Technologies). Volatile metabolite profiling was carried out with the following temperature program: 50 °C (1 min)–5 °C/min–300 °C (5 min). MS was operated in the EI mode at 70 eV with a mass range of 35–350 amu in full-scan mode.

### 3.7. Processing of GC-MS Data and Deconvolution of GC Peaks

Data analysis was performed using Agilent MassHunter Qualitative Analysis software (version B.07.00). The identification of chromatographic peaks was based on a combination of linear retention indices (calculated against a C9–C25 n-alkane series) and mass spectrometric data. Mass spectra were matched using the NIST17.L library, supplemented by manual verification, with a match factor threshold greater than 80% applied for compound identification.

### 3.8. Small-Scale Fermentation of Liquid and Solid Media for DHS-48 and HHL-101 in Mono-Culture and Co-Culture

First, individual DHS-48 or HHL-101 colonies were inoculated into Potato Dextrose Agar (PDA), which were then incubated and inverted for five days at 28 °C. Then, 500 mL flasks filled with 100 mL of Potato Dextrose Broth (PDB) were inoculated with individual 1 cm^2^ colonies of DHS-48 or HHL-101, which were then continuously shaken in mono-cultures at 28 °C for 10 days. Concurrently, 500 mL flasks containing 100 mL of potato dextrose broth (PDB) were inoculated with 1 cm^2^ single colonies of DHS-48 and HHL-101 for co-cultivation under identical circumstances. A 1 cm^2^ single colony of the single culture DHS-48 or HHL-101 was inoculated onto the center of a fresh agar plate with a diameter of 9 cm for mono-culture (15 mL agar media inverted and incubated at 28 °C for 12 days); subsequently, 1 cm^2^ agar segments from each fungus were placed 4.5 cm apart on a fresh agar plate with a diameter of 9 cm for co-cultures (15 mL agar media inverted and incubated at 28 °C for 12 days). All culture groups were made and measured in triplicate. The cultures were extracted thrice with EtOAc (50 mL × 3 for each PDA plate, 500 mL × 3 for each PDB flask). The EtOAc-soluble substances were eluted with a flow rate of 0.8 mL·min^−1^ over a 60 min gradient (Solvents: A, H_2_O; B, MeOH), as follows: 0–5 min, 25% B; 5–15 min, 25–30% B; 15–30 min, 30–55% B; 30–40 min, 55–75% B; 40–50 min, 70–90% B; and 50–60 min, 90–100% B at 25 °C with UV detection at λ = 210 nm.

### 3.9. Large Fermentation by Solid Fermentation and Isolation of Compounds

Mono-culture of DHS-48 or HHL-101 was initially inoculated onto Petri dishes with potato dextrose agar (PDA) at 28 °C for 5 days. Subsequently, a 1 cm^2^ single colony of both DHS-48 and HHL-101 was inoculated into Erlenmeyer flasks (160 × 1 L), each containing 100 g of rice and 100 mL of 0.3% of saline water and fermented at 28 °C for 30 days. A total of 160 flasks of extracted cultures were filled three times with 400 mL of EtOAc, and the filtrate was evaporated under reduced pressure to yield a crude extract of 90 g. Five fractions (Fr. 1–Fr. 5) were obtained by chromatographing the EtOAc extract on a silica gel column chromatography (CC), utilizing a step gradient elution with CH_2_Cl_2_-MeOH (0–100%). Fr. 2 underwent gradient elution with CH_2_Cl_2_-EtOAc (5:1, *v*/*v*) for open silica gel CC, producing six fractions Fr. 2.1–2.6. Compound **4** (30 mg) was obtained by subjecting Fr. 2.3 to open silica gel CC utilizing gradient elution with CH_2_Cl_2_-EtOAc (4:1, *v*/*v*). To obtain compound **5** (100 mg), Fr. 2.4 was exposed to open silica gel CC utilizing gradient elution with CH_2_Cl_2_-EtOAc (4:1, *v*/*v*). To obtain compound **6** (300 mg), Fr. 2.6 was submitted to open silica gel CC utilizing gradient elution with CH_2_Cl_2_-EtOAc (3:1, *v*/*v*). Fifth fractions (Fr. 4.1–Fr. 4.5) were obtained by silica gel separation of Fr. 4 using CH_2_Cl_2_-EtOAc (1:1, *v*/*v*). Compound **11** (20 mg) was obtained by purifying Fr. 4.1 using Sephadex LH-20 CC using MeOH-CH_2_Cl_2_ (1:1, *v*/*v*). Reversed-phase HPLC (MeOH-H_2_O 60:40, *v*/*v*) was performed on Fr. 4.2 to obtain compounds **9** (2 mg), **10** (2 mg), and **12** (2 mg). Reversed-phase HPLC (MeOH-H_2_O 55:45, *v*/*v*) was performed on Fr. 4.3 to extract compounds **8** (3 mg) and **13** (3 mg). Compound **7** (2 mg) was obtained by purifying Fr. 4.4 using Sephadex LH-20 CC (CH_2_Cl_2_/MeOH, 1:1, *v*/*v*) and then undergoing reversed-phase HPLC (MeOH-H_2_O 55:45, *v*/*v*). Fr. 5 was subjected to open silica gel CC using gradient elution with CH_2_Cl_2_-EtOAc (1:1, *v*/*v*) to yield six fractions (Fr. 5.1–5.6). Compounds **1** (4 mg), **2** (6 mg), and **3** (5 mg) were obtained by purifying Fr. 5.3 using Sephadex LH-20 CC (CH_2_Cl_2_/MeOH, 1:1, *v*/*v*) and then undergoing reversed-phase HPLC (MeOH-H_2_O 40:60, *v*/*v*).

Phaseolorin K (**1**): red amorphous powder (MeOH); [α]^20^_D_ + 70 (c 0.01, MeOH); UV (MeOH) λ_max_ 212 nm; ^1^H and ^13^C NMR data, see Table 1; HRESIMS *m*/*z* 351.1058 [M + Na]^+^ (calc. for C_15_H_20_O_8_Na 351.1050).

Pestaphthalide C (**7**): Colorless oil (MeOH); [α]^20^_D_ + 13 (c 0.01, MeOH); UV (MeOH) λ_max_ 261 nm; ^1^H and ^13^C NMR data, see Table 1; HRESIMS *m*/*z* 223.0614 [M − H]^−^ (calc. for C_11_H_11_O_5_ 223.0612).

### 3.10. Inhibitory Effects of Compounds (Produced in Large Quantities by DHS-48 After Co-Culture) on HHL-101

PDA medium containing final concentrations of 0, 50 µM of compound **5** and compound **6** (DMSO as solvent) were prepared, respectively. In order to observe more clearly the inhibition of HHL-101 growth by the compounds produced in large quantities after DHS-48 co-culture, two methods of administration were used. In the first method, a 1 cm^2^ single colony of HHL-101 was inoculated into a 14 cm diameter PDA plate approximately 3.5 cm from the middle limit on the left side (14 cm diameter PDA plate containing 24 mL agar medium), and after the medium solidified, a 1 cm diameter test tube was used to dig a 1 cm boundary in the middle of the medium. The middle boundary was filled with PDA medium containing a final concentration of 0, 50 µM of compound **5** and compound **6**, respectively. In the second method, a 0.5 cm^2^ single colony of HHL-101 was inoculated into in the middle of 9 cm diameter PDA plates (15 mL agar medium containing a final concentration of 0, 50 µM of compound **5** and compound **6**, respectively). The plates were inverted and incubated at 28 °C in a thermostat for 10 days to observe the colony color, size, and mycelial growth, and photographs were taken and stored. The colony diameter was measured daily. The above experiments were performed in three parallel experiments. The mean values of colony diameters measured in the three parallel experiments were taken and the standard deviation was calculated and analyzed.

### 3.11. Electron Circular Dichroism Calculation

Using the Merck Molecular Force Field (MMFF) and Spartan’s 14 program, specific Monte Carlo conformational searches were conducted. For ECD calculations, conformers with a Boltzmann population greater than 0.4% were selected (Tables S8–S11). Then, using the PCM polarizable conductor calculation model, the conformers were first optimized at the B3LYP/6-31 g level in gas. Time-dependent density functional theory (TD-DFT) was used to theoretically calculate ECD in MeOH for all conformers of compounds **1** and **7** at the B3LYP/6-31+g (d, p) level. Rotatory strengths were computed for 30 excited states in total. Gaussian band shapes with sigma = 0.3 eV were applied to dipole-length rotational strengths to create ECD spectra using the software SpecDis 1.6 (University of Würzburg, Würzburg, Germany) and GraphPad Prism 5 (University of California San Diego, USA).

### 3.12. Cytotoxicity Assay

The HepG2 liver cancer cell line and the HeLa cervical cancer cell line were acquired from the Type Culture Collection of the Chinese Academy of Sciences in Shanghai, China. The cells were cultured utilizing RPMI-1640 media. The cytotoxicity against HepG2 and HeLa cells was evaluated using the 3-(4,5-dimethylthiazol-2-yl)-2,5-diphenyltetrazolium bromide (MTT) assay, obtained from Sigma-Aldrich, St. Louis, MO, USA, as previously outlined [55]. Additionally, adriamycin (sourced from Shanghai Macklin Biochemical Co., Ltd., with a purity of 99.8%) (Shanghai, China) and 5-fluorouracil (5-FU) (obtained from Beijing Solarbio Science and Technology Co., Ltd., with a purity of 99.8%) (Beijing, China) were utilized as positive controls.

### 3.13. Splenocyte Proliferation Assay

Spleen cells were harvested from BALB/c mice (purchased from Hunan Sja Laboratory Animal Co., Ltd, Changsha, China) under sterile conditions, seeded in a 96-well plate at a density of 1 × 10^7^ cells/mL per well, and stimulated with Con A (5 μg/mL) or LPS (10 μg/mL) or cyclosporine A (CsA) at 37 °C and 5% CO_2_ for 48 h. Subsequently, 20 μL of CCK-8 was introduced to each well four hours before to the conclusion of the incubation period. The absorbance at OD_450_ was recorded using an ELISA reader, and the IC_50_ value was derived from the correlation curve relating chemical concentration to OD_450_.

### 3.14. Statistical Analysis

All the cell data are presented as the mean standard deviation of the means (S.D.), and a one-way analysis of variance (ANOVA) was used to evaluate the statistical significance of the differences between the groups using GraphPad Prism 5.01. OPLS⁃DA and VIP values were analyzed using SIMCA-P.

## 4. Conclusions

Fungal co-cultivation has emerged as an effective technique for stimulating dormant biosynthetic pathways and discovering new bioactive chemicals. To effectively highlight induction processes, we acquired a thorough comprehension of intermicrobial interactions at the molecular level concerning both volatile and non-volatile compounds produced during co-culture. Subsequent examination was performed, employing HS-SPME-GC-MS (culminating in multivariate statistical analysis (OPLS-DA)), HPLC chromatogram, and ^1^H NMR spectroscopy. A total of 22 volatiles were detected in DHS-48 under mono-culture conditions, 8 volatiles were discovered from HHL-101 in mono-culture, and 22 volatiles were identified from the co-culture of DHS-48 and HHL-101. The experiments also led to the isolation of two new polyketides, named phaseolorin K (**1**) and pestaphthalide C (**7**), and 11 known compounds in non-volatile metabolites. Compound **2** exhibited considerable inhibitory action against the proliferation of ConA-induced T lymphocytes and LPS-induced B murine spleen lymphocytes, whereas compounds **4**–**6** showed comparable or superior in vitro cytotoxicity against the investigated human cancer cell lines relative to the positive control. Our study elucidates the volatile and non-volatile metabolites produced during co-culture, indicating that co-culture has significant potential for augmenting the generation and/or accumulation of novel chemodiversity from mangrove endophytic fungus.

## Figures and Tables

**Table 1:** ^1^H (400 MHz) and ^13^C (100 MHz) NMR data of **1** and **7** in CD_3_OD.

Position	1		7	
	*δ*_C_ Type	*δ*_H_ (*J* in Hz)	*δ*_C_ Type	*δ*_H_ (*J* in Hz)
1	159.3, C		173.5, C	
2	109.1, CH	6.37, d, 8.0		
3	129.9, CH	6.97, t, 8.2	83.3, CH	5.24, d,3.7
3a			151.3, C	
4	108.8, CH	6.40, d, 8.2	102.1, CH	6.51, s
4a	154.5, C			
5	76.8, CH	4.21, s	163.9, C	
6	85.4, C		112.8, C	
7	42.9, CH_2_	H_a_ 2.15, dd, 15.0, 5.4	158.3, C	
		H_b_ 1.92, d, 15.0		
7a			105.0, C	
8	70.5, CH	3.90, d, 4.9	68.8, CH	4.10, m
8a	74.5, C			
9	64.7, CH	5.67, s	20.4, CH_3_	1.20, d, 6.5
9a	109.9, C			
10			7.84, CH_3_	2.05, s
10a	86.4, C			
11	20.5, CH_3_	1.30, s		
12	69.3, CH_2_	H_a_ 4.07, d, 7.7		
		H_b_ 3.81, d, 7.7		

**Table 2:** Cytotoxicity of compounds **1**–**13**.

Compound	IC_50_ (µM) ^a^	
	HepG2	Hela
**1**–**3**	\	\
**4**	14.03 ± 0.56	24.17 ± 2.35
**5**	4.94 ± 0.33	19.26 ± 0.78
**6**	13.11 ± 0.43	21.06 ± 0.87
**7**–**13**	\	\
Adriamycin ^b^	\	0.92 ± 0.56
Fluorouracil ^c^	178.12 ± 28.82	\

^a^ Data are presented as mean ± SD from three separate experiments. ^b^ Hela cell positive control. ^c^ Hepg2 cell positive control. ‘\’ stands for no inhibitory effect at 200 µM.

**Table 3:** Immunosuppressive activity of compounds **1**–**13**.

Compound	IC_50_ (µM) ^a^	
	ConA-Induced T-Cell Proliferation	LPS-Induced B-Cell Proliferation
**1**	\	\
**2**	42.35 ± 2.49	88.19 ± 2.59
**3**–**13**	\	\
cyclosporin A ^b^	4.39 ± 0.02	25.11 ± 0.43

^a^ Data are presented as mean ± SD from three separate experiments. ^b^ Positive control. ‘\’ stands for no inhibitory effect at 200 µM.

## Data Availability

The original data presented in the study are included in the article/Supplementary Materials; further inquiries can be directed to the corresponding author.

## Supplementary Materials

The following supporting information can be downloaded at https://www.mdpi.com/article/10.3390/md23120452/s1, Figures S1–S50 (complete spectroscopic characterization of compounds **1**–**13**, including ^1^H/^13^C/2D NMR and HR-ESI-MS spectra, along with HPLC purity profiles) and Figure S51, the loadings plot; Tables S1–S7 (fungal growth measurements and VOC analysis data); and Tables S8–S11 (computational chemistry data for compounds **1** and **7**).
