# Supplementary material for: “Hyphae Intertwined, Biomolecules Co-Born”—New Polyketides Induction by Co-Culture of the Mangrove Endophytic Fungus Phomopsis asparagi DHS-48 and Pestalotiopsis sp. HHL-101 at Both Volatile and Non-Volatile Levels
Source: Mar Drugs. 2025 Nov 26;23(12):452. doi: 10.3390/md23120452 (PMC12734679; doi:10.3390/md23120452)
Supplement: Supplementary file 1 [file marinedrugs-23-00452-s001.zip › marinedrugs-3952535-supplementary.pdf]

“Hyphae intertwined, Biomolecules co-born”——New Polyketides Induction by Co-culture of the Mangrove Endophytic Fungus *Phomopsis asparagi* DHS-48 and *Pestalotiopsis* sp. HHL-101 at Both Volatile and Non-volatile Levels

Ting Feng, XiaoJing Li, ZhenYi Liang and Jing Xu *

Collaborative Innovation Center of Ecological Civilization, School of Chemistry and Chemical Engineering, Hainan University, Haikou 570228, China

* Corresponding author.

E-mail addresses: happyjing3@hainanu.edu.cn; Tel.: +86-898-6627-9226; Fax: +86-898-6627-9010

Contents

Figure S1. ^{1}H-NMR (600 MHz, CD_3OD) of compound (**1**) -- 4

Figure S2. ^{13}C-NMR (150 MHz, CD_3OD) of compound (**1**)-- 4

Figure S3. DEPT (150 MHz, CD_3OD) of compound (**1**)-- 5

Figure S4. ^{1}H-^{1}H COSY (600 MHz, CD_3OD) of compound (**1**) -- 5

Figure S5. HSQC of compound (**1**) -- 6

Figure S6. HMBC of compound (**1**) --- 6

Figure S7. NOESY (600 MHz, CD_3OD) of compound (**1**) -- 7

Figure S8. HR-ESI-MS of compound (**1**) -- 7

Figure S9. ^{1}H-NMR (600 MHz, CD_3OD) of compound (**7**) -- 8

Figure S10. ^{13}C-NMR (150 MHz, CD_3OD) of compound (**7**) -- 8

Figure S11. DEPT (150 MHz, CD_3OD) of compound (**7**) --- 9

Figure S12. ^{1}H-^{1}H COSY (600 MHz, CD_3OD) of compound (**7**)-- 9

Figure S13. HSQC of compound (**7**)---10

Figure S14. HMBC of compound (**7**)--10

Figure S15. NOESY (600 MHz, CD_3OD) of compound (**7**)---11

Figure S16. HR-ESI-MS of compound (**7**)---11

Figure S17. ^{1}H-NMR (400 MHz, CD_3OD) of compound (**2**)--12

Figure S18. ^{13}C-NMR (101 MHz, CD_3OD) of compound (**2**) ---12

Figure S19. HR-ESI-MS of compound (**2**)---13

Figure S20. ^{1}H-NMR (400 MHz, CD_3OD) of compound (**3**)--13

Figure S21. ^{13}C-NMR (101 MHz, CD_3OD) of compound (**3**) ---14

Figure S22. HR-ESI-MS of compound (**3**)---14

Figure S23. ^{1}H-NMR (400 MHz, $CDCl_3$) of compound (**4**) ---15

Figure S24. ^{13}C-NMR (101 MHz, $CDCl_3$) of compound (**4**)---15

Figure S25. HR-ESI-MS of compound (**4**)---16

Figure S26. ^{1}H-NMR (400 MHz, $CDCl_3$) of compound (**5**) ---16

Figure S27. ^{13}C-NMR (101 MHz, $CDCl_3$) of compound (**5**)---17

Figure S28. HR-ESI-MS of compound (**5**)---17

Figure S29. ^{1}H-NMR (400 MHz, $CDCl_3$) of compound (**6**) ---18

Figure S30. ^{13}C-NMR (101 MHz, $CDCl_3$) of compound (**6**)---18

Figure S31. HR-ESI-MS of compound (**6**)---19

Figure S32. ^{1}H-NMR (400 MHz, CD_3OD) of compound (**8**)--19

Figure S33. ^{13}C-NMR (101 MHz, CD_3OD) of compound (**8**) ---20

Figure S34. HR-ESI-MS of compound (**8**)---20

Figure S35. ^{1}H-NMR (400 MHz, $CDCl_3$) of compound (**9**) ---21

Figure S36. ^{13}C-NMR (101 MHz, $CDCl_3$) of compound (**9**)---21

Figure S37. HR-ESI-MS of compound (**9**)--22
Figure S38. ^{1}H-NMR (400 MHz, CD_3OD) of compound (**10**) ---22
Figure S39. ^{13}C-NMR (101 MHz, CD_3OD) of compound (**10**)---23
Figure S40. HR-ESI-MS of compound (**10**) ---23
Figure S41. ^{1}H-NMR (400 MHz, CD_3OD) of compound (**11**) ---24
Figure S42. ^{13}C-NMR (101 MHz, CD_3OD) of compound (**11**)---24
Figure S43. HR-ESI-MS of compound (**11**) ---25
Figure S44. ^{1}H-NMR (400 MHz, CD_3OD) of compound (**12**) ---25
Figure S45. ^{13}C-NMR (101 MHz, CD_3OD) of compound (**12**)---26
Figure S46. HR-ESI-MS of compound (**12**) ---26
Figure S47. ^{1}H-NMR (400 MHz, CD_3OD) of compound (**13**) ---27
Figure S48. ^{13}C-NMR (101 MHz, CD_3OD) of compound (**13**)---27
Figure S49. HR-ESI-MS of compound (**13**) ---28
Figure S50. HPLC spectrum for the purity of tested compounds.--28
Figure S51. The loadings plot--35
Table S1. Average colony diameter of left-right configuration (cm) -------------------------------------35
Table S2. Average colony diameter of center-positioned (cm) ---35
Table S3. Colony growth inhibition ratio under VOC exposure---35
Table S4. VOCs of DHS-48 and HHL-101 in mono-culture and co-culture------------------------------36
Table S5. Vip score values (> 1) of VOCs of DHS-48 and HHL-101 in mono-culture and co-culture 38
Table S6. Average colony diameter dynamics of HHL-101 after 10 days on 14 cm PDA plate， with **5**/**6** (0, 50 μM) restricted to the central 1 cm region --39
Table S7. Average colony diameter dynamics of HHL-101 in 9 cm PDA plates treated with compounds **5**/**6** (0, 50 μM) --39
Table S8. Gibbs free energies[a] and equilibrium populations[b] of low-energy conformers of 5*S*,6*S*, 8*S*, 8a*R*, 9*S*, 10a*S*-**1** ---39
Table S9. Cartesian coordinates for the low-energy reoptimized MMFF conformers of 5*S*,6*S*, 8*S*, 8a*R*, 9*S*, 10a*S*-**1** at B3LYP/6-31G(d,p) level of theory in gas--40
Table S10. Gibbs free energies[a] and equilibrium populations[b] of low-energy conformers of 3*R*, 8*R*-**7** ---43
Table S11. Cartesian coordinates for the low-energy reoptimized MMFF conformers of 3*R*, 8*R*-**7** at B3LYP/6-31G(d,p) level of theory in gas --43

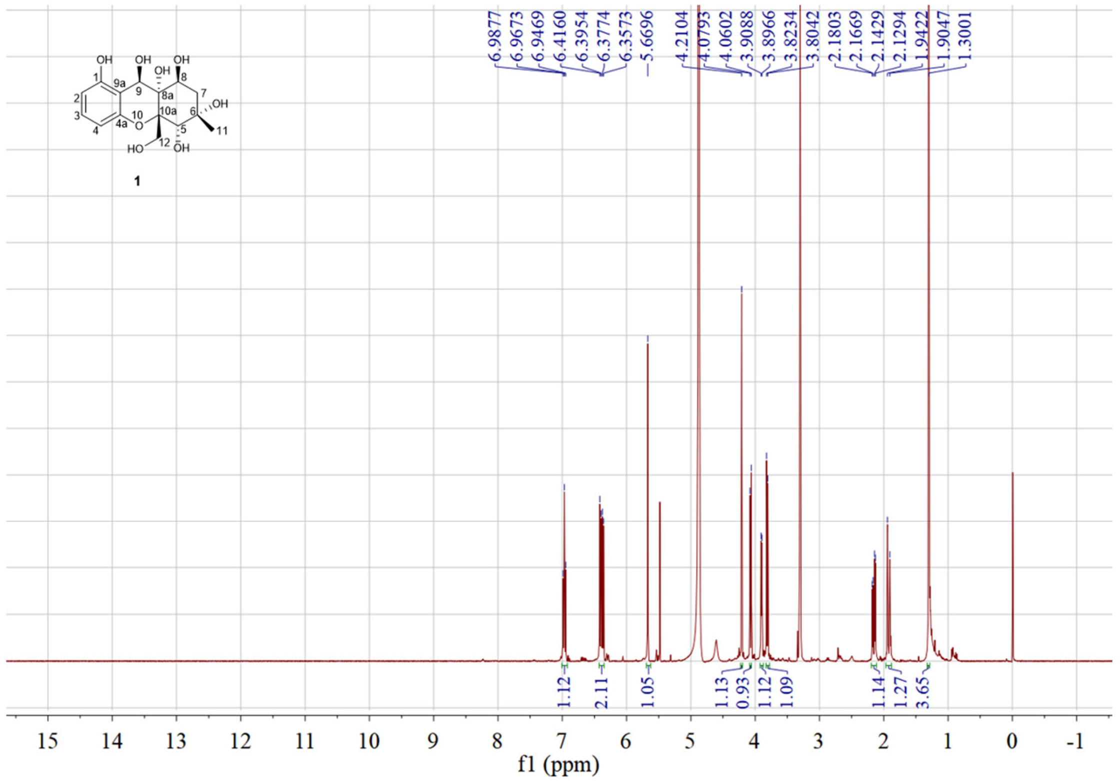

Figure S1. ^{1}H-NMR (600 MHz, CD_3OD) of compound (**1**)

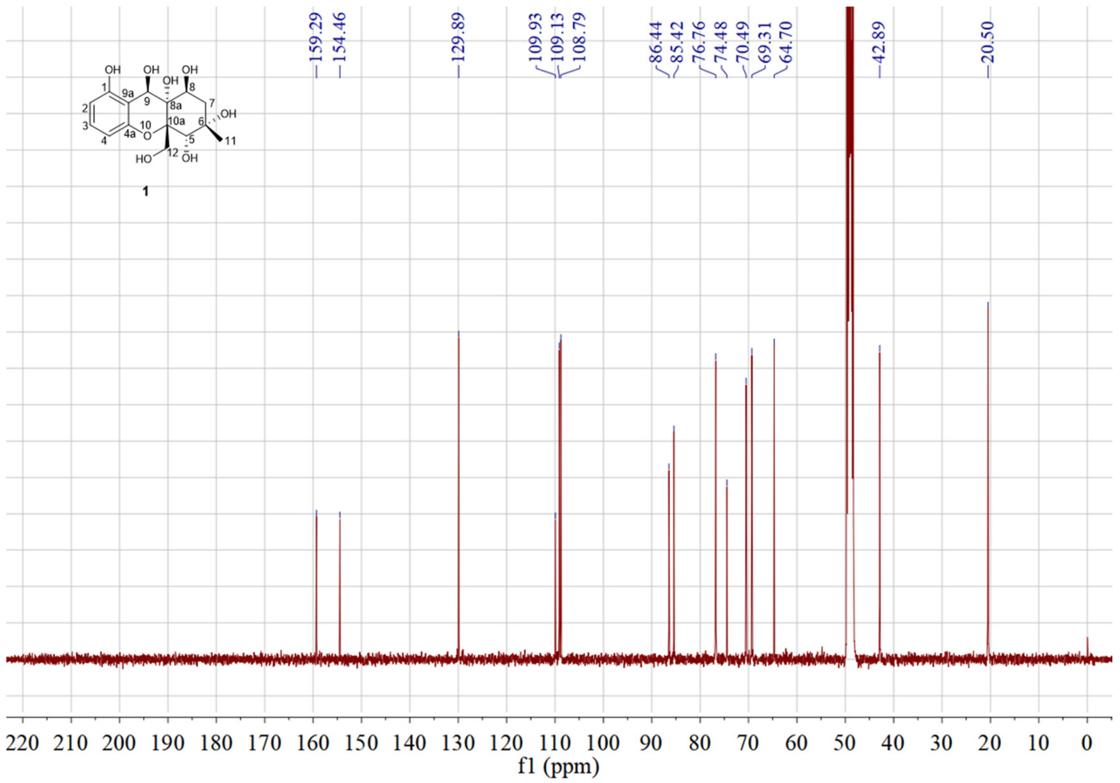

Figure S2. ^{13}C-NMR (150 MHz, CD_3OD) of compound (**1**)

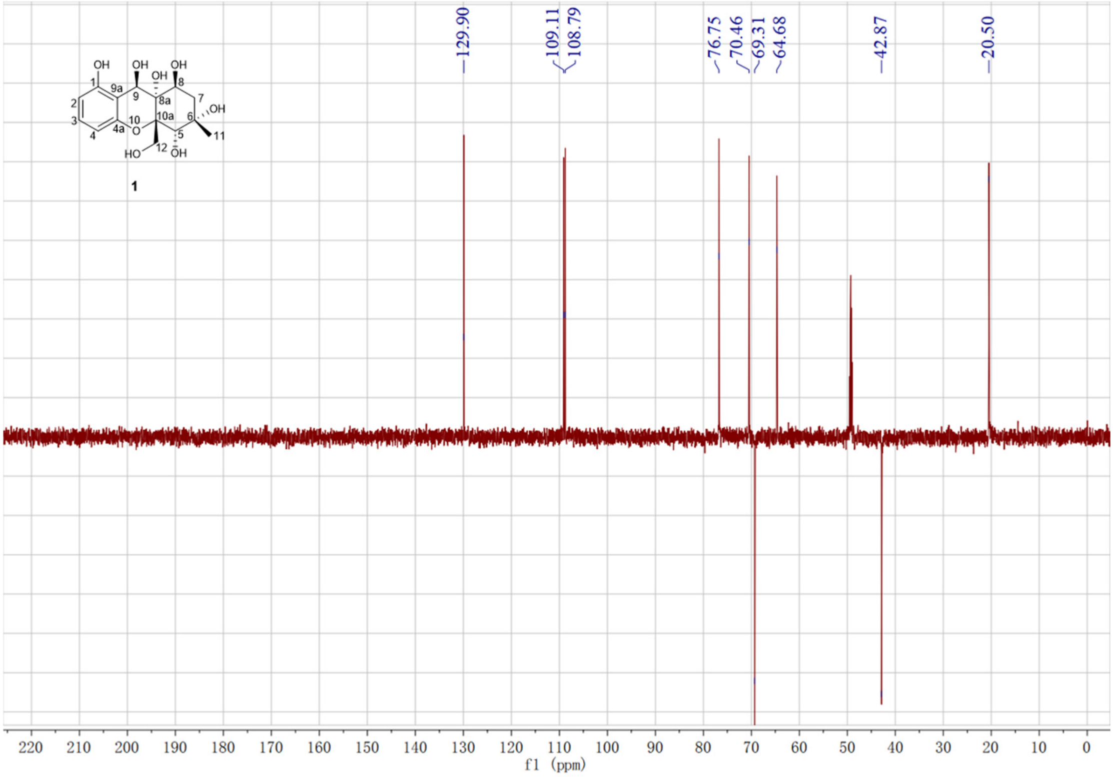

Figure S3. DEPT (150 MHz, CD3OD) of compound (**1**)

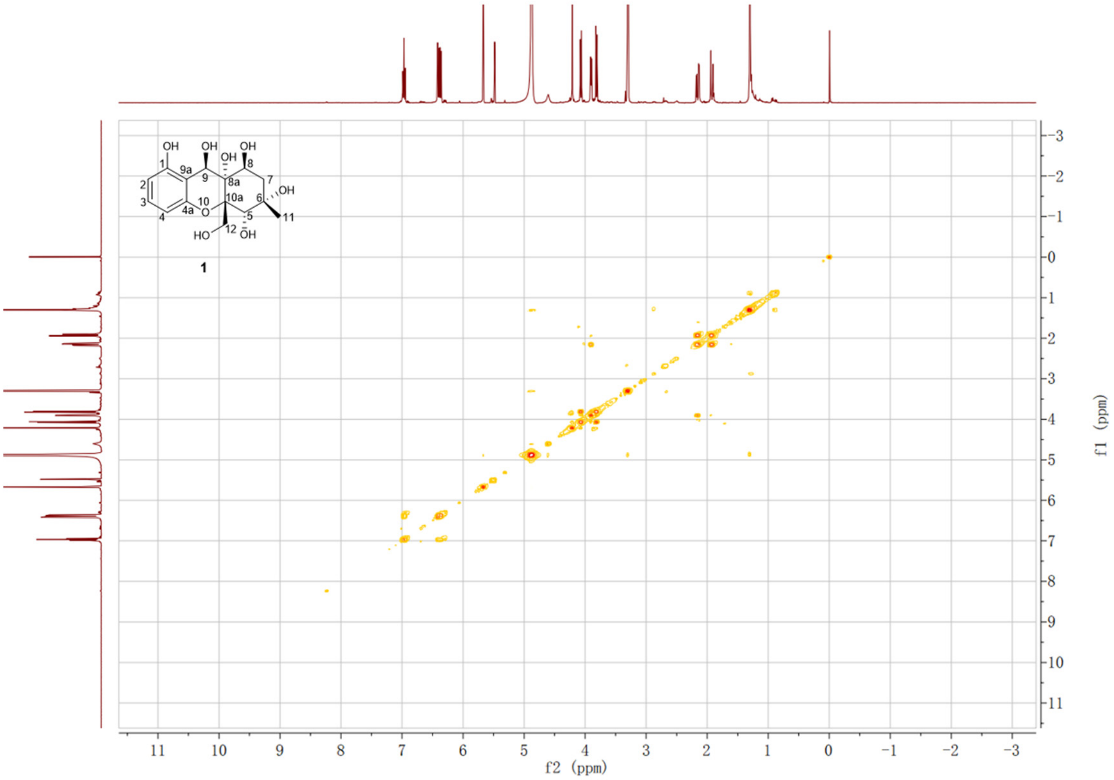

Figure S4. ^{1}H-^{1}H COSY (600 MHz, CD3OD) of compound (**1**)

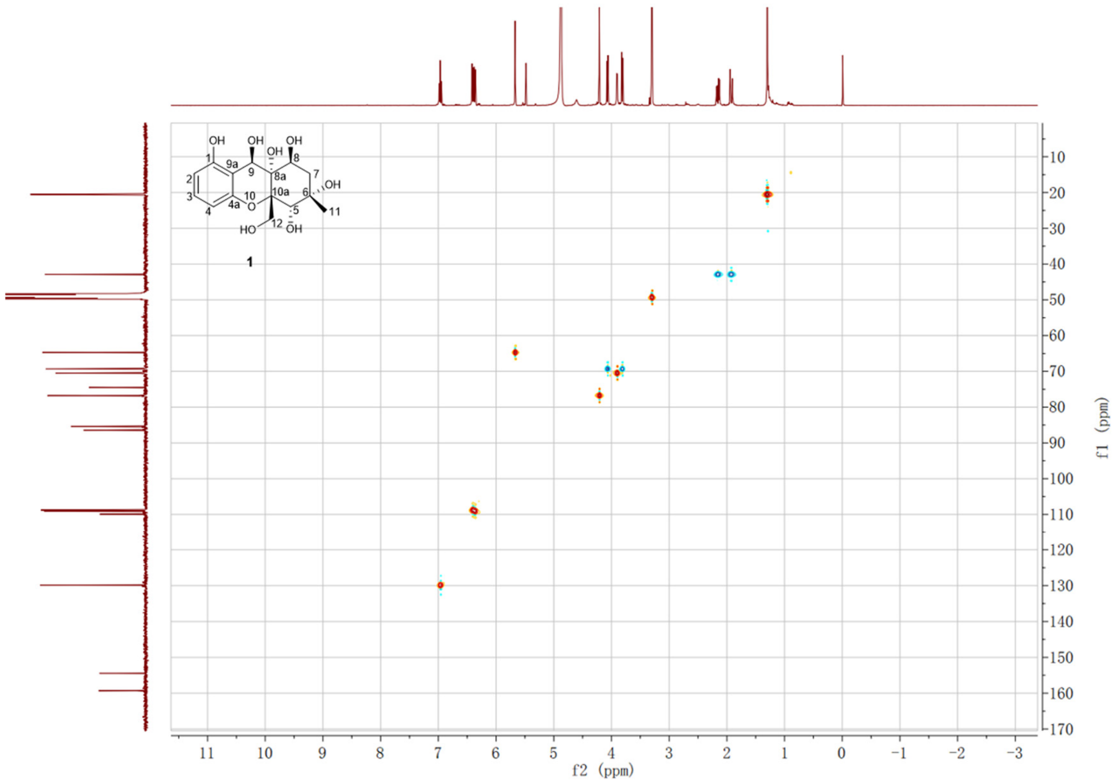

Figure S5. HSQC of compound (**1**)

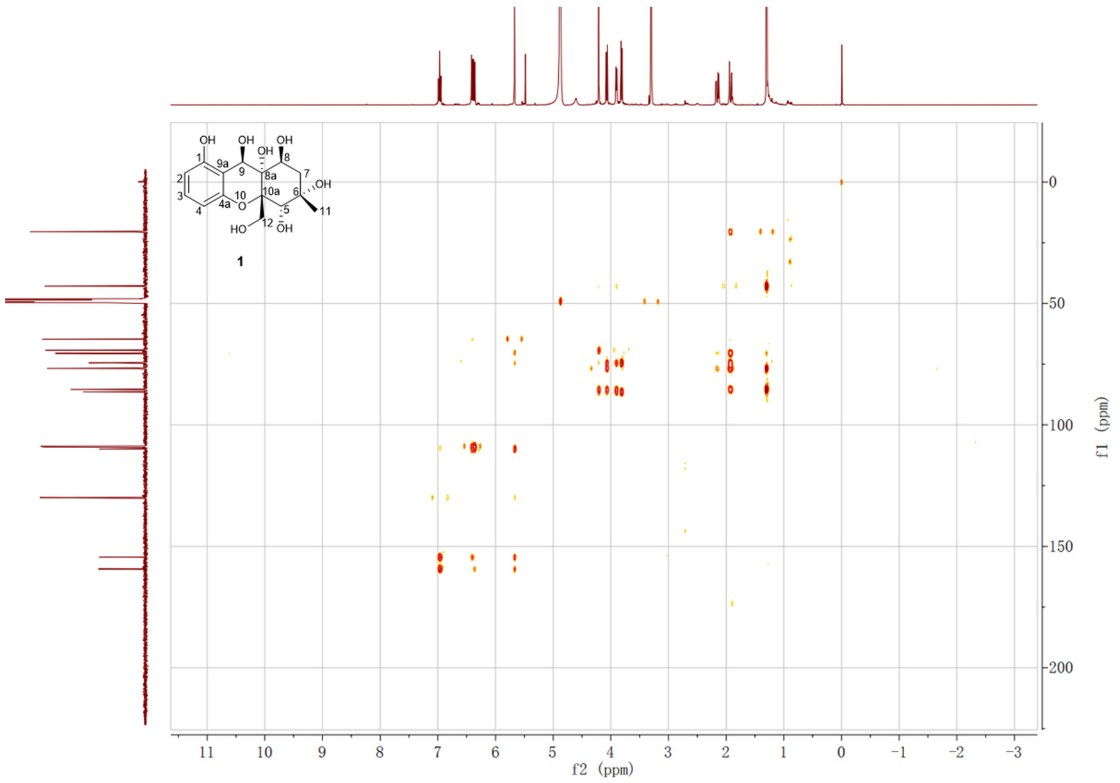

Figure S6. HMBC of compound (**1**)

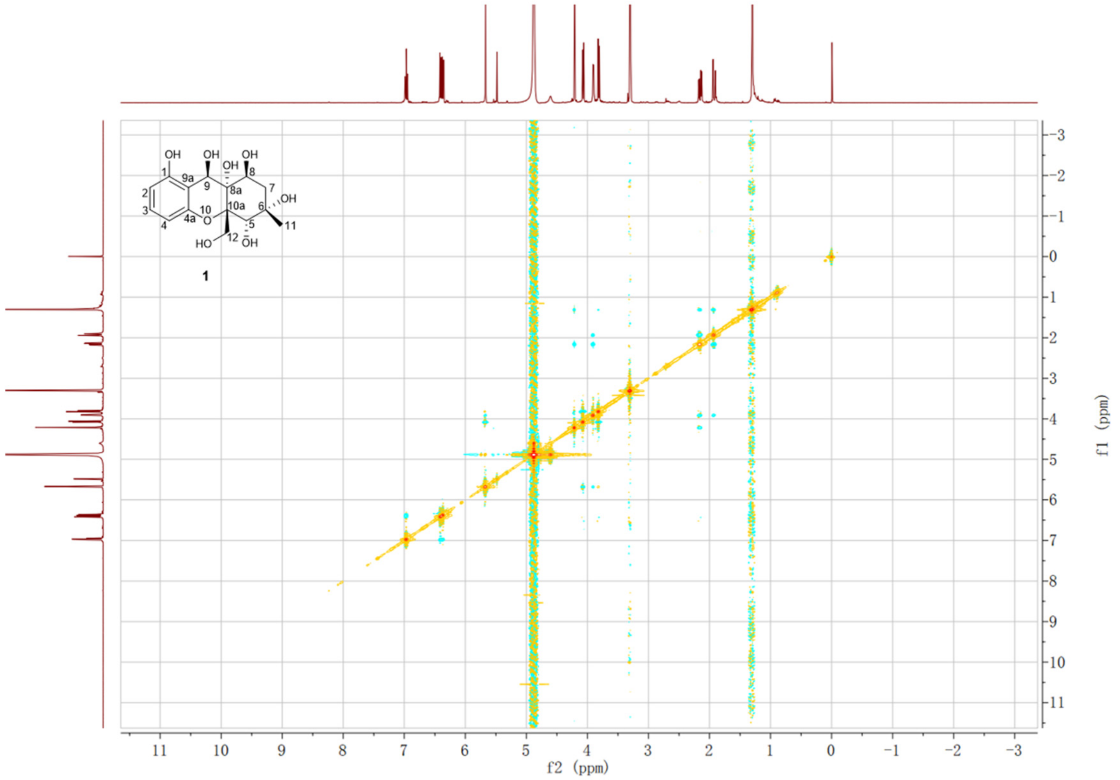

Figure S7. NOESY (600 MHz, CD_3OD) of compound (**1**)

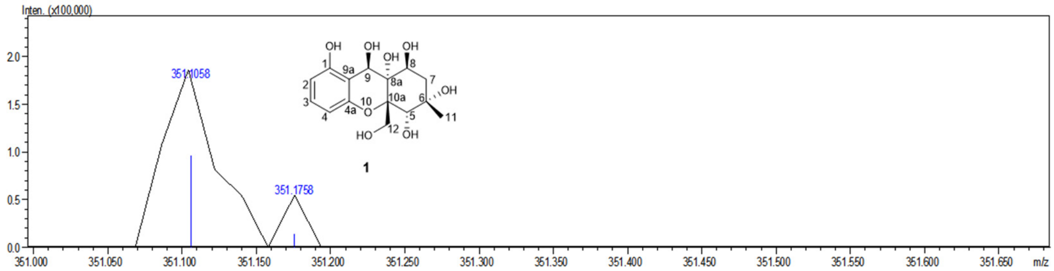

Figure S8. HR-ESI-MS of compound (**1**)

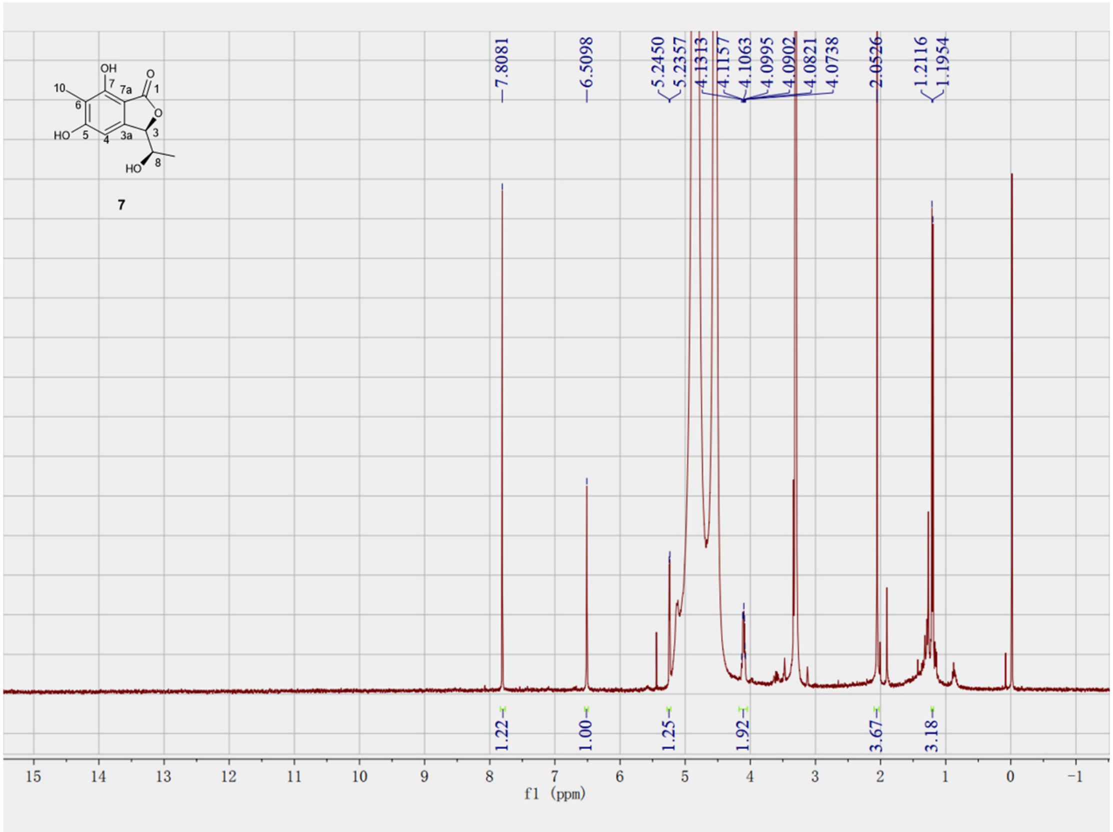

Figure S9. ^{1}H-NMR (600 MHz, CD_3OD) of compound (**7**)

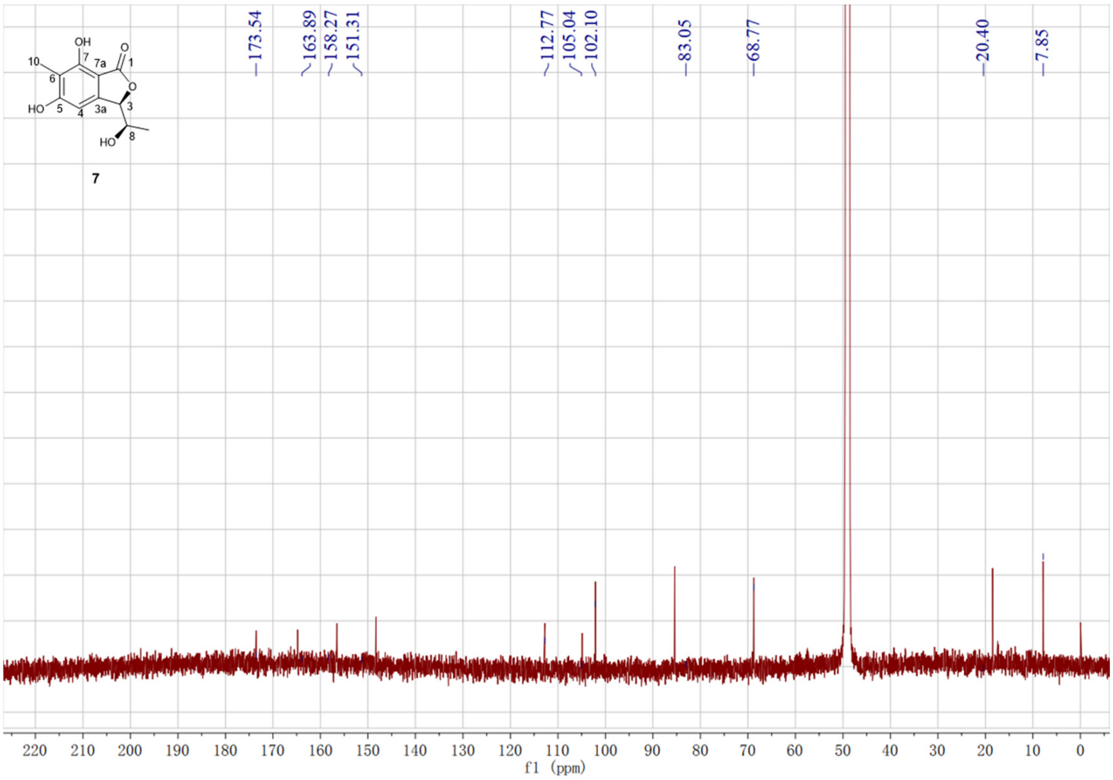

Figure S10. ^{13}C-NMR (150 MHz, CD_3OD) of compound (**7**)

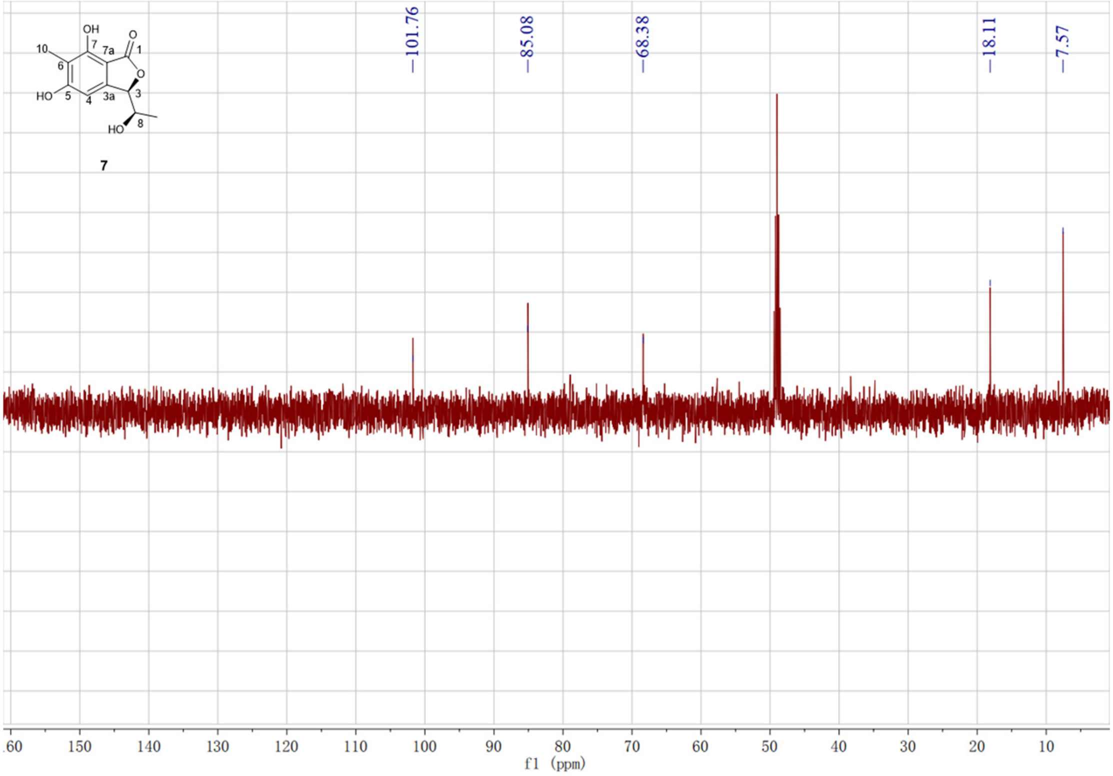

Figure S11. DEPT (150 MHz, CD_3OD) of compound (**7**)

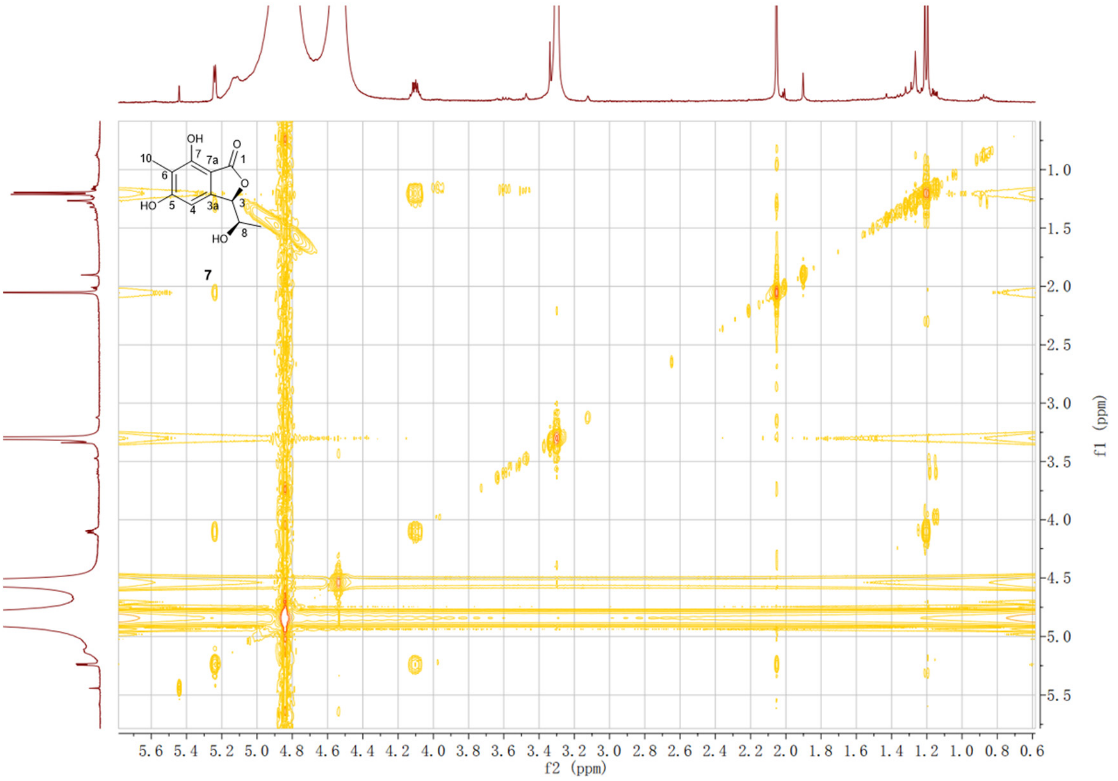

Figure S12. ^{1}H-^{1}H COSY (600 MHz, CD_3OD) of compound (**7**)

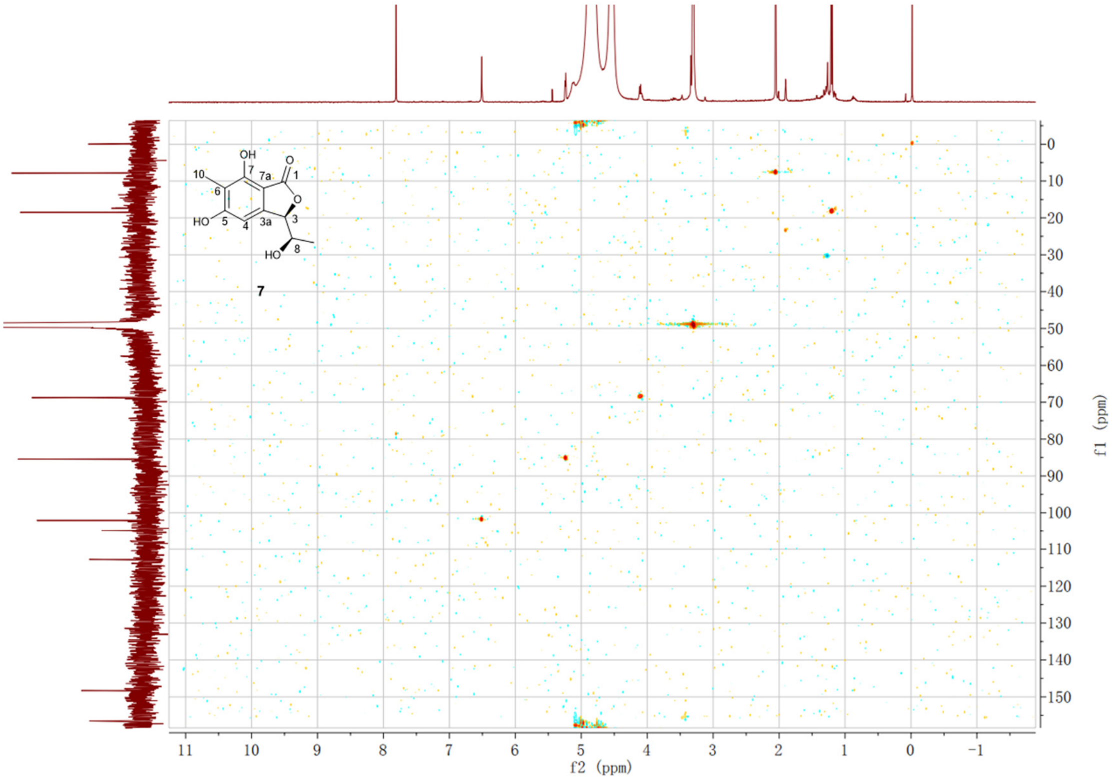

Figure S13. HSQC of compound (**7**)

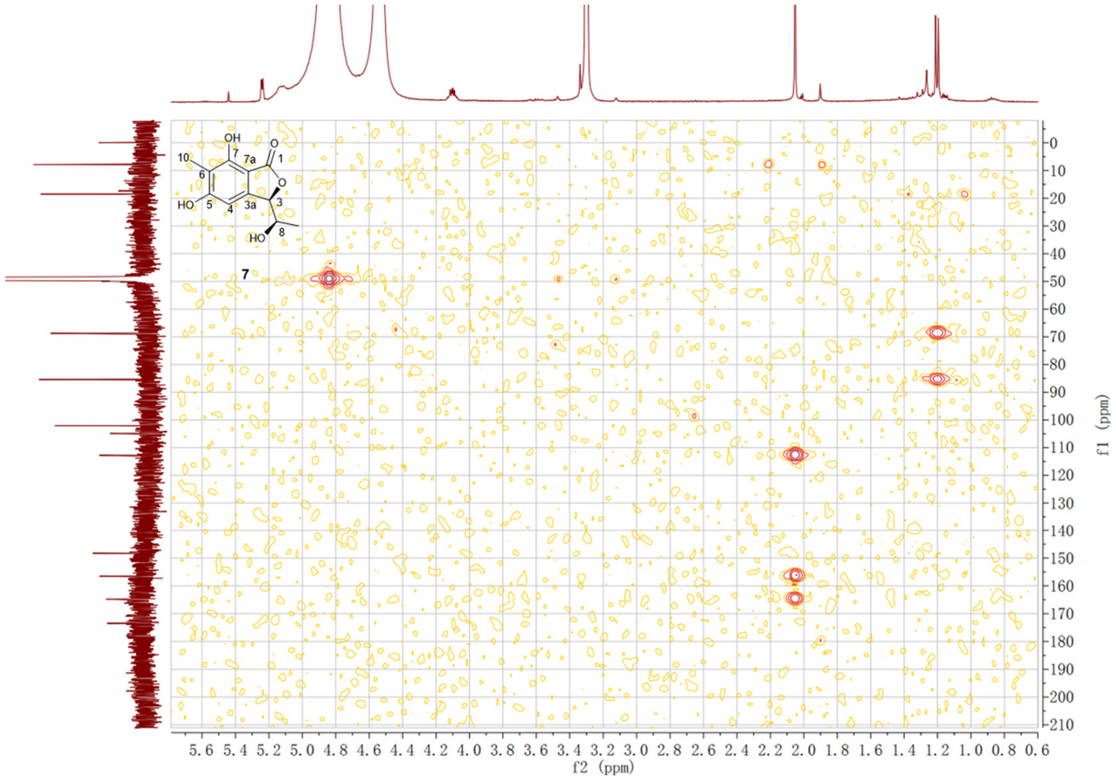

Figure S14. HMBC of compound (**7**)

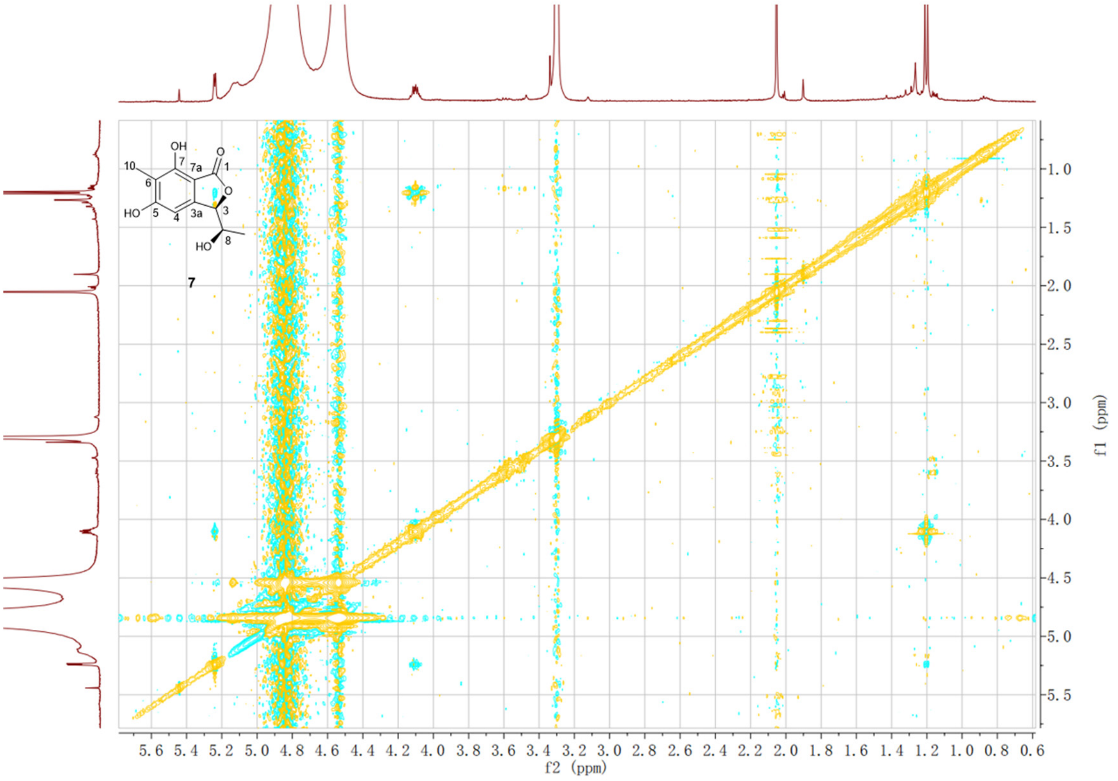

Figure S15. NOESY (600 MHz, CD_3OD) of compound (**7**)

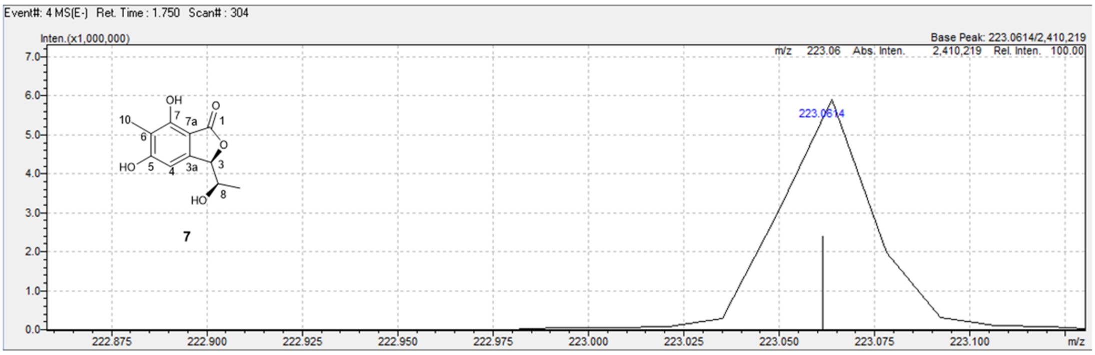

Figure S16. HR-ESI-MS of compound (**7**)

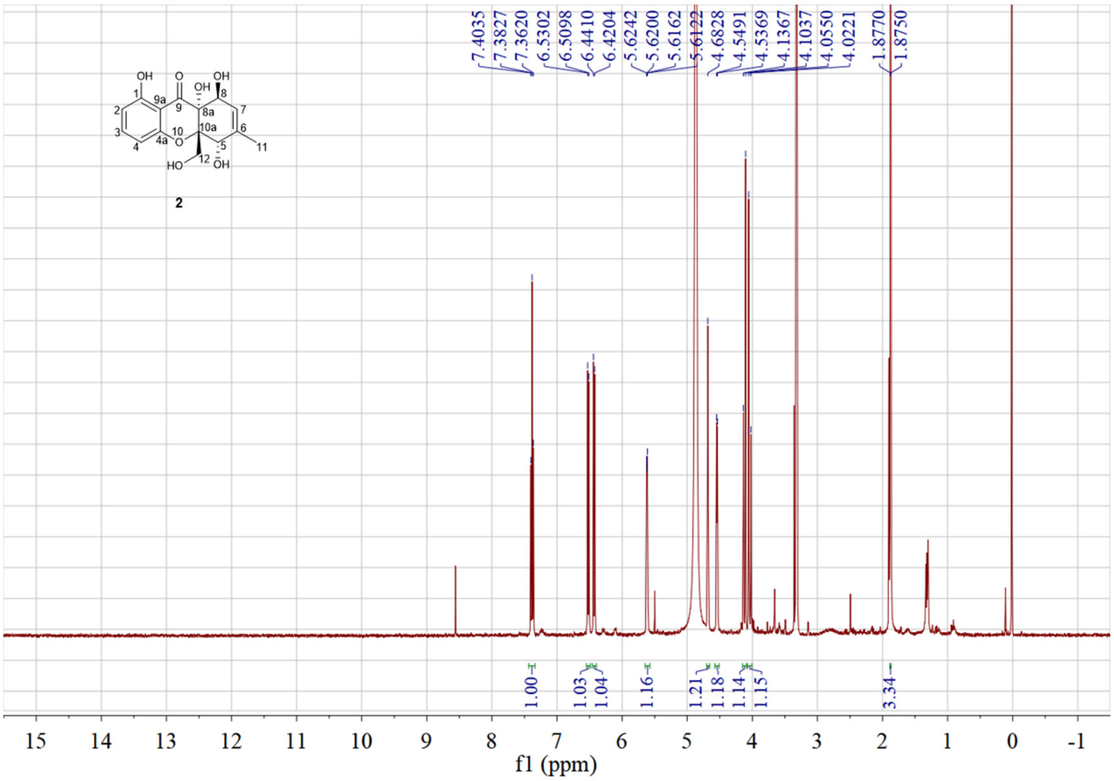

Figure S17. ^{1}H-NMR (400 MHz, CD_3OD) of compound (**2**)

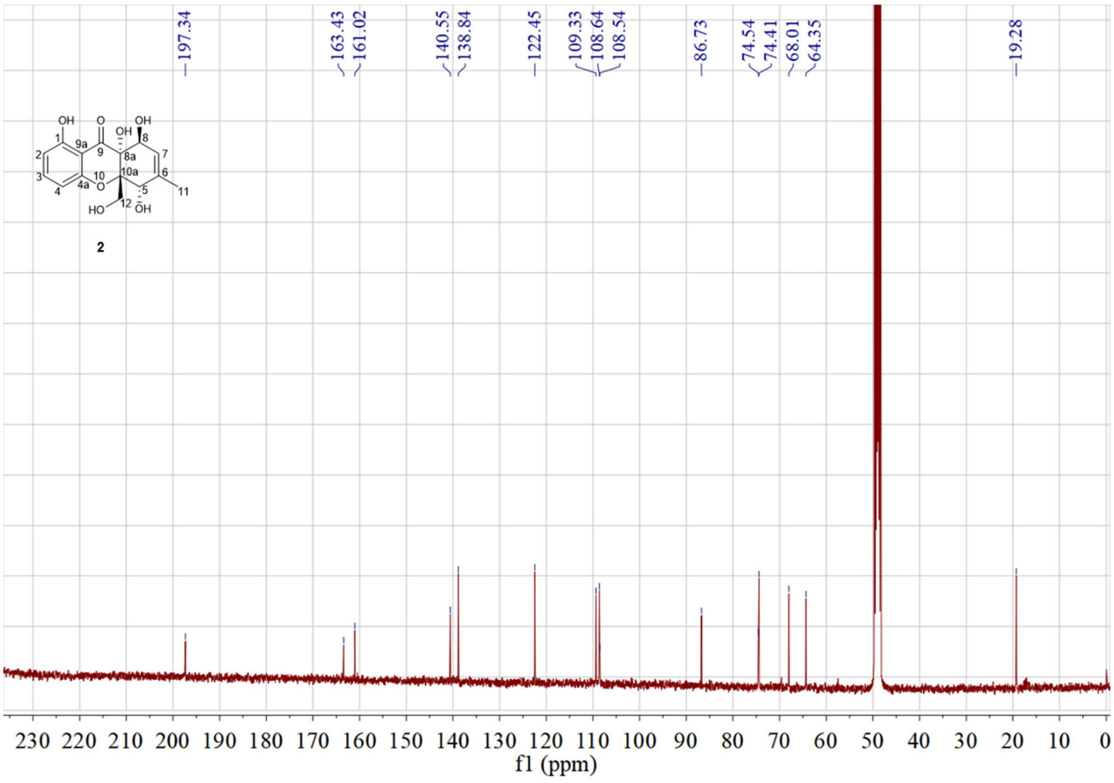

Figure S18. ^{13}C-NMR (101 MHz, CD$_3$OD) of compound (**2**)

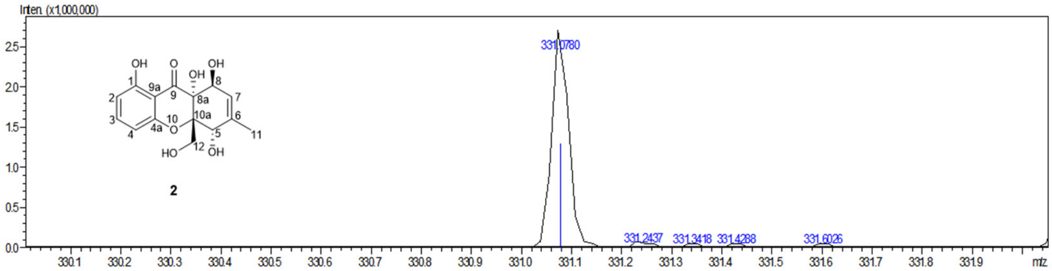

Figure S19. HR-ESI-MS of compound (**2**)

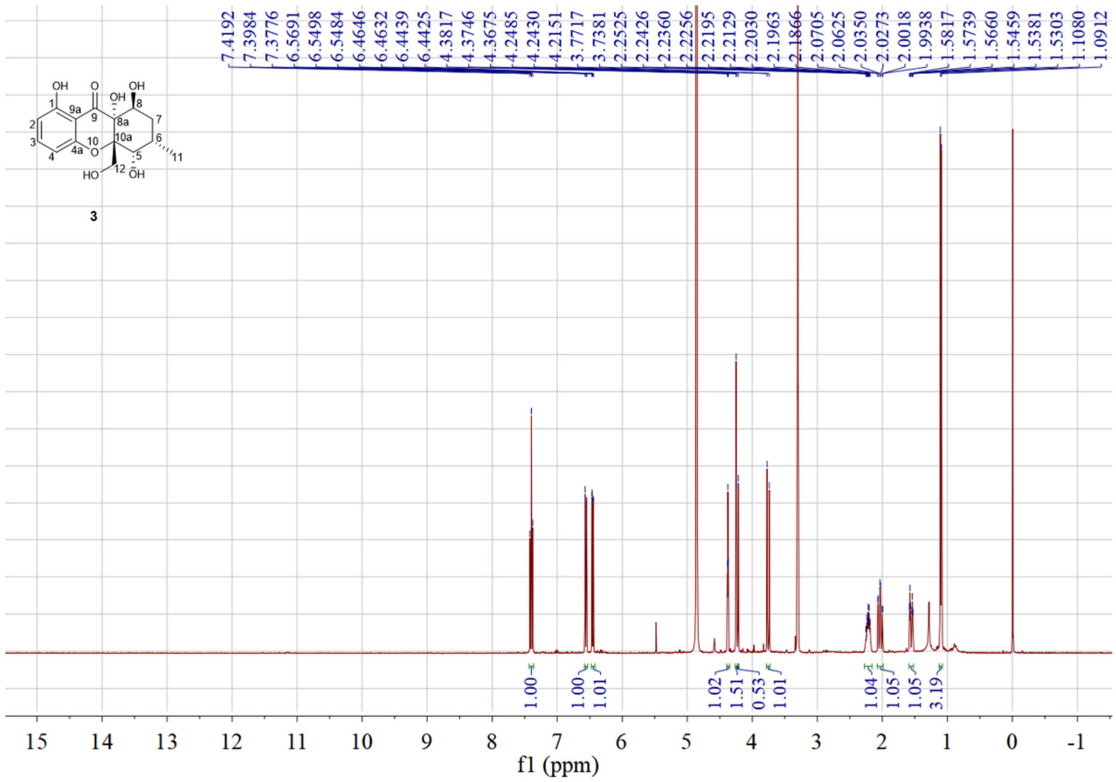

Figure S20. ^{1}H-NMR (400 MHz, CD$_3$OD) of compound (**3**)

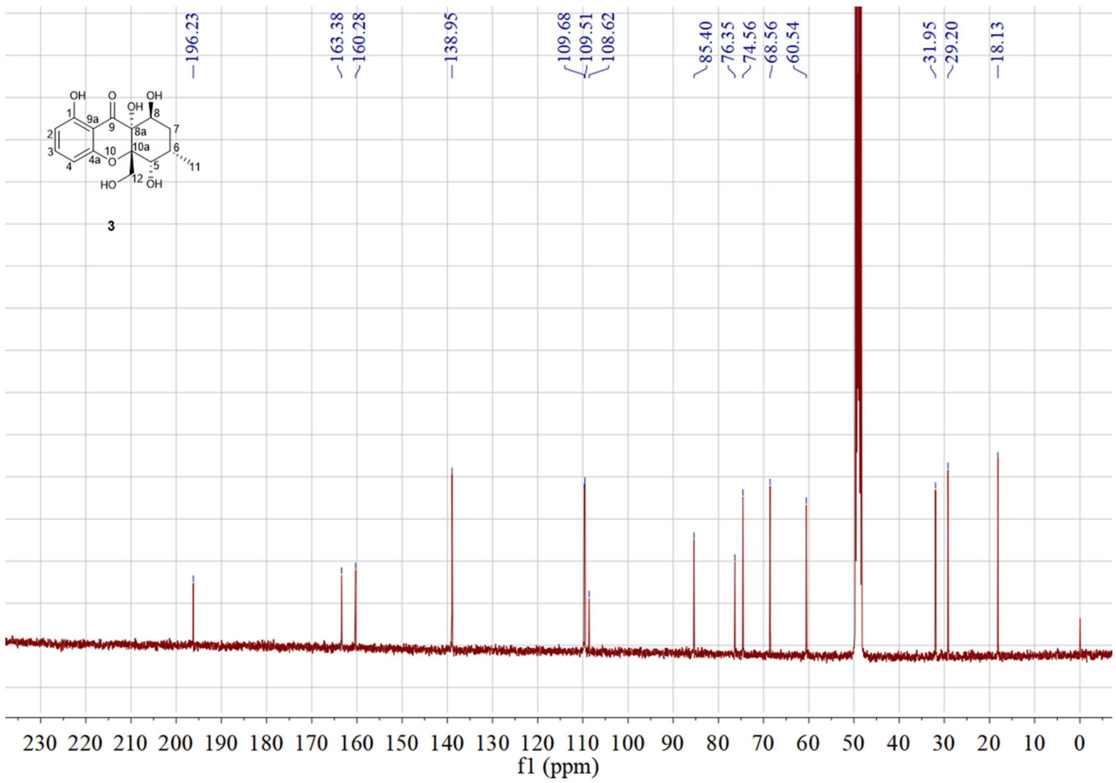

Figure S21. ^{13}C-NMR (101 MHz, CD_3OD) of compound (**3**)

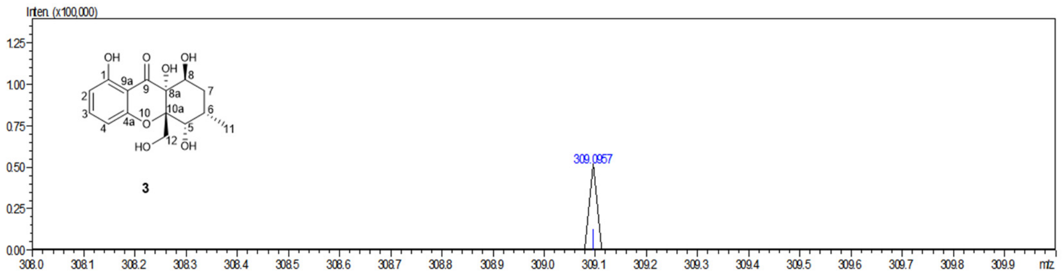

Figure S22. HR-ESI-MS of compound (**3**)

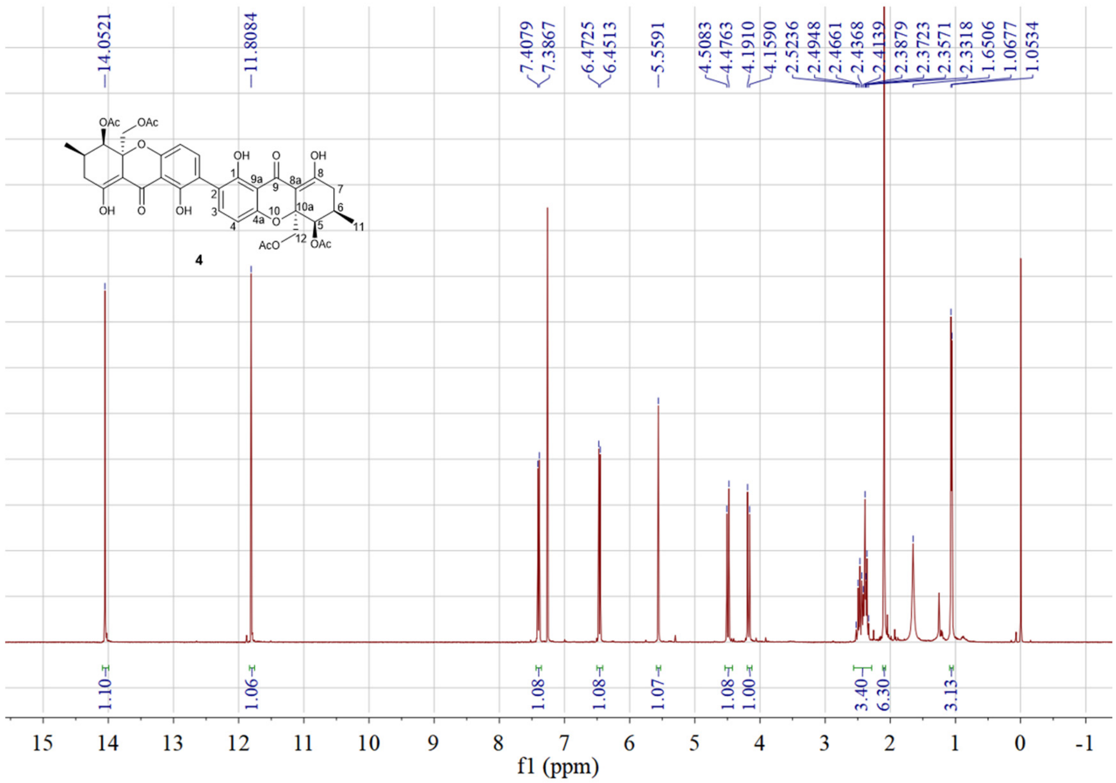

Figure S23. ^{1}H-NMR (400 MHz, $CDCl_3$) of compound (**4**)

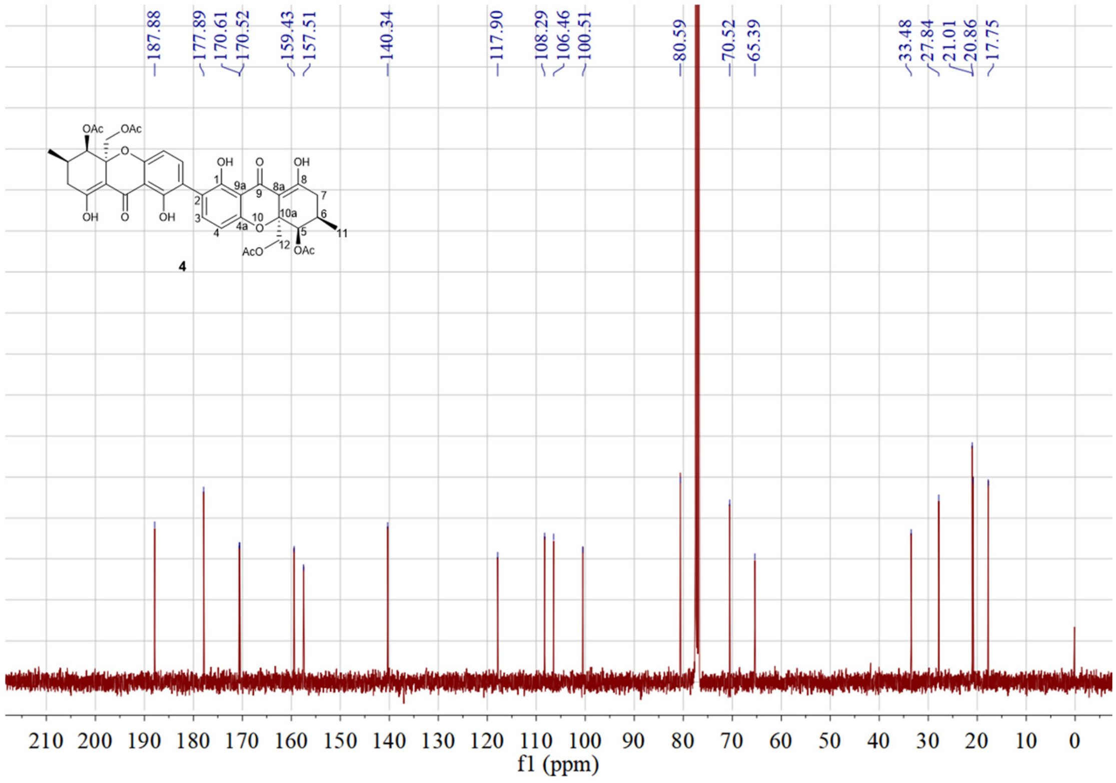

Figure S24. ^{13}C-NMR (101 MHz, CDCl$_3$) of compound (**4**)

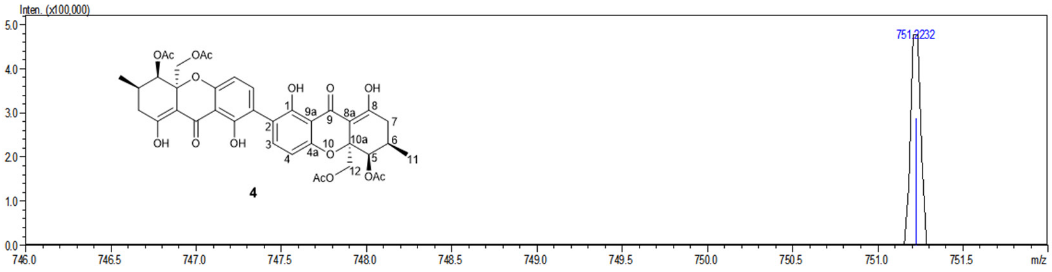

Figure S25. HR-ESI-MS of compound (**4**)

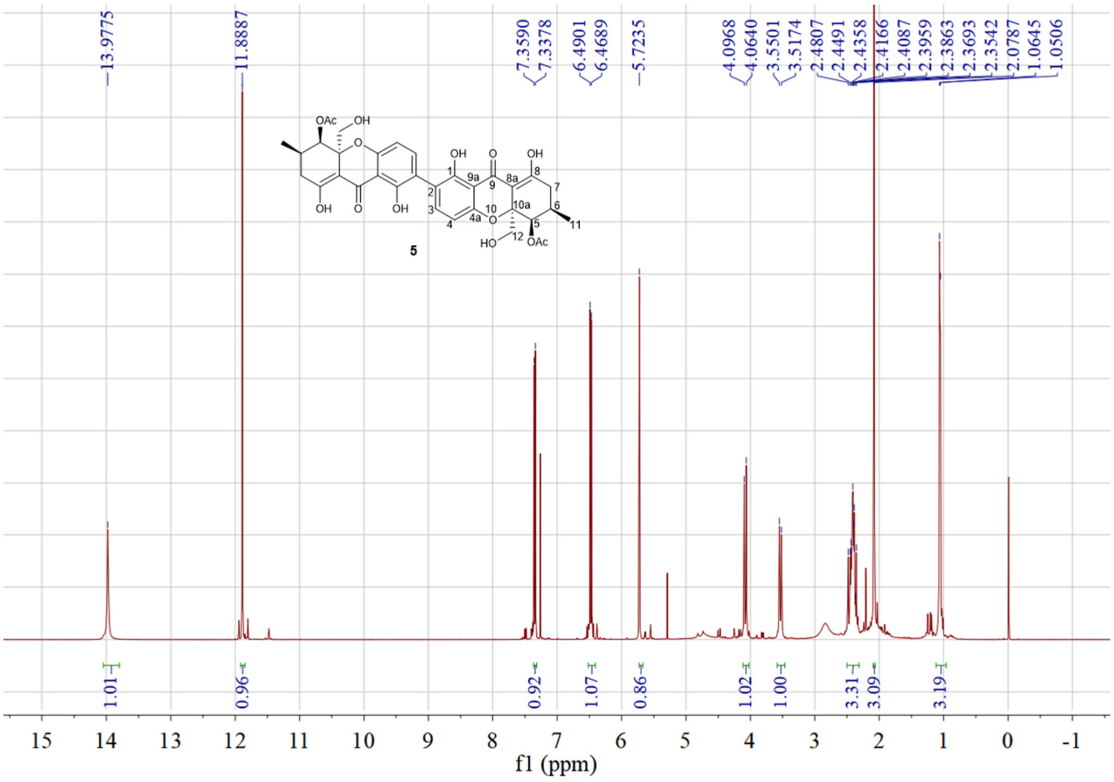

Figure S26. ^{1}H-NMR (400 MHz, CDCl$_3$) of compound (**5**)

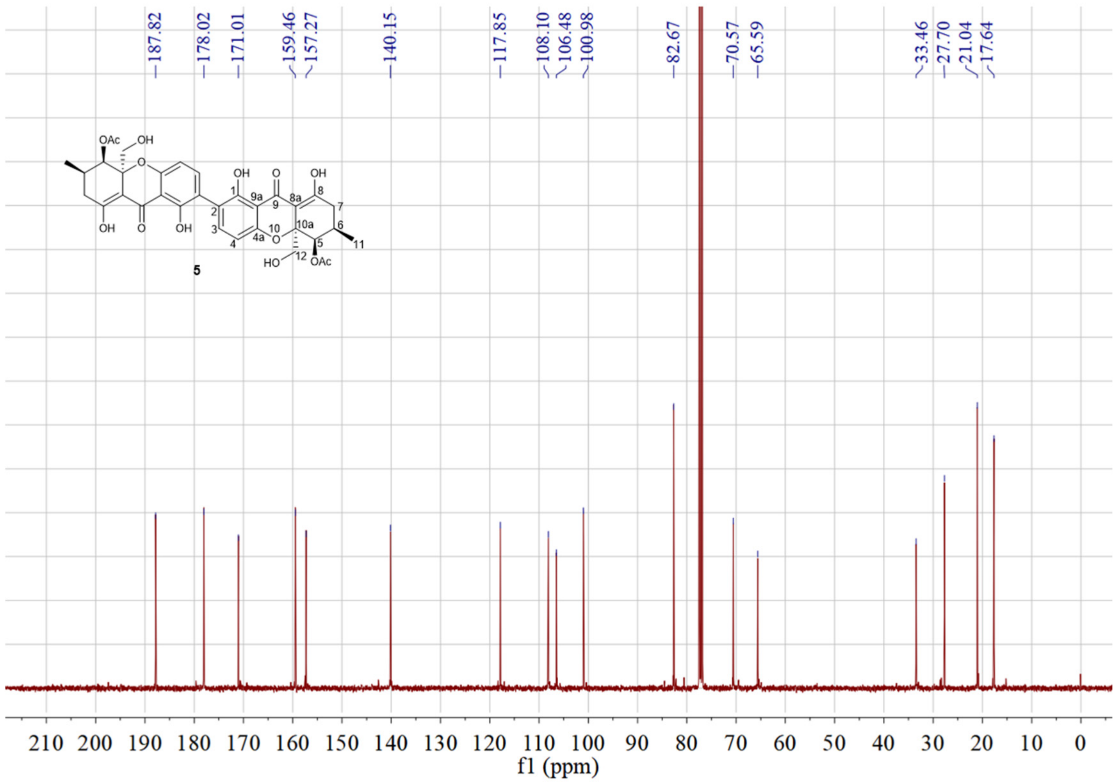

Figure S27. ^{13}C-NMR (101 MHz, $CDCl_3$) of compound (**5**)

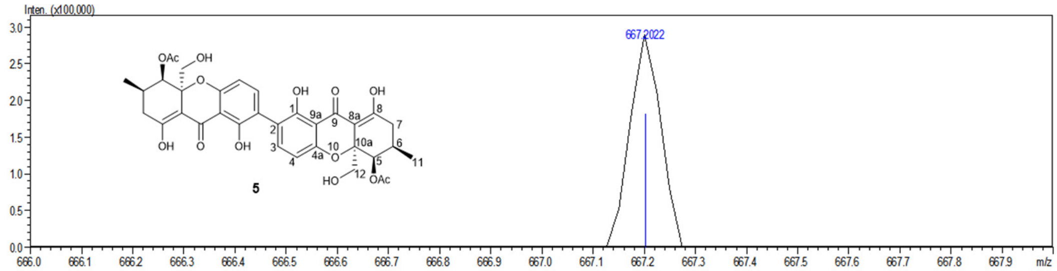

Figure S28. HR-ESI-MS of compound (**5**)

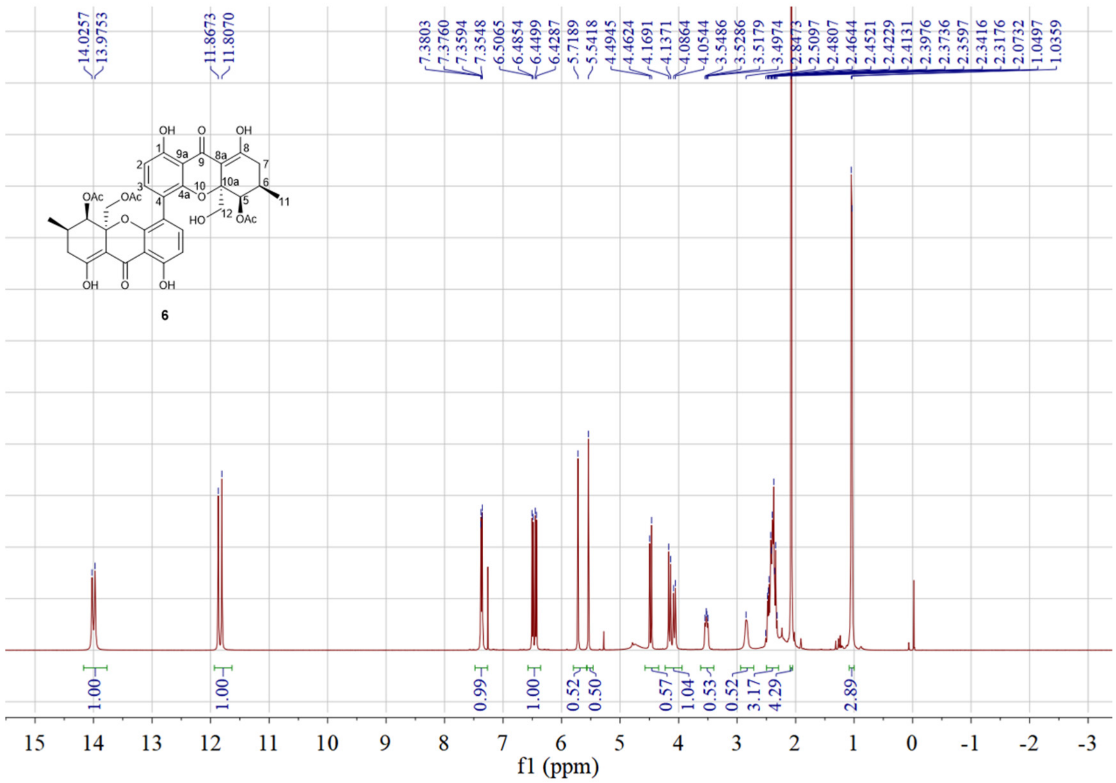

Figure S29. ^{1}H-NMR (400 MHz, $CDCl_3$) of compound (**6**)

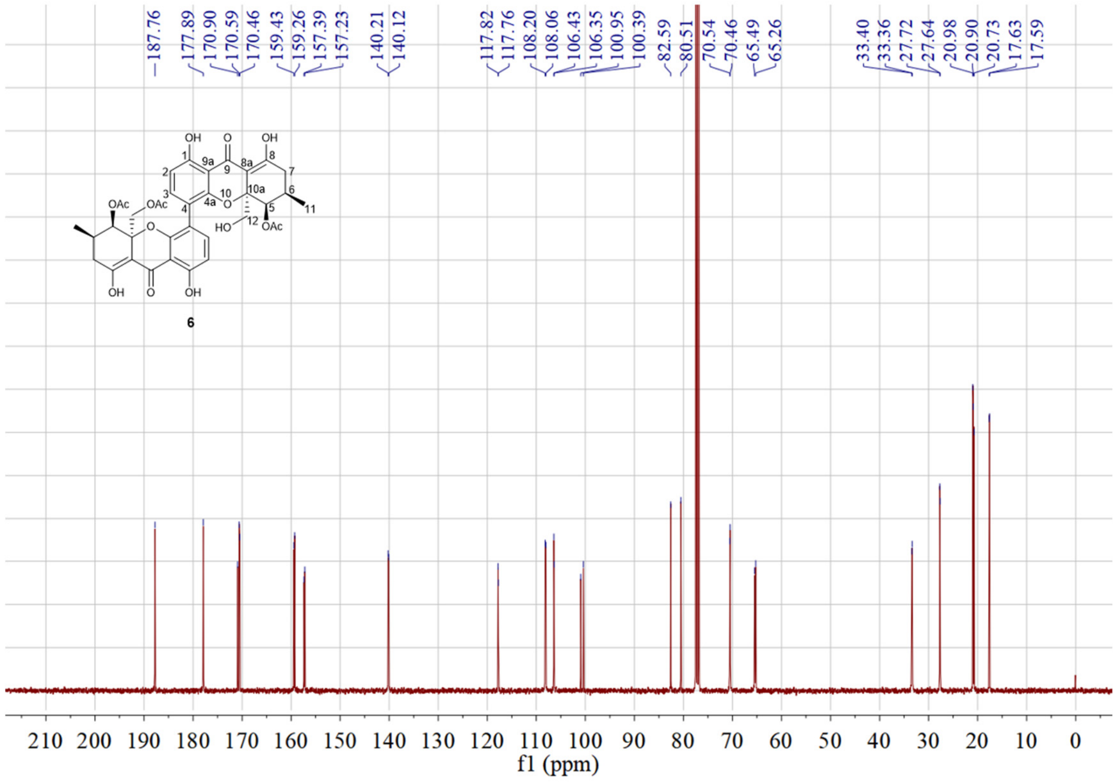

Figure S30. ^{13}C-NMR (101 MHz, $CDCl_3$) of compound (**6**)

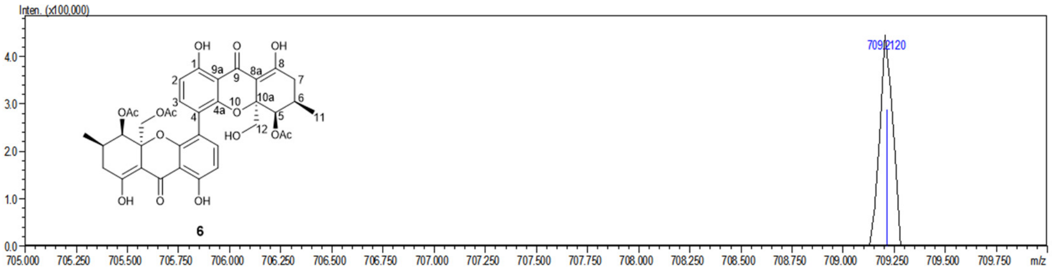

Figure S31. HR-ESI-MS of compound (**6**)

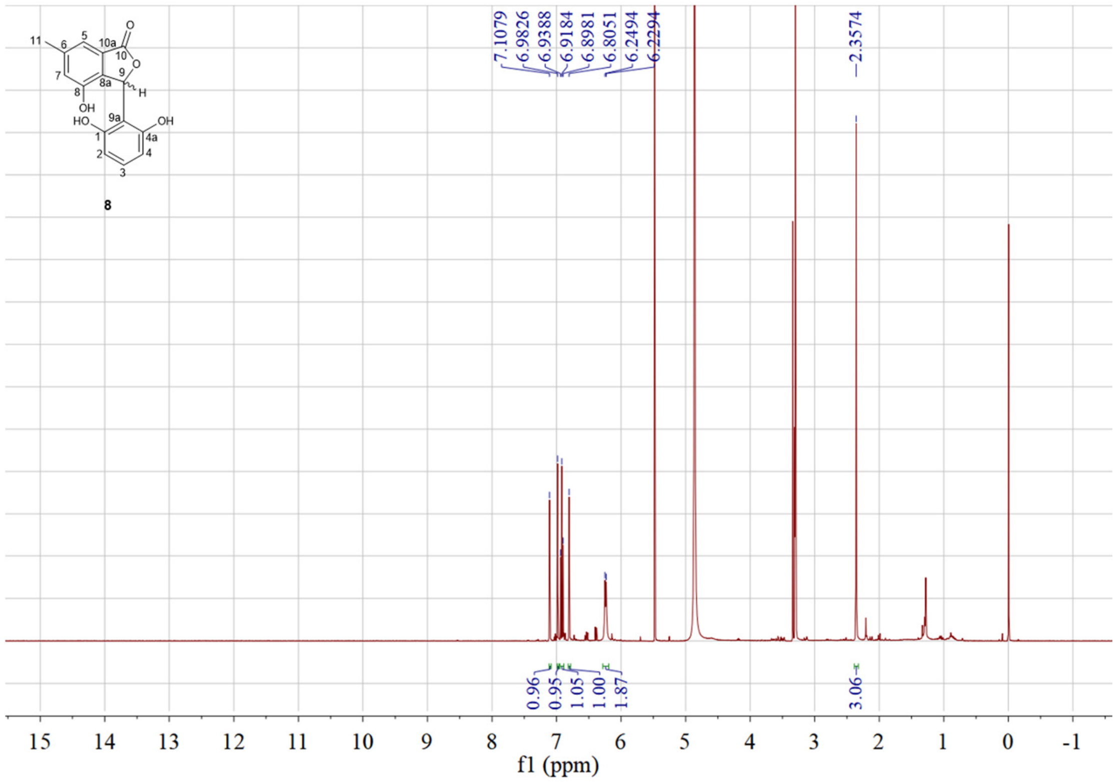

Figure S32. ^{1}H-NMR (400 MHz, CD_3OD) of compound (**8**)

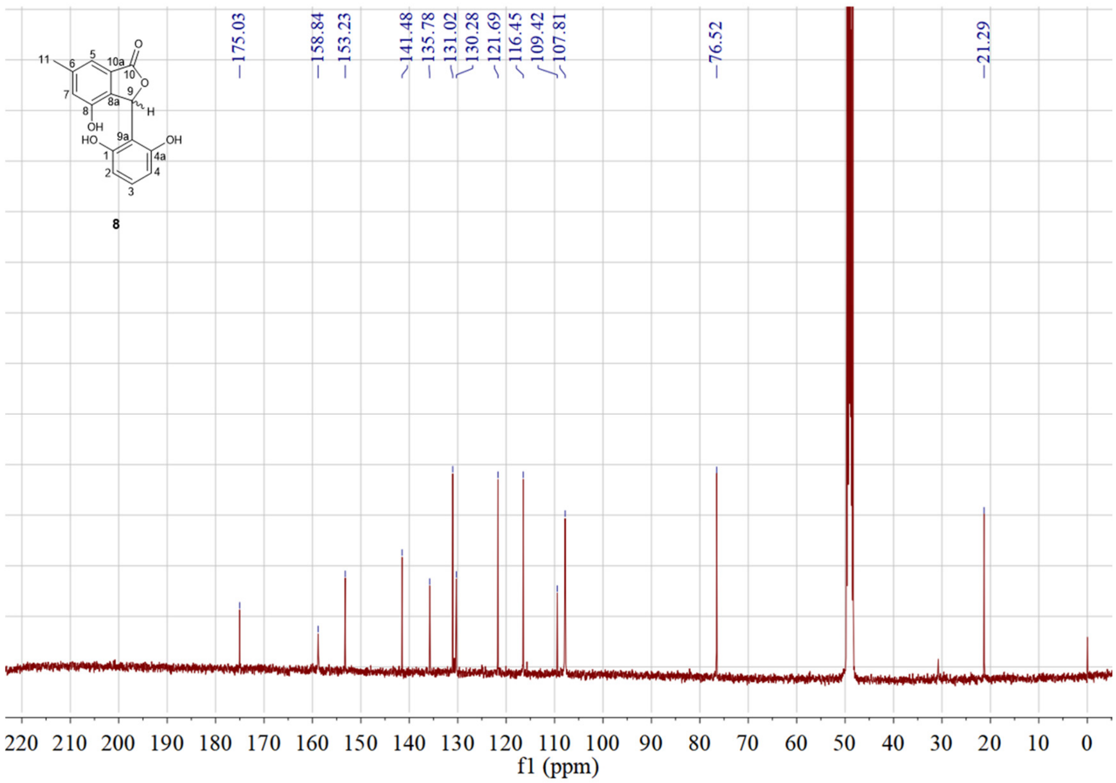

Figure S33. ^{13}C-NMR (101 MHz, CD_3OD) of compound (**8**)

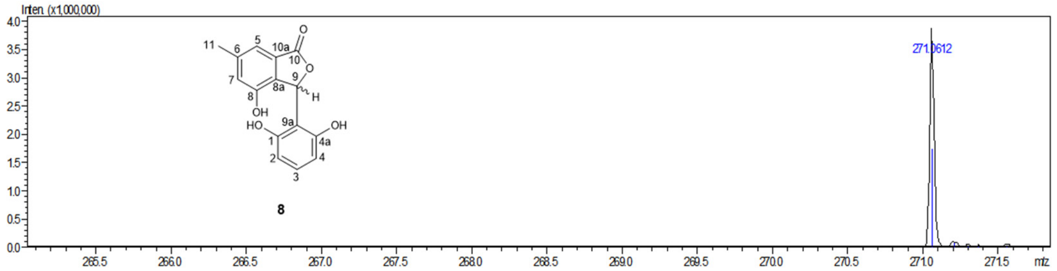

Figure S34. HR-ESI-MS of compound (**8**)

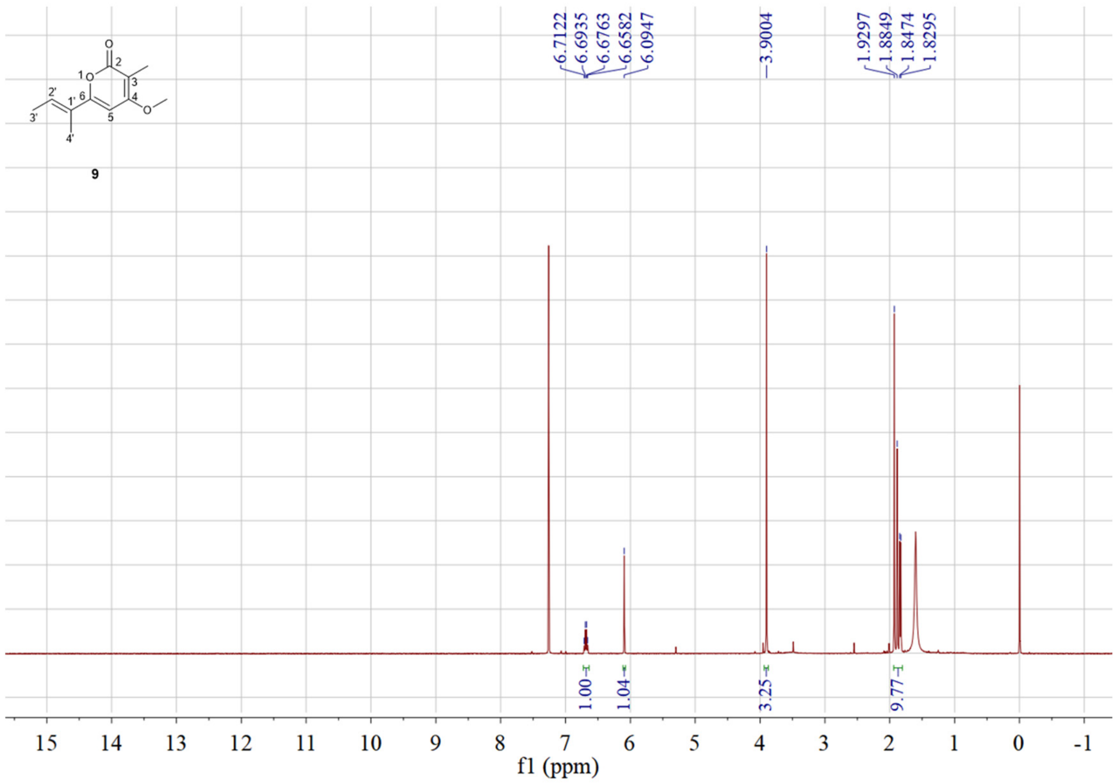

Figure S35. ^{1}H-NMR (400 MHz, $CDCl_3$) of compound (**9**)

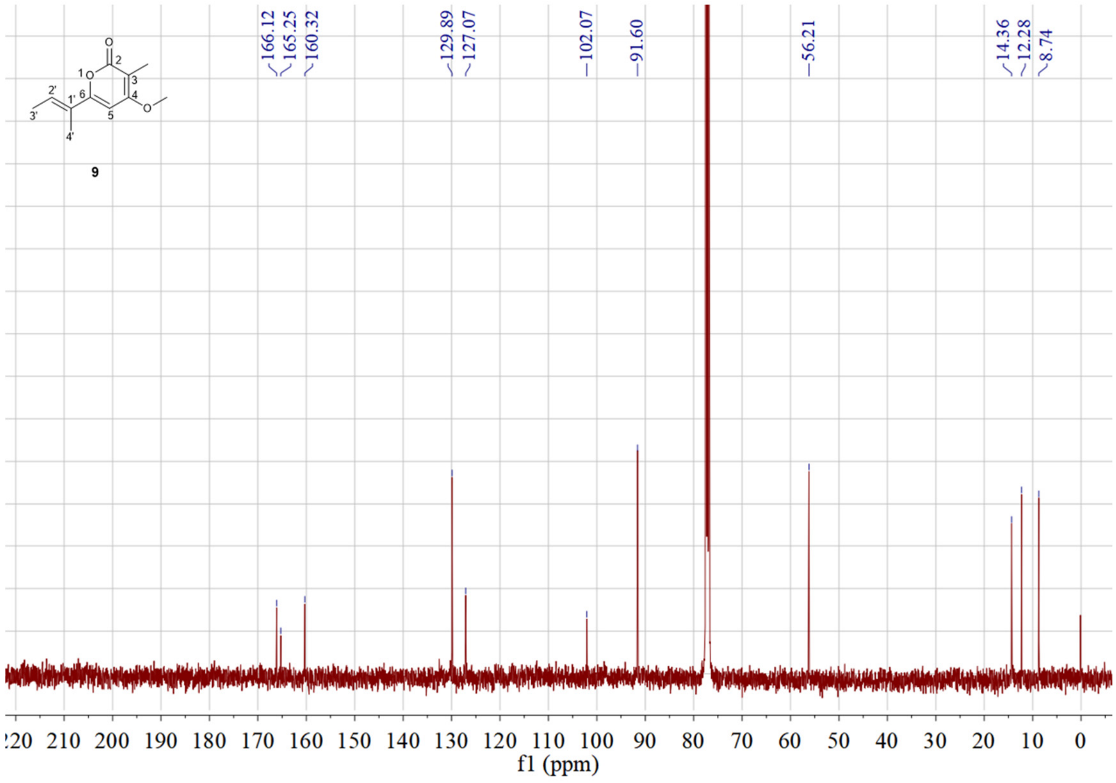

Figure S36. ^{13}C-NMR (101 MHz, $CDCl_3$) of compound (**9**)

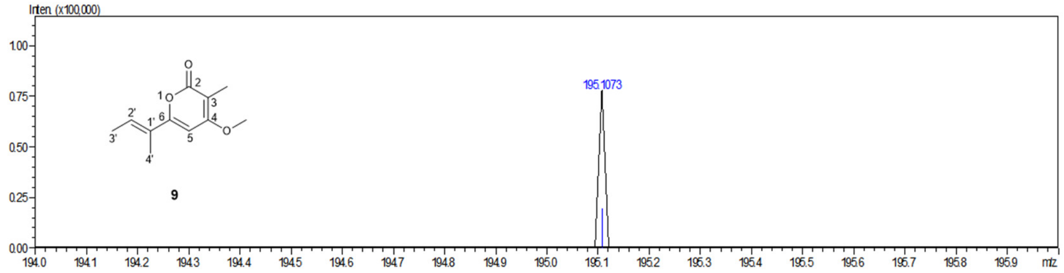

Figure S37. HR-ESI-MS of compound (**9**)

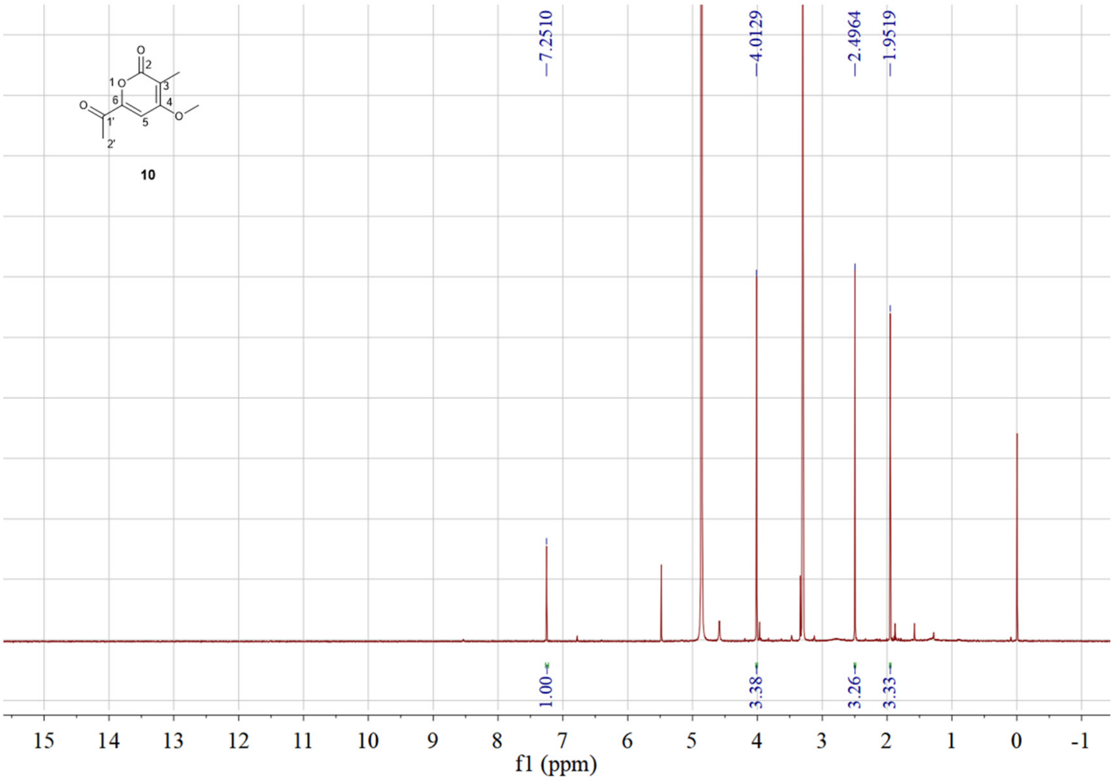

Figure S38. ^{1}H-NMR (400 MHz, CD_3OD) of compound (**10**)

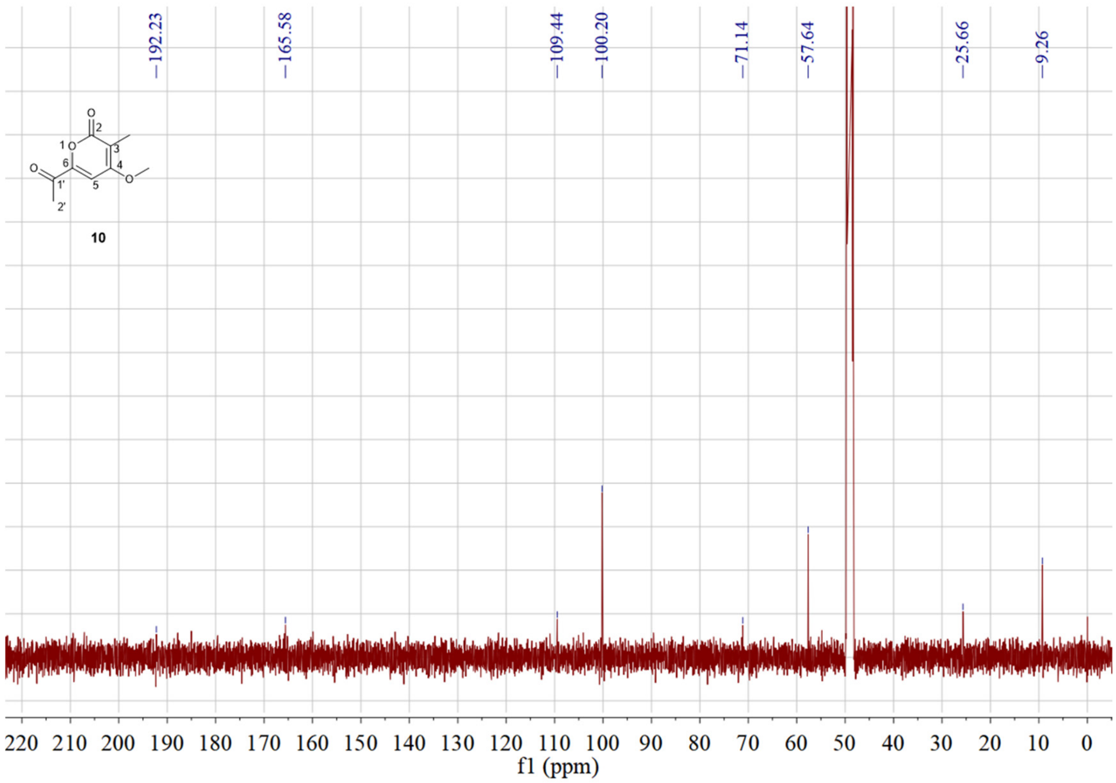

Figure S39. ^{13}C-NMR (101 MHz, CD_3OD) of compound (**10**)

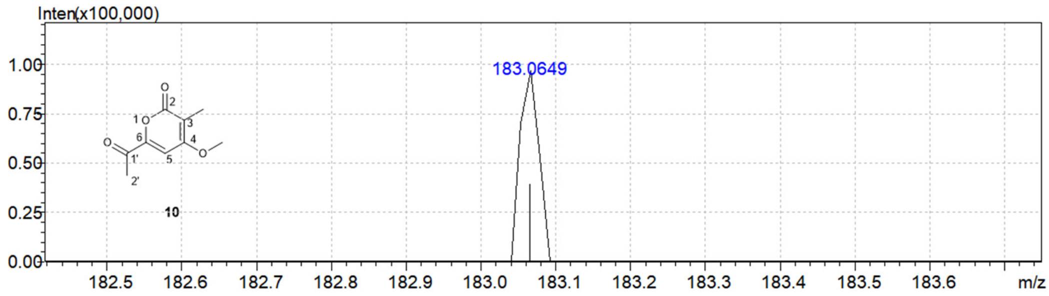

Figure S40. HR-ESI-MS of compound (**10**)

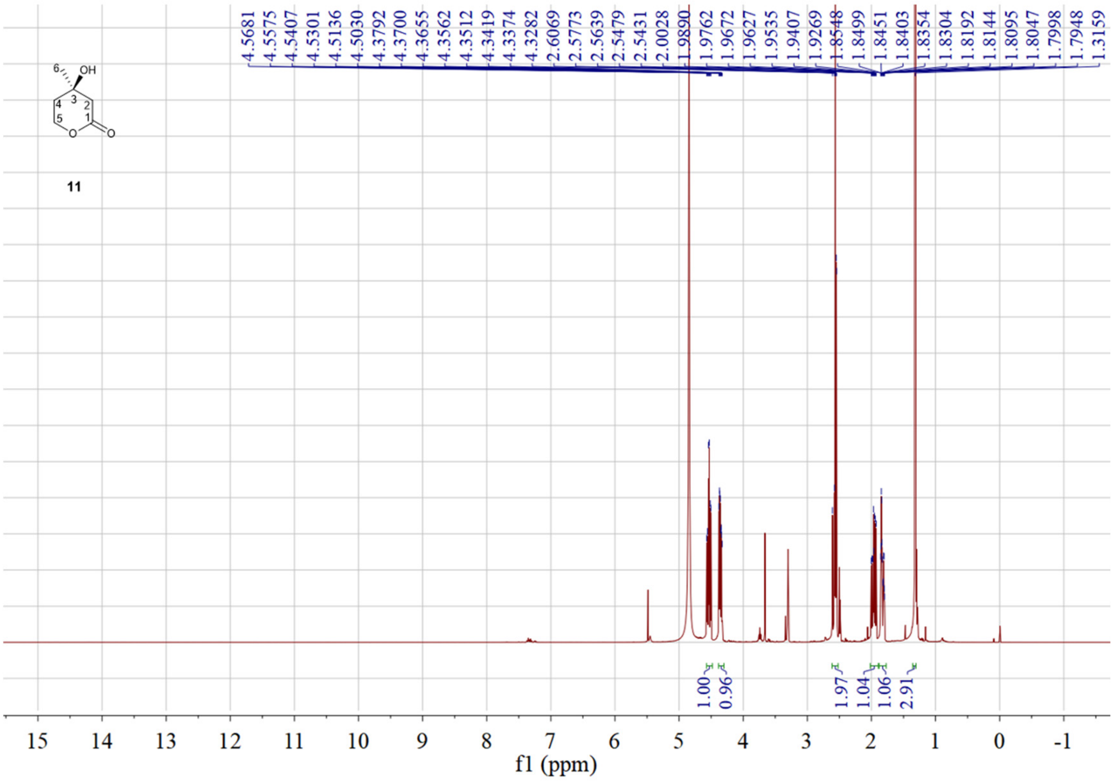

Figure S41. ^{1}H-NMR (400 MHz, CD_3OD) of compound (**11**)

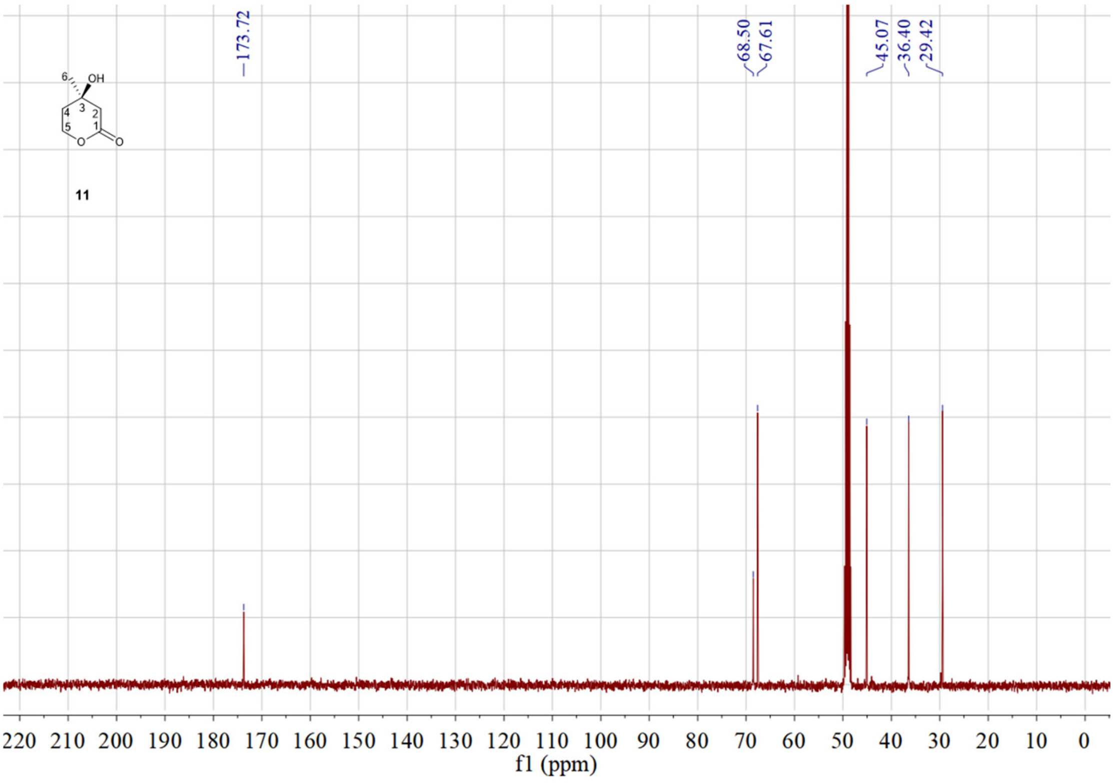

Figure S42. ^{13}C-NMR (101 MHz, CD_3OD) of compound (**11**)

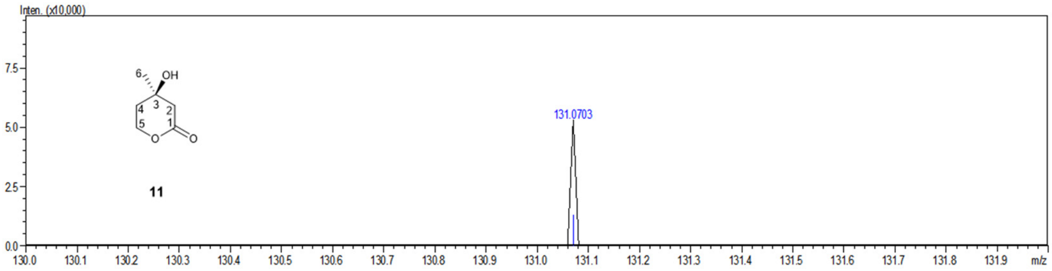

Figure S43. HR-ESI-MS of compound (**11**)

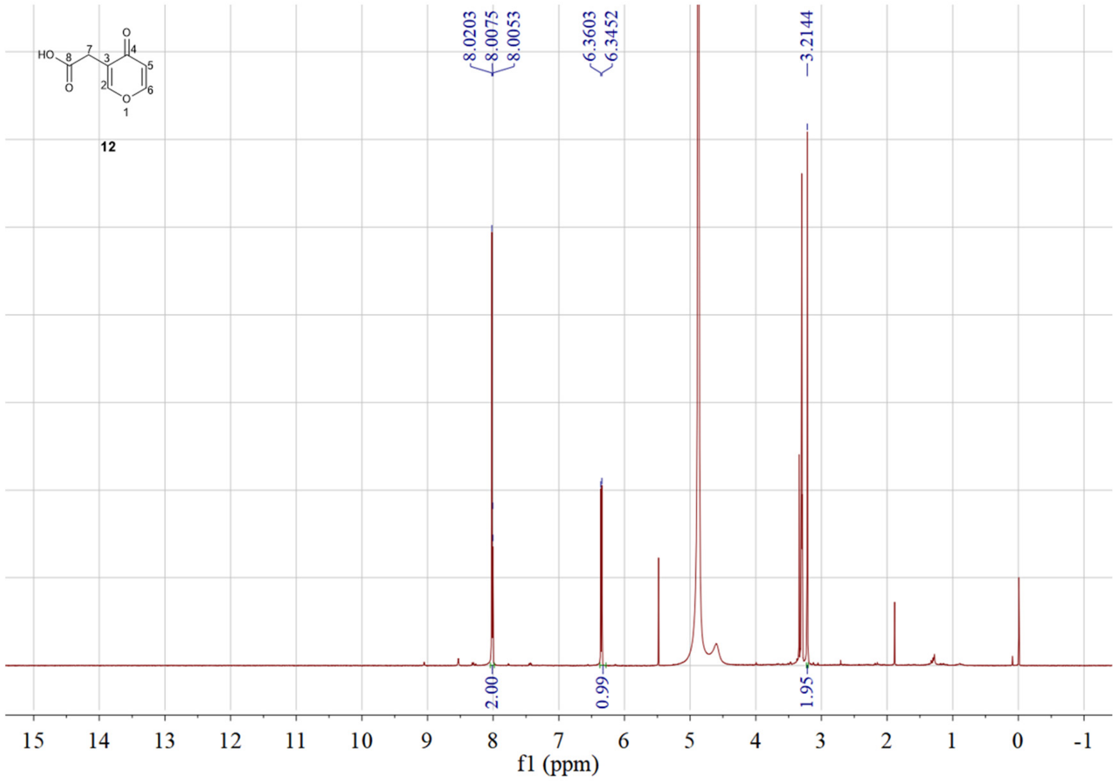

Figure S44. ^{1}H-NMR (400 MHz, CD_3OD) of compound (**12**)

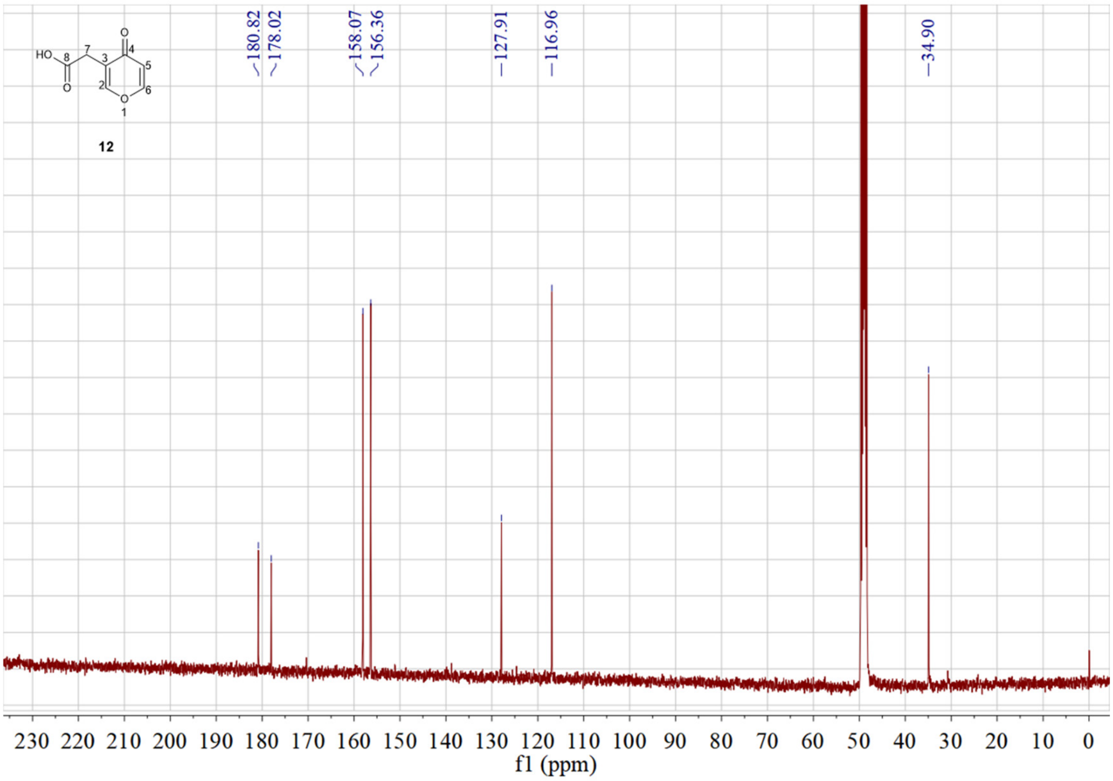

Figure S45. ^{13}C-NMR (101 MHz, CD_3OD) of compound (**12**)

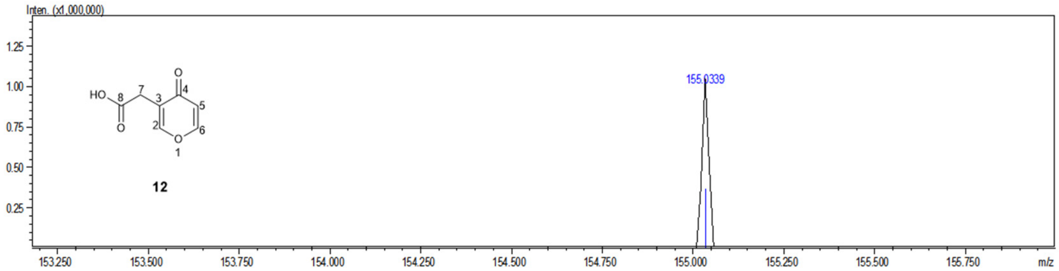

Figure S46. HR-ESI-MS of compound (**12**)

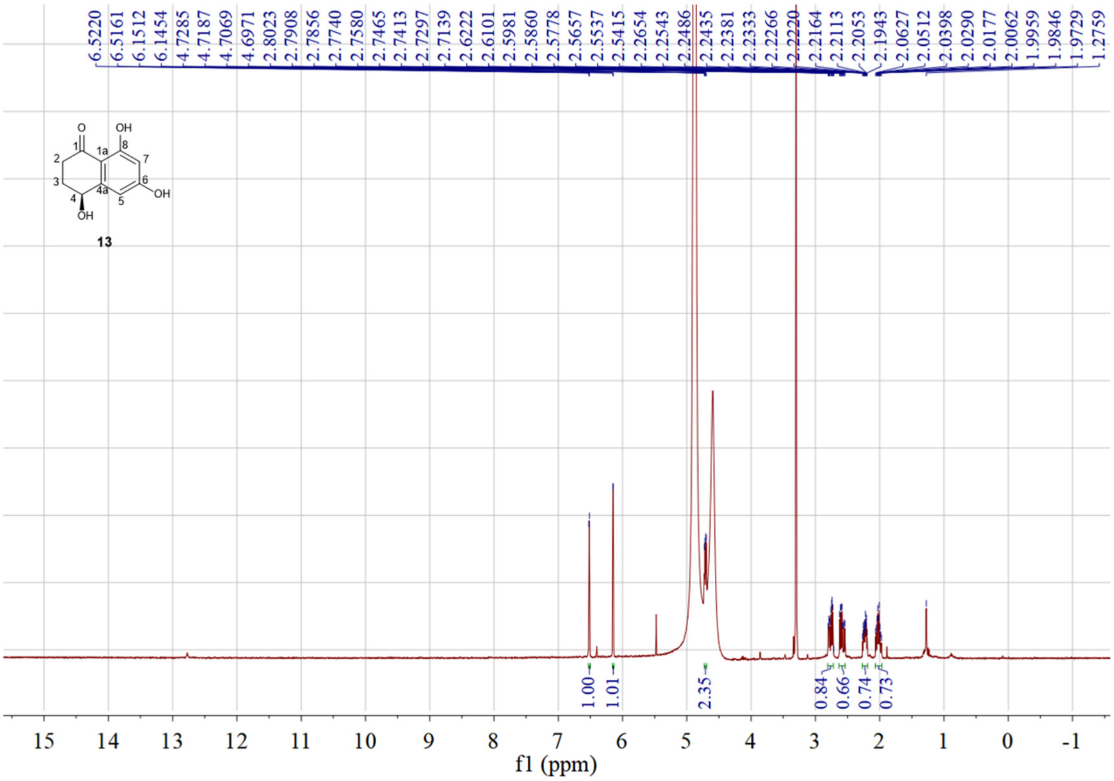

Figure S47. ^{1}H-NMR (400 MHz, CD_3OD) of compound (**13**)

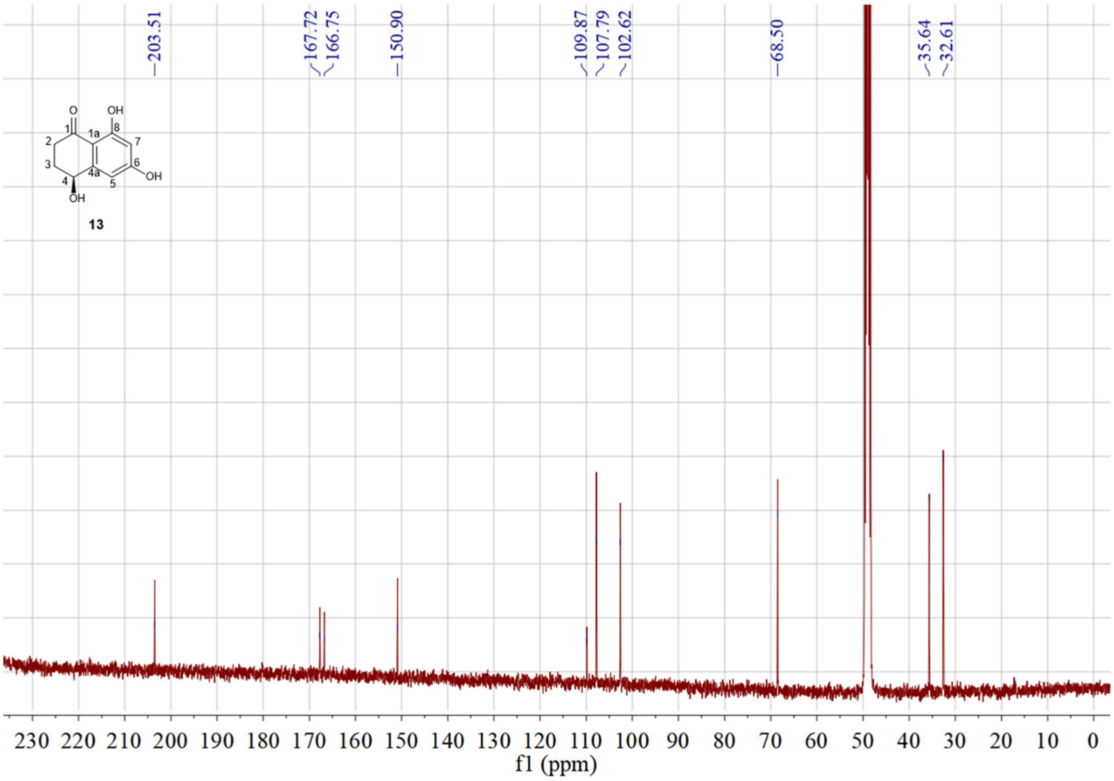

Figure S48. ^{13}C-NMR (101 MHz, CD_3OD) of compound (**13**)

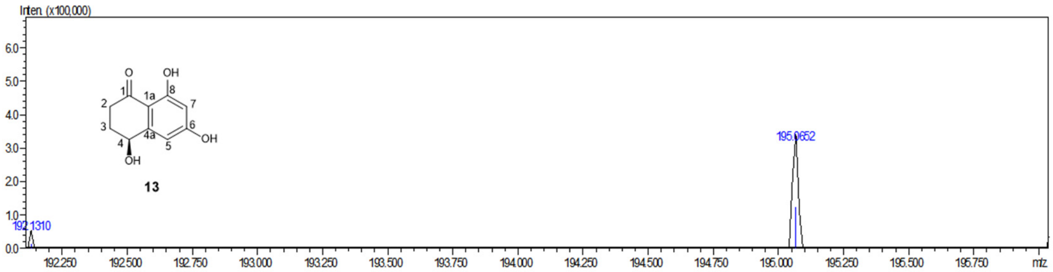

Figure S49. HR-ESI-MS of compound (**13**)

Figure S50. HPLC spectrum for the purity of tested compounds.

HPLC spectrum for the purity of tested compounds. HPLC chromatograms: C18 column (Agilent Technologies 10 mm×250 mm). Solvents: A, H_2O; B, MeOH. Linear gradient: 0 min,10% B; 70 min, 80% B. Temperature 25°C. Flow rate 2 mL/min. UV detection at λ = 210 nm.

Compound 1

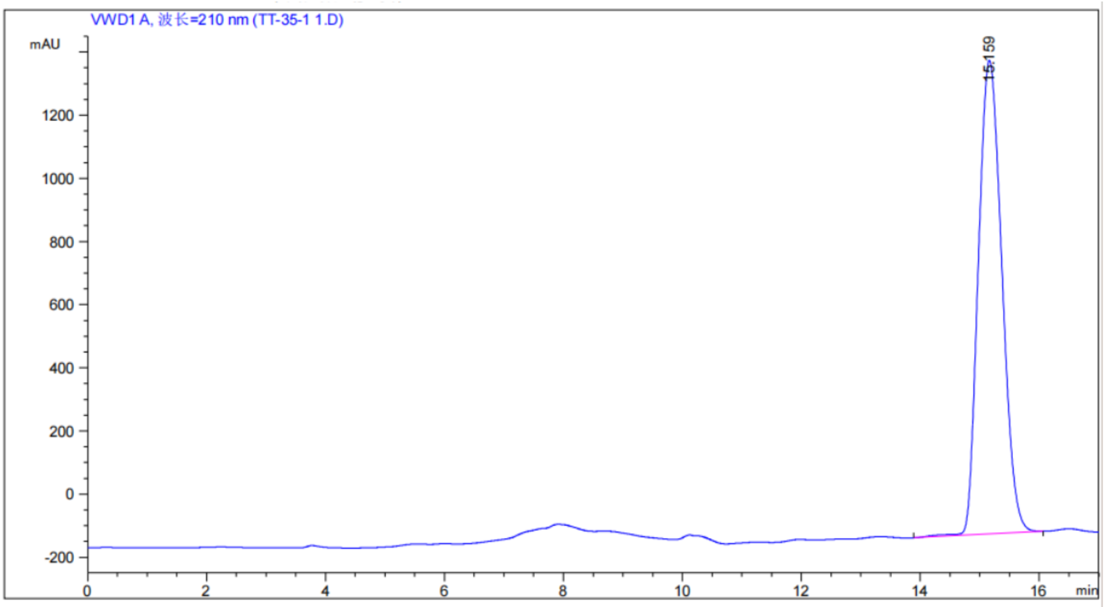

Peak	Retention time	Peak type	Peak width	Peak area	Peak height	Peak area%
#	[min]		[min]	mAU *s	[mAU]	%
1	15.159	BB	0.4249	4.11634e4	1500.20874	100.0000

Compound 2

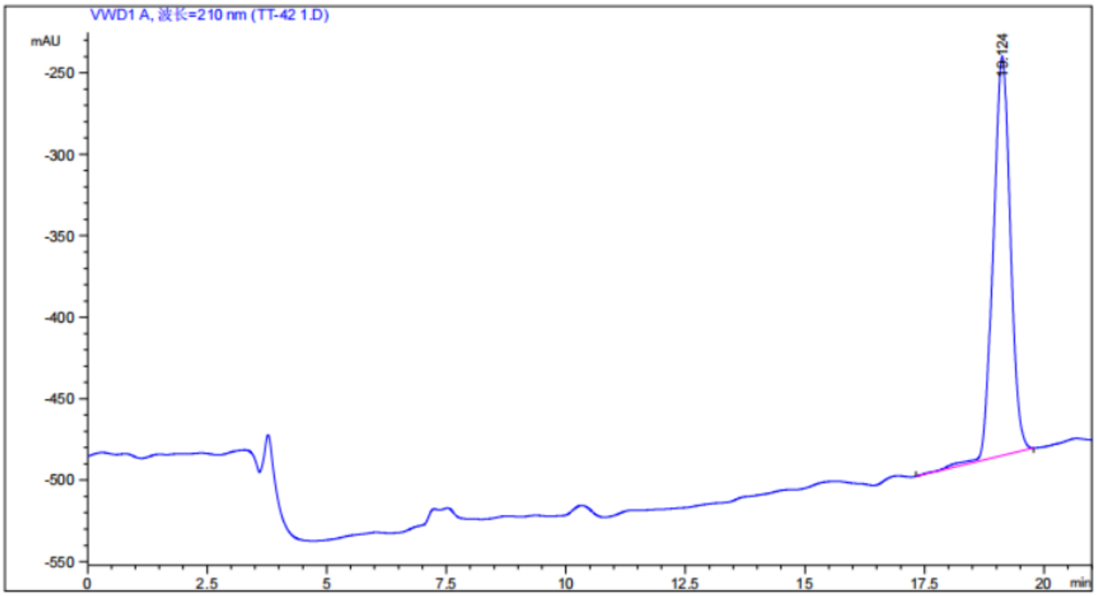

Peak	Retention time	Peak type	Peak width	Peak area	Peak height	Peak area%
#	[min]		[min]	mAU *s	[mAU]	%
1	19.124	BB	0.3732	6126.05273	245.00531	100.0000

Compound 3

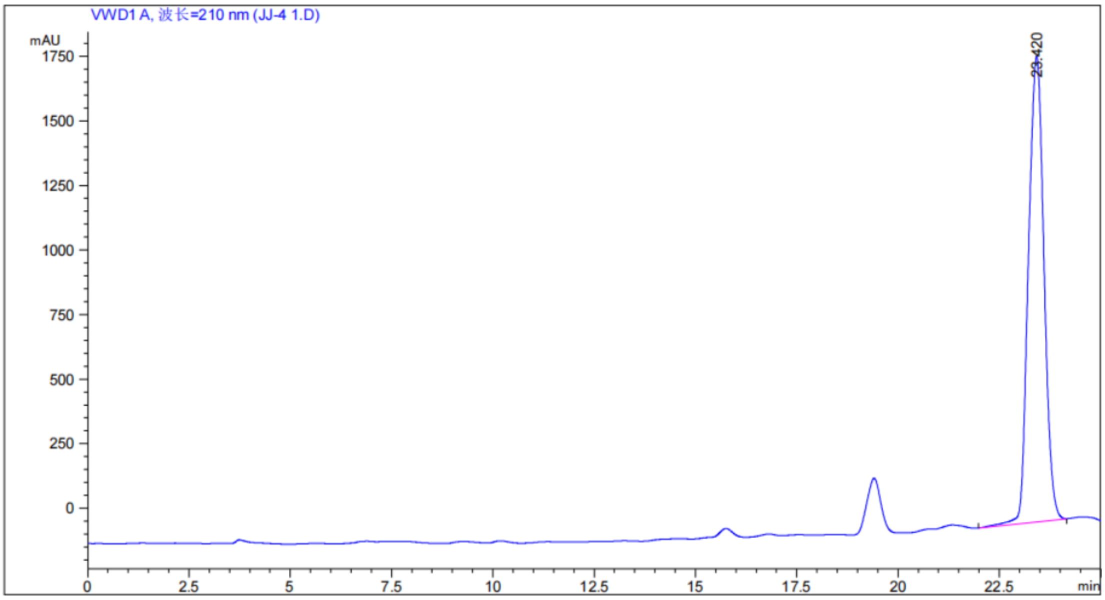

Peak	Retention time	Peak type	Peak width	Peak area	Peak height	Peak area%
#	[min]		[min]	mAU *s	[mAU]	%
1	23.420	BB	0.4258	4.80472e4	1803.96387	100.0000

Compound 4

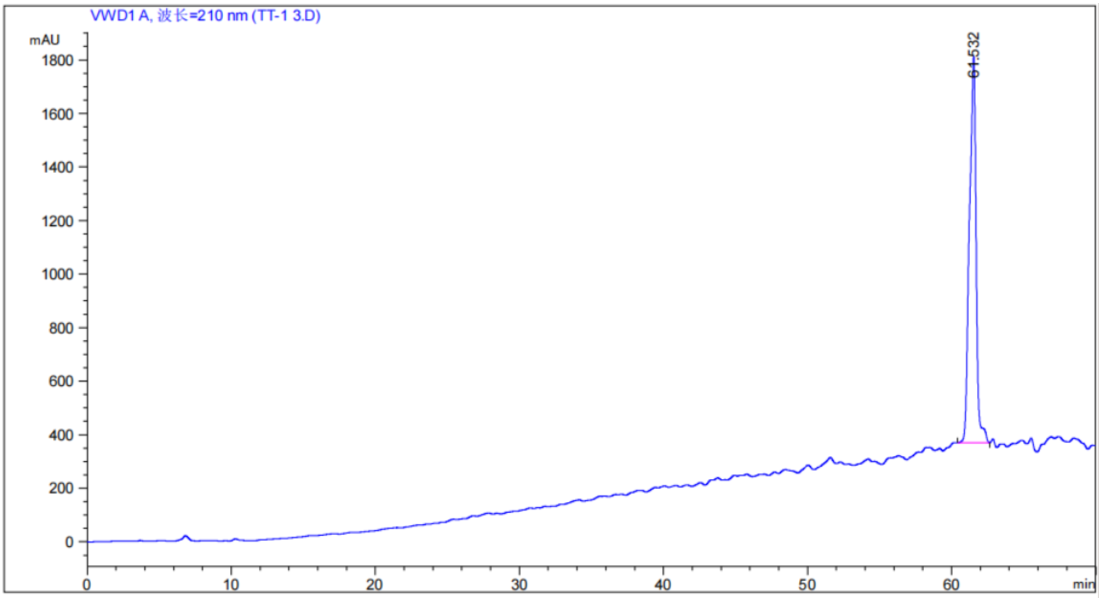

Peak #	Retention time [min]	Peak type	Peak width [min]	Peak area mAU *s	Peak height [mAU]	Peak area% %
1	61.532	BB	0.4553	4.68133e4	1444.29846	100.0000

Compound 5

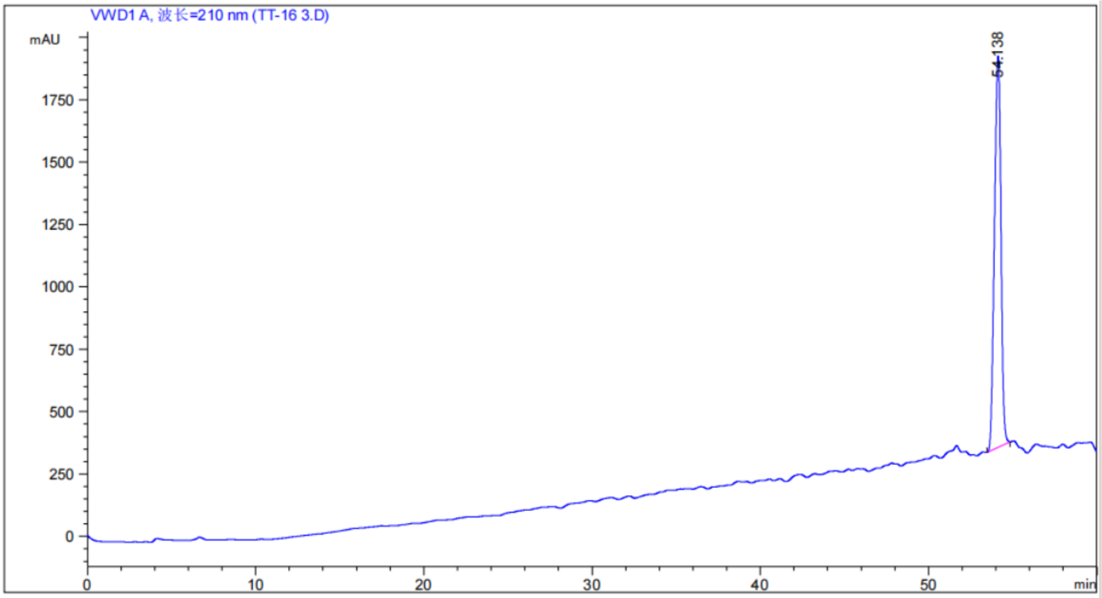

Peak #	Retention time [min]	Peak type	Peak width [min]	Peak area mAU *s	Peak height [mAU]	Peak area% %
1	54.138	BB	0.4139	4.21779e4	1569.80591	100.0000

Compound 6

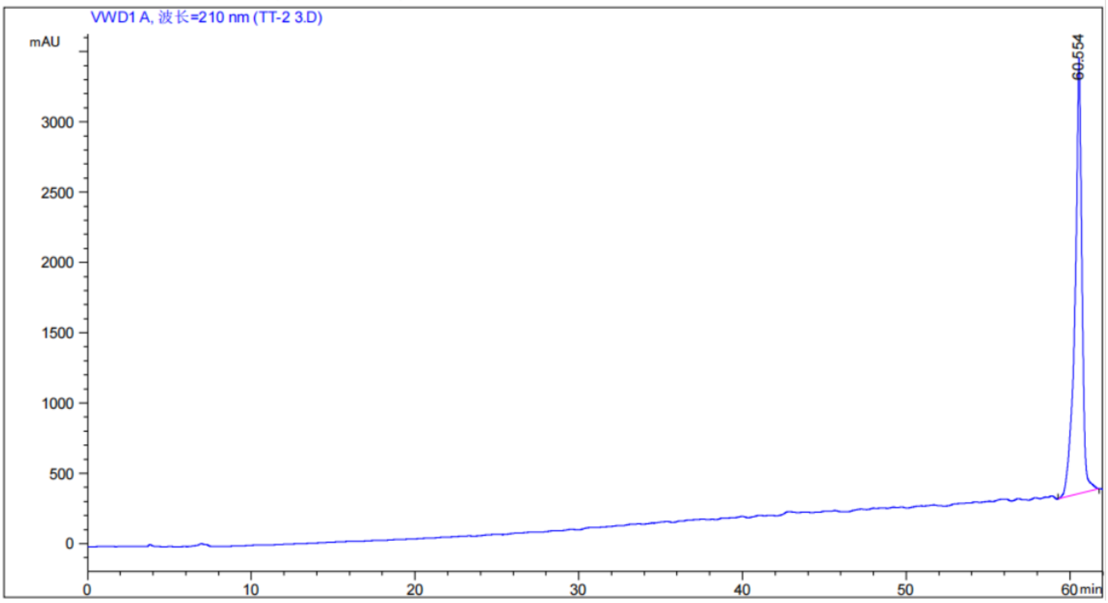

Peak	Retention time	Peak type	Peak width	Peak area	Peak height	Peak area%
#	[min]		[min]	mAU *s	[mAU]	%
1	60.554	BB	0.3899	9.15588e4	3098.46729	100.0000

Compound 7

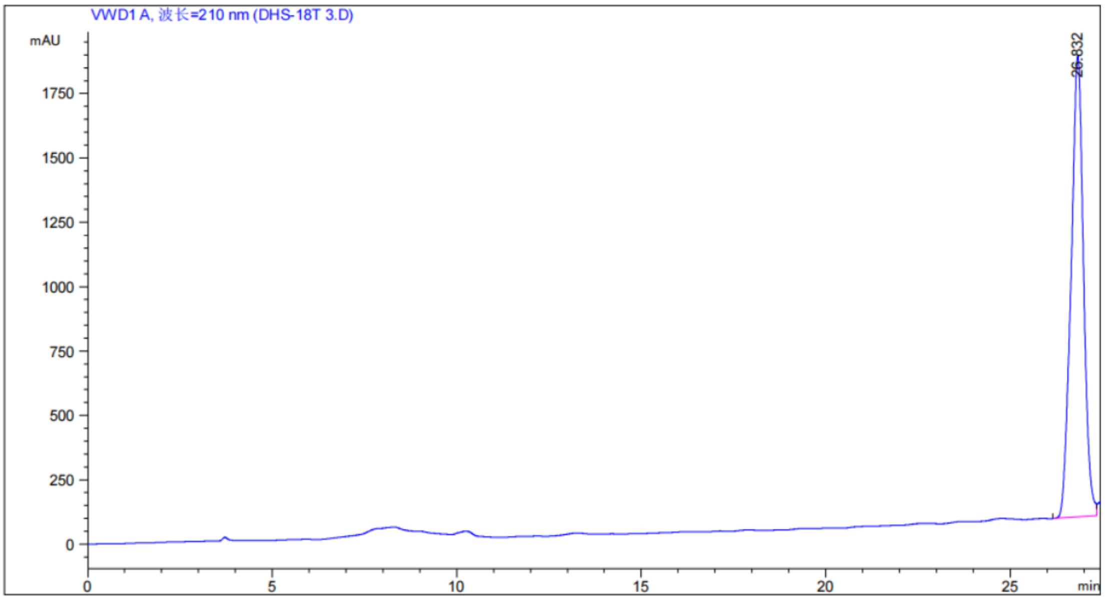

Peak	Retention time	Peak type	Peak width	Peak area	Peak height	Peak area%
#	[min]		[min]	mAU *s	[mAU]	%
1	26.832	BV	0.3426	4.20541e4	1789.70239	100.0000

Compound 8

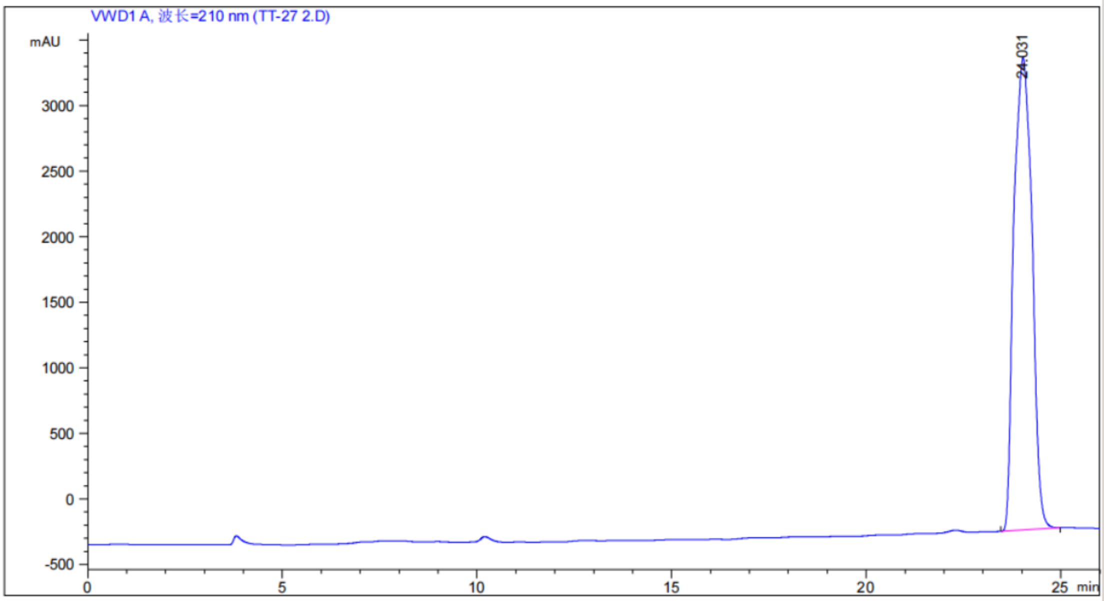

Peak #	Retention time [min]	Peak type	Peak width [min]	Peak area mAU *s	Peak height [mAU]	Peak area% %
1	24.031	BB	0.4304	1.16321e5	3604.51294	100.0000

Compound 9

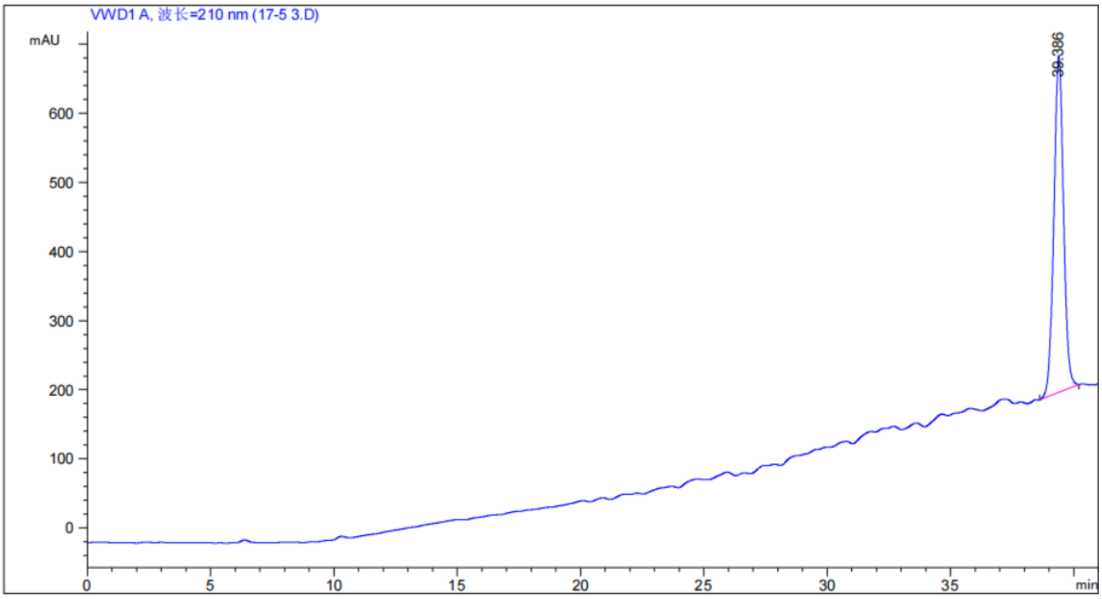

Peak #	Retention time [min]	Peak type	Peak width [min]	Peak area mAU *s	Peak height [mAU]	Peak area% %
1	39.386	BB	0.3963	1.32558e4	486.85516	100.0000

Compound 10

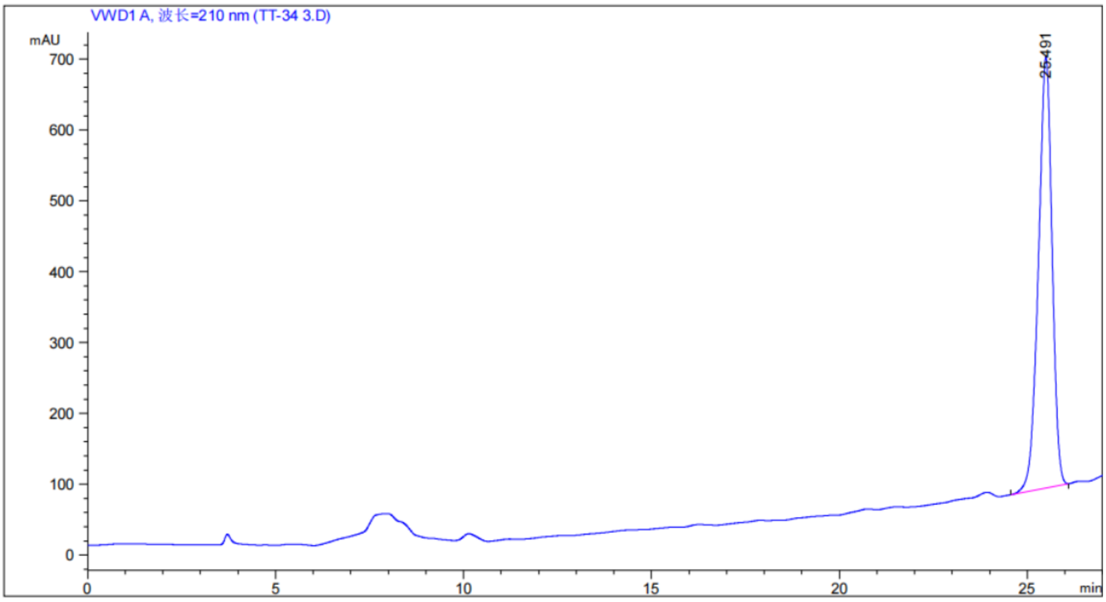

Peak #	Retention time [min]	Peak type	Peak width [min]	Peak area mAU *s	Peak height [mAU]	Peak area% %
1	25.491	BB	0.3771	1.54374e4	609.33905	100.0000

Compound 11

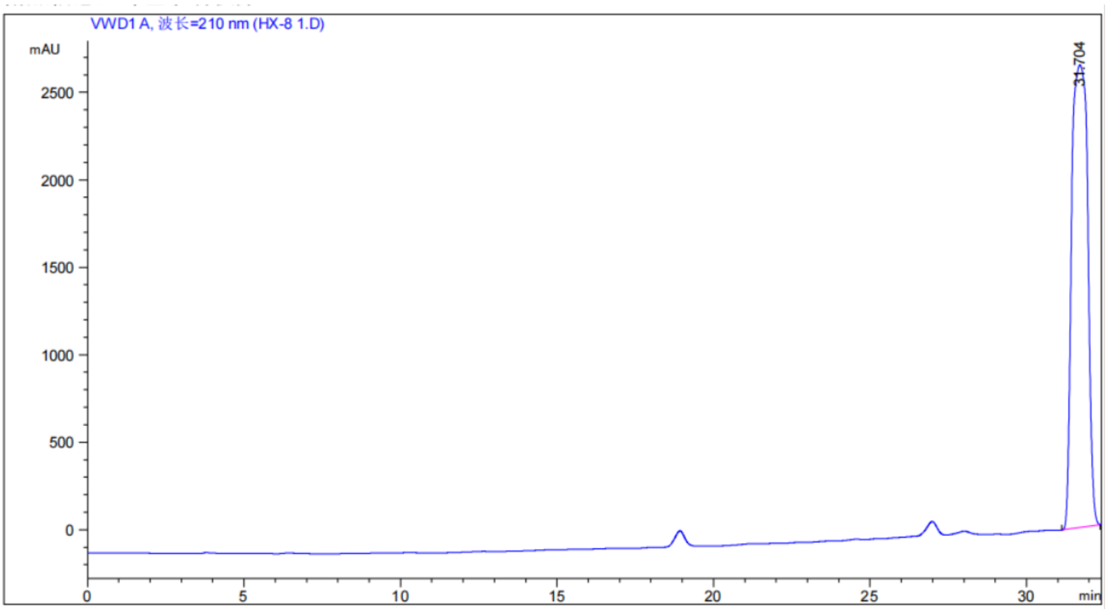

Peak Retention time Peak type Peak width Peak area Peak height Peak area%

#	[min]		[min]	mAU *s	[mAU]	%
1	31.704	BB	0.5826	9.37283e4	2643.36206	100.0000

Compound 12

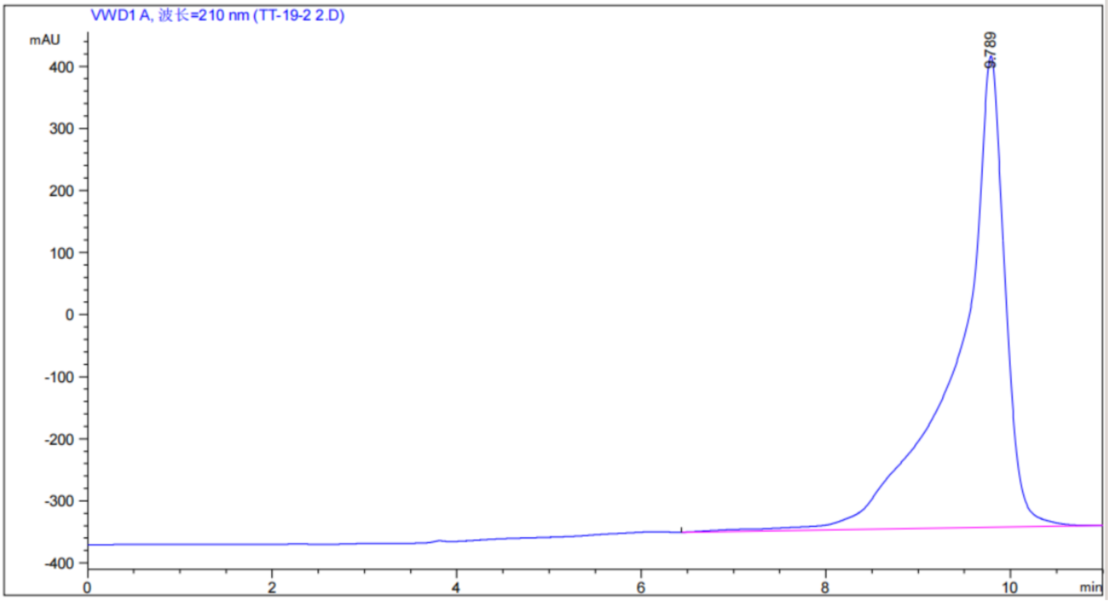

Peak	Retention time	Peak type	Peak width	Peak area	Peak height	Peak area%
#	[min]		[min]	mAU *s	[mAU]	%
1	9.789	BB	0.4804	2.69994e4	759.19965	100.0000

Compound 13

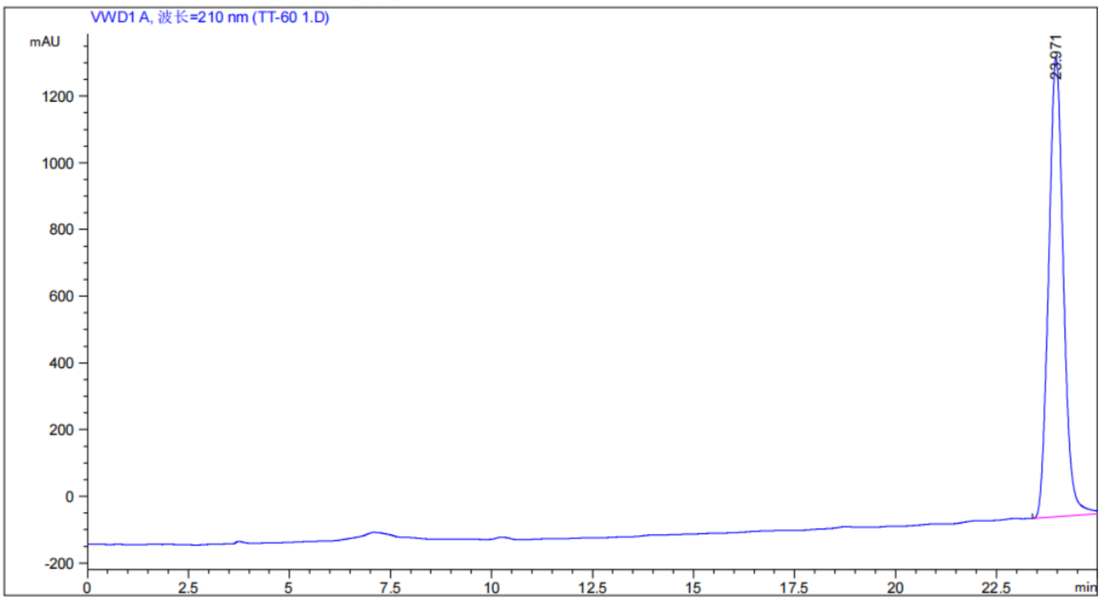

Peak	Retention time	Peak type	Peak width	Peak area	Peak height	Peak area%

```
  #    [min]          [min]   mAU    *s    [mAU    ]        %
----|-------|----|-------|----------|----------|--------|
   1  23.971 BB    0.3787 3.52283e4  1376.41394 100.0000
```

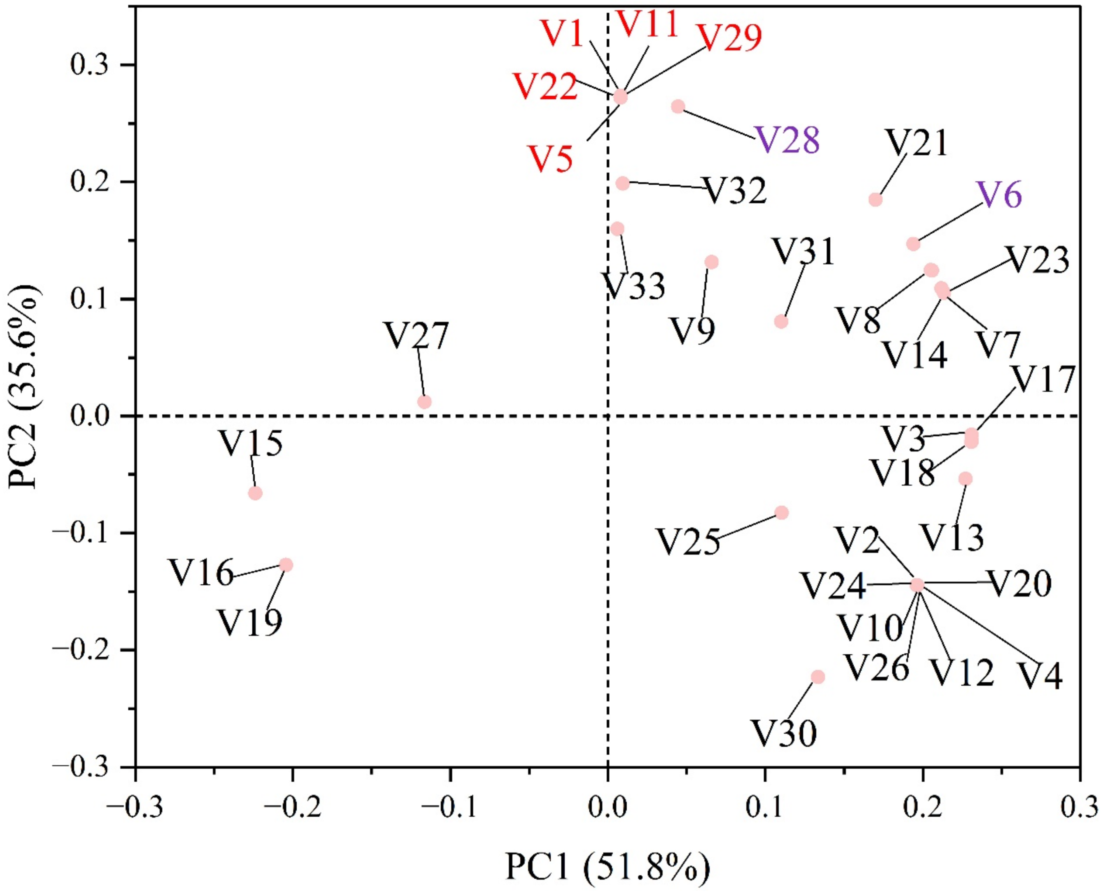

Figure S51. The loadings plot. Red markers (**V1**, **V5**, **V11**, **V22**, and **V29**) represent VOCs newly produced in co-culture, purple markers (**V6** and **V28**) represent VOCs with increased abundance in co-culture.

Table S1 Average colony diameter of left-right configuration (cm)

Day	D(D)				D(H)				H(H)				H(D)			
5	5.70	6.86	6.68	6.94	6.36	6.46	6.36	6.46	4.70	3.98	4.74	4.96	3.72	3.94	3.72	3.94
10	6.92	8.32	8.38	8.48	6.72	8.12	6.72	8.12	5.70	5.70	5.40	5.52	4.04	4.44	4.04	4.44
15	8.36	8.50	7.40	9.00	8.54	6.54	8.54	6.54	5.12	5.52	6.54	5.08	4.74	4.50	4.74	4.50

Table S2 Average colony diameter of center-positioned (cm)

Day	D(D)				D(H)				H(H)				H(D)			
5	7.20	7.28	7.94	8.20	7.54	7.28	7.54	7.28	4.10	4.62	4.36	4.62	3.72	3.94	3.72	3.94
10	8.12	7.60	8.32	7.66	7.76	7.79	7.76	7.79	5.98	6.40	7.28	6.44	4.70	4.44	4.70	4.44
15	9.00	9.00	9.00	9.00	7.60	8.56	7.60	8.56	6.76	6.76	6.48	6.98	5.20	4.74	5.20	4.74

Table S3 Colony growth inhibition ratio under VOC exposure

Day	Left-right configuration	Center-positioned

	D(H)/D(D)				H(D)/H(H)				D(H)/D(D)				H(D)/H(H)			
5	1.12	0.94	0.95	0.93	0.79	0.99	0.78	0.79	1.05	1.00	0.95	0.89	0.91	0.85	0.85	0.85
10	0.97	0.98	0.80	0.96	0.71	0.78	0.75	0.80	0.96	1.03	0.93	1.02	0.79	0.69	0.65	0.69
15	1.02	0.77	1.15	0.73	0.93	0.82	0.72	0.89	0.84	0.95	0.84	0.95	0.77	0.70	0.80	0.68

Table S4 VOCs of DHS-48 and HHL-101 in mono-culture and co-culture

Number	Retention time	Compound name	Molecular formula	Molecular weight	CAS registry number	Peak area D	Peak area H	Peak area H+D
**V1**	8.416	2,4,6-Octatriene, 2,6-dimethyl-	$C_{10}H_{16}$	136.23	673-84-7	-	-	224604669.28
**V2**	8.430	(+)-4-Carene	$C_{10}H_{16}$	136.23	5208-49-1	164399670.21	-	-
**V3**	8.639	o-Cymene	$C_{10}H_{14}$	134.22	527-84-4	547349064.73	-	266197898.97
**V4**	8.641	p-Cymene	$C_{10}H_{14}$	134.22	99-87-6	295144780.84	-	-
**V5**	8.743	(6,6-Dimethylbicyclo[3.1.1]hept-2-en-2-yl)methyl ethyl carbonate	$C_{13}H_{20}O_3$	224.30	-	-	-	465737305.56
**V6**	9.579	.gamma.-Terpinene	$C_{10}H_{16}$	136.23	99-85-4	1055864904.97	-	1161439067.90
**V7**	10.396	Cyclohexene, 1-methyl-4-(1-methylethylidene)-	$C_{10}H_{16}$	136.23	138-86-3	421601704.75	-	391591785.52
**V8**	11.076	Phenylethyl Alcohol	$C_8H_{10}O$	122.16	60-12-8	286546991.34	68277199.68	279801178.66
**V9**	12.988	3-Cyclohexen-1-ol, 4-methyl-1-(1-methylethyl)-, (R)-	$C_{10}H_{18}O$	154.25	562-74-3	2518561092.15	-	1720066864.46
**V10**	13.145	Dill ether	$C_{10}H_{16}O$	152.23	74410-10-9	38966161.70	-	-
**V11**	13.274	.alpha.-Terpineol	$C_{10}H_{18}O$	154.25	98-55-5	-	-	8169745.32
**V12**	13.662	p-Menth-2-en-7-ol, cis-	$C_{10}H_{18}O$	154.25	19898-86-3	8214353.69	-	-
**V13**	14.720	Benzene, 1-methoxy-4-methyl-2-(1-methylethyl)-	$C_{11}H_{16}O$	164.24	250-712-2	45427926.56	-	16273282.10

**V14**	15.091	Carvenone	$C_{10}H_{16}O$	152.23	-	14908325.53	-	14908325.53
**V15**	16.349	Naphthalene, decahydro-4a-methyl-1-methylene-7-(1-methylethylidene)-, (4aR-trans)-	$C_{15}H_{24}$	204.35	138874-69-8	-	21997662.85	5495181.67
**V16**	18.686	Cyclohexane, 1-ethenyl-1-methyl-2,4-bis(1-methylethenyl)-, [1S-(1.alpha.,2.beta.,4.beta.)]-	$C_{15}H_{24}$	204.35	674819-48-8	-	5810453.57	-
**V17**	18.814	Longifolene	$C_{15}H_{24}$	204.35	475-20-7	18610567.80	-	8542099.81
**V18**	19.793	1,5,9,9-Tetramethyl-2-methylene-spiro[3.5]non-5-ene	$C_{14}H_{22}$	190.32	-	25336960.01	-	11435184.63
**V19**	20.209	Dimethyl phthalate	$C_{10}H_{10}O_4$	194.18	131-11-3	-	10521597.47	-
**V20**	20.559	2H-3,9a-Methano-1-benzoxepin, octahydro-2,2,5a,9-tetramethyl-, [3R-(3.alpha.,5a.alpha.,9.alpha.,9a.alpha.)]-	$C_{15}H_{26}O$	222.37	20053-66-1	12223459.80	-	-
**V21**	21.026	Aristolochene	$C_{15}H_{24}$	204.35	26620-71-3	85075852.67	-	116387859.85
**V22**	21.233	Naphthalene, 1,2,3,5,6,7,8,8a-octahydro-1,8a-dimethyl-7-(1-methylethenyl)-, [1R-(1.alpha.,7.beta.,8a.alpha.)]-	$C_{15}H_{24}$	204.35	724783-68-0	-	-	26634391.16
**V23**	21.393	.alpha.-Muurolene	$C_{15}H_{24}$	204.35	10208-80-7	26931265.40	2185010.89	24538652.37
**V24**	21.834	1,1,6,8-Tetramethyl-1,2-dihydro-naphthalene	$C_{14}H_{18}$	186.29	-	10396637.94	-	-
**V25**	21.943	Naphthalene, 1,2,3,5,6,8a-hexahydro-4,7-dimethyl-1-(1-	$C_{15}H_{24}$	204.35	483-76-1	6453993.06	-	-

		methylethyl)-, (1S-cis)-						
**V26**	23.478	4H-Inden-4-one, 1,2,3,5,6,7-hexahydro-1,1,2,3,3-pentamethyl-	$C_{14}H_{22}O$	206.32	33704-61-9	124179965.80	-	-
**V27**	23.653	Hexadecane	$C_{16}H_{34}$	226.44	544-76-3	-	7368187.67	11024292.40
**V28**	25.890	Heptadecane	$C_{17}H_{36}$	240.47	629-78-7	12146319.73	10027798.28	17957525.71
**V29**	26.010	Hexadecane, 2,6,10-trimethyl-	$C_{19}H_{40}$	268.52	55000-52-7	-	-	20802626.67
**V30**	26.012	7-Methyl-octadecane	$C_{19}H_{40}$	268.52	26741-16-2	13153964.63	5140755.00	-
**V31**	31.423	Phenanthrene, 7-ethenyl-1,2,3,4,4a,4b,5,6,7,9,10,10a-dodecahydro-1,1,4a,7-tetramethyl-, [4aS-(4a.alpha.,4b.beta.,7.beta.,10a.beta.)]-	$C_{20}H_{32}$	272.47	1686-56-2	65397428.47	-	24268359.39
**V32**	31.874	Hexadecanoic acid, ethyl ester	$C_{18}H_{36}O_2$	284.48	628-97-7	-	-	36975351.57
**V33**	34.942	Linoleic acid ethyl ester	$C_{20}H_{36}O_2$	308.50	544-35-4	-	-	3064342.40

Table S5 Vip score values (> 1) of VOCs of DHS-48 and HHL-101 in mono-culture and co-culture

Number	VIP value
**V29**	1.12477
**V5**	1.1198
**V11**	1.11928
**V22**	1.11854
**V1**	1.11813
**V28**	1.10365
**V30**	1.0904
**V21**	1.07027
**V20**	1.05522
**V12**	1.05514
**V4**	1.05486
**V10**	1.05472
**V26**	1.05463

**V2**	1.05376
**V24**	1.05345
**V6**	1.05145
**V16**	1.0472
**V19**	1.04658
**V14**	1.04584
**V8**	1.04398
**V7**	1.041
**V23**	1.04085
**V13**	1.03161
**V15**	1.02939
**V17**	1.02793
**V18**	1.02768
**V3**	1.02658

Table S6 Average colony diameter dynamics of HHL-101 after 10 days on 14 cm PDA plate， with **5**/**6** (0, 50 μM) restricted to the central 1 cm region.

Day	Blank control 0 μM					Compd **5** 50 μM					Compd **6** 50 μM				
	N1	N2	N3	Mean	SD	N1	N2	N3	Mean	SD	N1	N2	N3	Mean	SD
1	2.0	2.1	2.0	2.03	0.06	1.7	1.9	2.0	1.87	0.15	1.9	1.7	1.8	1.80	0.10
2	3.4	3.6	3.7	3.57	0.15	3.1	3.4	3.4	3.30	0.17	3.2	3.4	3.1	3.23	0.15
3	4.0	4.1	4.2	4.10	0.10	4.0	4.0	4.1	4.03	0.06	3.8	3.8	3.9	3.83	0.06
4	5.5	5.5	5.5	5.50	0.00	5.5	5.5	5.3	5.43	0.12	5.3	5.2	5.3	5.27	0.06
5	6.0	6.0	6.1	6.03	0.06	6.0	5.8	5.9	5.90	0.10	5.6	5.7	5.6	5.63	0.06
6	7.5	8.0	7.7	7.73	0.25	6.8	6.5	6.7	6.67	0.15	5.7	7.0	5.7	6.13	0.75
7	8.5	9.0	8.7	8.73	0.25	7.0	7.0	7.1	7.03	0.06	6.7	6.7	6.7	6.70	0.00
8	9.2	10.0	9.5	9.57	0.40	7.2	7.3	7.2	7.23	0.06	7.0	7.0	7.0	7.00	0.00
9	11.0	11.0	10.0	10.67	0.58	7.5	7.6	7.4	7.50	0.10	7.2	7.0	7.4	7.20	0.20
10	12.0	11.0	12.0	11.67	0.58	7.7	7.8	7.7	7.73	0.06	7.2	7.1	7.2	7.17	0.06

Table S7 Average colony diameter dynamics of HHL-101 in 9 cm PDA plates treated with compounds **5**/**6** (0, 50 μM)

Day	Blank control 0 μM					Compd **5** 50 μM					Compd **6** 50 μM				
	N1	N2	N3	Mean	SD	N1	N2	N3	Mean	SD	N1	N2	N3	Mean	SD
1	0.9	0.8	0.9	0.87	0.06	0.7	0.7	0.6	0.67	0.06	0.5	0.6	0.5	0.52	0.03
2	2.5	2.4	2.4	2.43	0.06	1.5	1.6	1.5	1.53	0.06	1.2	1.2	1.2	1.18	0.03
3	2.9	2.8	2.8	2.83	0.06	2.0	2.1	2.5	2.20	0.26	1.8	1.9	1.9	1.83	0.03
4	4.2	4.1	4.0	4.10	0.10	3.4	3.5	3.5	3.47	0.06	2.3	2.3	2.3	2.27	0.03
5	4.7	4.6	4.8	4.70	0.10	4.2	4.3	4.5	4.33	0.15	2.5	2.5	2.3	2.42	0.10
6	5.5	5.6	5.5	5.53	0.06	5.0	5.1	5.5	5.20	0.26	2.7	2.6	2.5	2.58	0.08

7	6.7	6.8	6.7	6.73	0.06	5.5	5.6	5.5	5.53	0.06	3.1	2.8	2.7	2.82	0.21
8	7.0	7.0	7.0	7.00	0.00	5.8	5.7	5.7	5.73	0.06	3.3	3.0	2.9	3.02	0.21
9	7.3	7.4	7.3	7.33	0.06	6.0	6.2	6.0	6.07	0.12	3.3	3.1	3.0	3.13	0.15
10	7.5	7.4	7.4	7.43	0.06	6.0	6.2	6.1	6.10	0.10	3.3	3.1	3.0	3.13	0.15

Table S8. Gibbs free energies[a] and equilibrium populations[b] of low-energy conformers of 5*S*,6*S*, 8*S*, 8a*R*, 9*S*, 10a*S*-**1**.

Conformers	In MeOH	
	G[a]	*P* (%)[b]
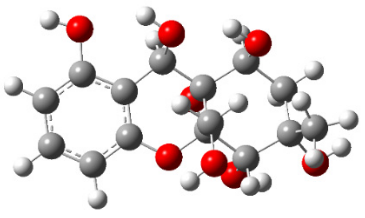 **1-1**	-743674.96307247	23.34
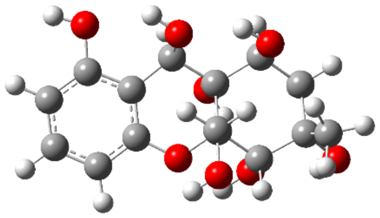 **1-2**	-743675.6426658	73.58
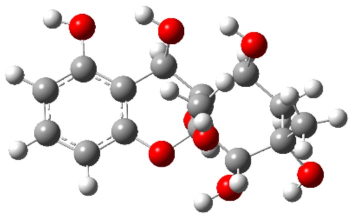 **1-3**	-743673.76327335	3.08

[a]B3LYP/6-31G(d,p), in kcal/mol. [b]From *G* values at 298.15K.

Table S9. Cartesian coordinates for the low-energy reoptimized MMFF conformers of 5*S*,6*S*, 8*S*, 8a*R*, 9*S*, 10a*S*-**1** at B3LYP/6-31G(d,p) level of theory in gas

Conformer **1-1**

**1-1**		Standard Orientation (Ångstroms)			
Center number	Atomic number	Atomic Type	X	Y	Z
1.	6.	0.	-0.319994	4.664304	0.013564
2.	6.	0.	0.207104	4.438756	1.288708
3.	6.	0.	0.433807	3.148374	1.750515
4.	6.	0.	0.112952	2.057647	0.930275
5.	6.	0.	-0.457613	2.248901	-0.334491

6.	6.	0.	-0.642269	3.565702	-0.779165
7.	8.	0.	0.385844	0.817095	1.436064
8.	6.	0.	0.403758	-0.298361	0.498204
9.	6.	0.	-0.863434	-0.218106	-0.398837
10.	6.	0.	-0.894991	1.093985	-1.205795
11.	6.	0.	0.354536	-1.562396	1.394233
12.	6.	0.	0.110924	-2.889127	0.606122
13.	6.	0.	-1.077220	-2.739115	-0.368901
14.	6.	0.	-1.027664	-1.497320	-1.269437
15.	8.	0.	-0.103671	0.996007	-2.407074
16.	8.	0.	-1.147629	3.693704	-2.055418
17.	8.	0.	-0.008642	-1.680250	-2.243256
18.	6.	0.	1.365164	-3.457750	-0.065808
19.	8.	0.	-0.708294	-1.388163	2.340799
20.	6.	0.	1.756930	-0.181415	-0.244364
21.	8.	0.	-2.029793	-0.102897	0.414309
22.	8.	0.	2.846193	-0.267006	0.663132
23.	8.	0.	-0.264851	-3.799050	1.673579
24.	1.	0.	-0.472498	5.675074	-0.356178
25.	1.	0.	0.456890	5.285830	1.920241
26.	1.	0.	0.857125	2.959652	2.730703
27.	1.	0.	-1.945341	1.241281	-1.485185
28.	1.	0.	1.306372	-1.641612	1.930333
29.	1.	0.	-1.143439	-3.627031	-1.009276
30.	1.	0.	-1.995742	-2.682733	0.221046
31.	1.	0.	-2.010559	-1.396961	-1.756296
32.	1.	0.	-0.256410	1.817100	-2.897289
33.	1.	0.	-1.276951	4.629644	-2.255921
34.	1.	0.	0.061962	-0.834114	-2.719159
35.	1.	0.	1.592125	-2.951580	-1.002812
36.	1.	0.	2.227058	-3.407354	0.604314
37.	1.	0.	1.184079	-4.511451	-0.308794
38.	1.	0.	-0.922918	-2.293590	2.626683
39.	1.	0.	1.770308	0.765184	-0.795991
40.	1.	0.	1.875828	-0.977036	-0.973657
41.	1.	0.	-1.820555	-0.496457	1.285533
42.	1.	0.	2.690322	0.409376	1.338021
43.	1.	0.	-0.612801	-4.603743	1.267726

Conformer **1-2**

**1-2**		Standard Orientation (Ångstroms)			
Center	Atom	Type	X	Y	Z

number	number				
1.	6.	0.	-0.336830	4.602488	-0.015518
2.	6.	0.	0.200878	4.382729	1.255125
3.	6.	0.	0.434271	3.092860	1.717536
4.	6.	0.	0.107123	2.002337	0.901963
5.	6.	0.	-0.471198	2.185502	-0.359846
6.	6.	0.	-0.661863	3.501614	-0.805156
7.	8.	0.	0.388659	0.756174	1.405138
8.	6.	0.	0.414866	-0.358363	0.450977
9.	6.	0.	-0.867043	-0.284481	-0.425789
10.	6.	0.	-0.912856	1.027882	-1.230064
11.	6.	0.	0.390735	-1.616911	1.354018
12.	6.	0.	0.107032	-2.959830	0.616230
13.	6.	0.	-1.085039	-2.806460	-0.362821
14.	6.	0.	-1.041134	-1.573252	-1.278092
15.	8.	0.	-0.134579	0.933452	-2.436599
16.	8.	0.	-1.177894	3.626746	-2.075556
17.	8.	0.	-0.025195	-1.757910	-2.255550
18.	6.	0.	1.348261	-3.531266	-0.079160
19.	8.	0.	-0.665940	-1.473639	2.336182
20.	6.	0.	1.757419	-0.228315	-0.304107
21.	8.	0.	-2.024926	-0.153555	0.401661
22.	8.	0.	2.856391	-0.280331	0.594773
23.	8.	0.	-0.214698	-3.932357	1.613430
24.	1.	0.	-0.495548	5.611991	-0.385850
25.	1.	0.	0.454893	5.231738	1.882105
26.	1.	0.	0.871062	2.909622	2.693144
27.	1.	0.	-1.966881	1.173818	-1.496205
28.	1.	0.	1.348364	-1.692309	1.877389
29.	1.	0.	-1.148725	-3.704375	-0.984638
30.	1.	0.	-2.008728	-2.759726	0.221954
31.	1.	0.	-2.025363	-1.474204	-1.762692
32.	1.	0.	-0.303523	1.748453	-2.931621
33.	1.	0.	-1.311742	4.561597	-2.278572
34.	1.	0.	0.026857	-0.923761	-2.752495
35.	1.	0.	1.541895	-3.052965	-1.037604
36.	1.	0.	2.229763	-3.456435	0.563228
37.	1.	0.	1.153629	-4.591276	-0.265421
38.	1.	0.	-0.458708	-0.672706	2.840065
39.	1.	0.	1.754823	0.704517	-0.878505
40.	1.	0.	1.876748	-1.042057	-1.013550
41.	1.	0.	-1.867806	-0.676586	1.208182
42.	1.	0.	2.730237	0.442498	1.225243

43.	1.	0.	-0.788684	-3.490951	2.257068

Conformer **1-3**

**1-3**		Standard Orientation (Ångstroms)			
Center number	Atom number	Type	X	Y	Z
1.	6.	0.	-0.436922	4.610281	-0.038853
2.	6.	0.	0.106637	4.413694	1.234012
3.	6.	0.	0.360298	3.134220	1.712790
4.	6.	0.	0.046979	2.027324	0.913477
5.	6.	0.	-0.536289	2.188083	-0.350723
6.	6.	0.	-0.746239	3.494795	-0.813345
7.	8.	0.	0.334288	0.795145	1.431749
8.	6.	0.	0.401808	-0.315714	0.490769
9.	6.	0.	-0.888204	-0.289210	-0.384707
10.	6.	0.	-0.963482	1.012733	-1.203965
11.	6.	0.	0.418684	-1.571012	1.401268
12.	6.	0.	0.173064	-2.923364	0.671789
13.	6.	0.	-1.040340	-2.817279	-0.286830
14.	6.	0.	-1.055906	-1.593970	-1.216778
15.	8.	0.	-0.188529	0.917128	-2.414786
16.	8.	0.	-1.265572	3.596013	-2.086391
17.	8.	0.	-0.078878	-1.772739	-2.236191
18.	6.	0.	1.424346	-3.448937	-0.041145
19.	8.	0.	-0.642421	-1.450760	2.382110
20.	6.	0.	1.709625	-0.174397	-0.306633
21.	8.	0.	-2.041545	-0.173177	0.449818
22.	8.	0.	2.800063	-0.345854	0.596997
23.	8.	0.	-0.097742	-3.904632	1.676144
24.	1.	0.	-0.610150	5.612668	-0.422127
25.	1.	0.	0.351053	5.274663	1.848572
26.	1.	0.	0.809172	2.967773	2.685423
27.	1.	0.	-2.021142	1.136123	-1.467497
28.					
